# Multiplexed Target Enrichment Enables Efficient and In-Depth Analysis of Antimicrobial Resistome in Metagenomes

**DOI:** 10.1128/spectrum.02297-22

**Published:** 2022-10-26

**Authors:** Yiming Li, Xiaomin Shi, Yang Zuo, Tian Li, Lu Liu, Zhangqi Shen, Jianzhong Shen, Rong Zhang, Shaolin Wang

**Affiliations:** a Beijing Key Laboratory of Detection Technology for Animal-Derived Food Safety, College of Veterinary Medicine, China Agricultural University, Beijing, China; b Guangdong Laboratory for Lingnan Modern Agriculture, Guangzhou, China; c The Second Affiliated Hospital of Zhejiang University, Zhejiang University, Hangzhou, China; Universidad de Buenos Aires, Facultad de Farmacia y Bioqumica

**Keywords:** resistome, targeted amplification, high-throughput sequencing, metagenomics, SNPs

## Abstract

Antibiotic resistance genes (ARGs) pose a serious threat to public health and ecological security in the 21st century. However, the resistome only accounts for a tiny fraction of metagenomic content, which makes it difficult to investigate low-abundance ARGs in various environmental settings. Thus, a highly sensitive, accurate, and comprehensive method is needed to describe ARG profiles in complex metagenomic samples. In this study, we established a high-throughput sequencing method based on targeted amplification, which could simultaneously detect ARGs (*n* = 251), mobile genetic element genes (*n* = 8), and metal resistance genes (*n* = 19) in metagenomes. The performance of amplicon sequencing was compared with traditional metagenomic shotgun sequencing (MetaSeq). A total of 1421 primer pairs were designed, achieving extremely high coverage of target genes. The amplicon sequencing significantly improved the recovery of target ARGs (~9 × 10^4^-fold), with higher sensitivity and diversity, less cost, and computation burden. Furthermore, targeted enrichment allows deep scanning of single nucleotide polymorphisms (SNPs), and elevated SNPs detection was shown in this study. We further performed this approach for 48 environmental samples (37 feces, 20 soils, and 7 sewage) and 16 clinical samples. All samples tested in this study showed high diversity and recovery of targeted genes. Our results demonstrated that the approach could be applied to various metagenomic samples and served as an efficient tool in the surveillance and evolution assessment of ARGs. Access to the resistome using the enrichment method validated in this study enabled the capture of low-abundance resistomes while being less costly and time-consuming, which can greatly advance our understanding of local and global resistome dynamics.

**IMPORTANCE** ARGs, an increasing global threat to human health, can be transferred into health-related microorganisms in the environment by horizontal gene transfer, posing a serious threat to public health. Advancing profiling methods are needed for monitoring and predicting the potential risks of ARGs in metagenomes. Our study described a customized amplicon sequencing assay that could enable a high-throughput, targeted, in-depth analysis of ARGs and detect a low-abundance portion of resistomes. This method could serve as an efficient tool to assess the variation and evolution of specific ARGs in the clinical and natural environment.

## INTRODUCTION

Antimicrobial resistance (AMR) has been considered a pressing global problem, which poses an increasing threat to public health and food security (1). Resistance is frequently observed in almost all commonly used antibiotics, including the so-called “last resort” antibiotics (2) Since 2019, the World Health Organization has classified AMR as one of the top 10 threats to global health (3). Recently, antibiotic-resistance genes (ARGs) have been characterized as emerging environmental contaminants (4). Zhang et al. (5) reported that wastewater, manured soil, animals, and humans are potential reservoirs of ARGs in the natural environment. ARGs could be transferred among different hosts in various environmental media, such as water, soil, and solid manure, through horizontal gene transfer (6, 7). Microbial populations in the environment might be subjected to selective pressure by ARG pollution, which might have consequences for the evolution of antimicrobial resistance (8). Multiple countries and public health organizations agreed that tracking the emergence and prevalence of AMR is critical to improving the treatment of infectious diseases and preventing dissemination in the natural environment (9–11).

Metagenomic shotgun sequencing (MetaSeq) has been widely used to study antibiotic resistance in various fields during the last decade (12–15). A critical challenge of MetaSeq was that antibiotic resistome only accounted for a tiny fraction of the total metagenome (16). Previous studies showed that resistome only accounts for less than 0.1% in feces metagenomic data sets, suggesting that much greater coverage and depth were required for metagenomic sequencing to retrieve AMR (17, 18). Another problem is that the sequencing depth is difficult to assess when the target abundance is extremely low. Furthermore, it is challenging to achieve a sensitive and specific identification of target genes in a complex metagenome background because many variants may exist in the same sample. A targeted sequencing strategy would be a much more efficient way to address these issues through AMR enrichment in metagenomes.

With targeted sequencing, interesting regions of the genome can be easily enriched and sequenced. Targeted approaches can reduce sequencing costs, turnaround time, and data analysis burden (19). There are two main enrichment strategies, the hybrid capture-based method, and the multiplex PCR-based method (amplicon sequencing), respectively. Currently, several hybrid capture-based sequencing methods for ARGs in metagenomes have been established (17, 20–22). Compared with amplicon sequencing, the hybrid capture-based method requires a more complex and longer manual operation time, which may cause unpredictable bias in the sequencing results (19). It was reported that hybrid capture has a certain misalignment rate, and some sequences with lower similarity (~75%) could also be captured (23, 24). Recently, an important trend is the use of multiplex PCR before sequencing. Multiple studies have evaluated the efficiency of amplicon sequencing in human disease characterization and species identification (25, 26). For instance, Ion AmpliSeq technology has been applied to viral gene variants detection, SNP typing in humans, and tuberculosis drug resistance genes, which allows up to 6144 custom amplicons for interesting regions (27–29). Until recently, amplicon sequencing has been mainly used for microbial identification and clinical disease screening. Few studies focused on AMR have been reported.

Herein, we described a customized AmpliSeq panel and sequencing assay targeting multiple ARGs in the metagenome. We found that the established AmpliSeq method could significantly enhance the sensitivity and specificity over conventional MetaSeq analysis in the detection of AMR. Target enrichment could effectively expand the resistome to include thousands of low-abundance ARGs, which are sometimes even more important to public health. This approach could serve as a cost- and time-efficient tool for various studies, including identification, epidemiological, and evolution assessment of ARGs in complex metagenomes.

## RESULTS

### Design and characterization of amplicon sequence panel.

**FIG 1:**
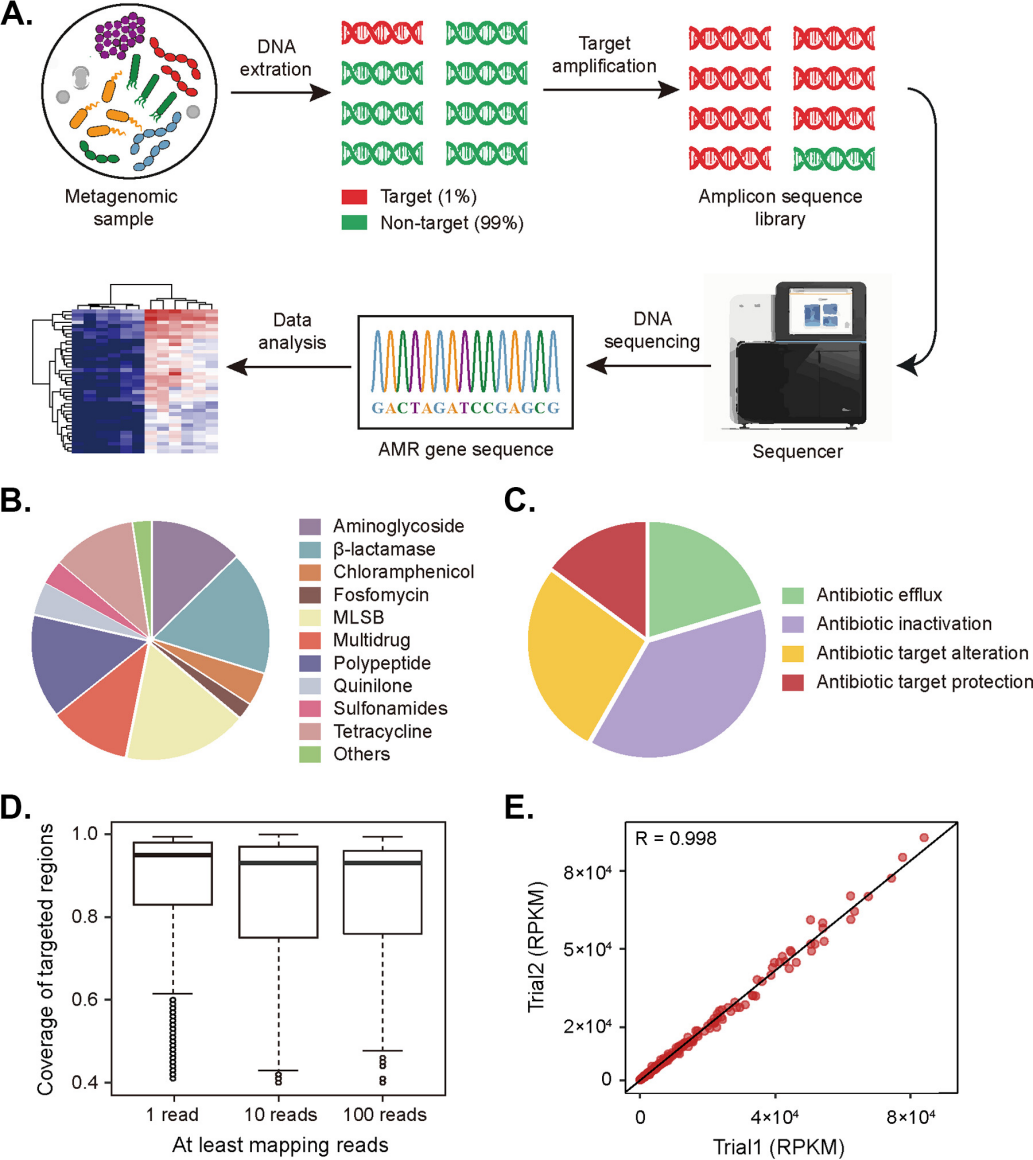
Characteristic of the designed approach. (A) The whole process of amplicon sequencing. The workflow begins with DNA exaction from a metagenomic sample. Targeted sequences only account for less than 1% of the total DNA. Targeted sequences are amplified and then prepared into a sequencing library. The amplified library was sequenced, and reads were analyzed for ARGs by aligning with the sequences in the custom database for further analysis. (B) Classification of ARG determinations selected from Resfinder, CARD, and AMRFinderPlus database (*n* = 251). MLSB, macrolide-lincosamide-streptogramin B. (C) Classification of resistance mechanisms of selected ARG determinations. (D) Percent length of coverage of targeted regions with reads from 6 tested samples (1 versus 10 versus 100 reads). (E) Consistency of methods in different trials. Reads from samples in Trail 2 were library prep and sequenced to the same depth as reads in Trial 1. The reads were mapped to target regions, filtered for mapping quality, and then the number of reads was normalized using reads per kilobase per million (RPKM). Pearson correlation coefficients are shown in the diagram.

A total of 278 target genes were selected, including 251 antibiotic resistance determinants, 8 mobile genetic elements genes, and 19 metal resistance genes, based on Resfinder, CARD, AMRFinderPlus, MGEfinder, and BacMet databases, respectively (Table S1 in Supplemental File 1). The genes targeted by our primers conferred resistance to multiple antibiotics and encoded diverse resistance mechanisms (Fig. 1B and C). A custom AmpliSeq panel was designed based on amplicon sequencing technology. Two primer pools were designed to obtain optimal results for multiplex PCR amplification, which contain 746 and 675 primer pairs, respectively. Each target gene was covered by several amplicons (median [interquartile range, IQR], 5 [3 to 7]), with a fragment size of 125 to 275 bp. Most target genes were 100% covered by amplicons (median, [IQR], 100% [100% to 100%]). In practice, analysis of our sequencing data showed over 75% of target regions were captured by 100 reads or more (Fig. 1D). The results yielded from the AmpliSeq approach were highly reproducible with much less variation among technical replicates because a strong correlation was observed between reads per kb per million reads (RPKM) (*R* = 0.998) and upper quartile (UQ) (*R* = 0.986) for library prepared individually in two trials (Fig. 1E and Fig. S1 and S2 in Supplemental File 1).

### Target amplification enabled better recovery of resistance genes than MetaSeq.

The performance of amplicon sequencing (AmpliSeq) and MetaSeq was compared using 6 feces samples. After stringent quality filtering, the median reads generated from AmpliSeq and MetaSeq methods were 1.73 × 10^6^ (IQR, 1.30 × 10^6^ to 2.30 × 10^6^) reads, and 3.41 × 10^7^ (IQR, 3.37 × 10^7^ to 3.44 × 10^7^) reads, respectively. In all 6 samples, the median percentage of mapping reads (the mapping reads number relative to the total reads number) against the custom database (251 ARGs, 7 mobile genetic elements [MGEs], and 19 metal resistance genes [MRGs]) was 0.0008% (IQR, 0.0004% to 0.0015%) for MetaSeq data set and 92.14% (IQR, 90.55% to 92.83%) for AmpliSeq data set (Table 1). And 9.2 × 10^4^-fold enrichment on average was achieved after target amplification compared with MetaSeq (Table 1).

**TABLE 1:** Comparison of metagenome shotgun sequencing and amplicon sequencing

Sample	Metagenome shotgun sequencing				Amplicon sequencing				Gain^*a*^
	Raw reads	Aligned reads	Percent (%)	Aligned reads per gene^*b*^	Raw reads	Aligned reads	Percent (%)	Aligned reads per gene^*b*^	
CS1	3.4 × 10^7^	690	0.0021	4 (2–10)	1.3 × 10^6^	1.0 × 10^6^	75.79	324.5 (19.75–2245.5)	3.7 × 10^4^
CS2	3.4 × 10^7^	125	0.0004	3.5 (2–4.75)	1.3 × 10^6^	1.3 × 10^6^	98.55	219 (23.5–2134)	2.7 × 10^5^
CS3	3.4 × 10^7^	550	0.0016	5 (2–9)	2.2 × 10^6^	2.0 × 10^6^	92.98	518 (90.5–2823)	5.8 × 10^4^
CS4	3.5 × 10^7^	86	0.0002	3 (2–5)	2.4 × 10^6^	2.2 × 10^6^	92.39	126 (22–463)	3.7 × 10^5^
CS5	3.3 × 10^7^	381	0.0011	4 (2–7)	1.3 × 10^6^	1.1 × 10^6^	90.10	127 (32–432.5)	7.9 × 10^4^
CS6	3.4 × 10^7^	176	0.0005	2 (2–5.75)	3.3 × 10^6^	3.0 × 10^6^	91.88	113 (15 –886)	1.8 × 10^5^
Avg	3.4 × 10^7^	335	0.0010	NA^*c*^	1.9 × 10^6^	1.8 × 10^6^	90.28	NA^*c*^	9.2 × 10^4^

a The results of dividing the on-target percentage of amplicon sequencing by shotgun sequencing.

b These results are represented as median (interquartile range, IQR).

c NA, Not applicable.

**FIG 2:**
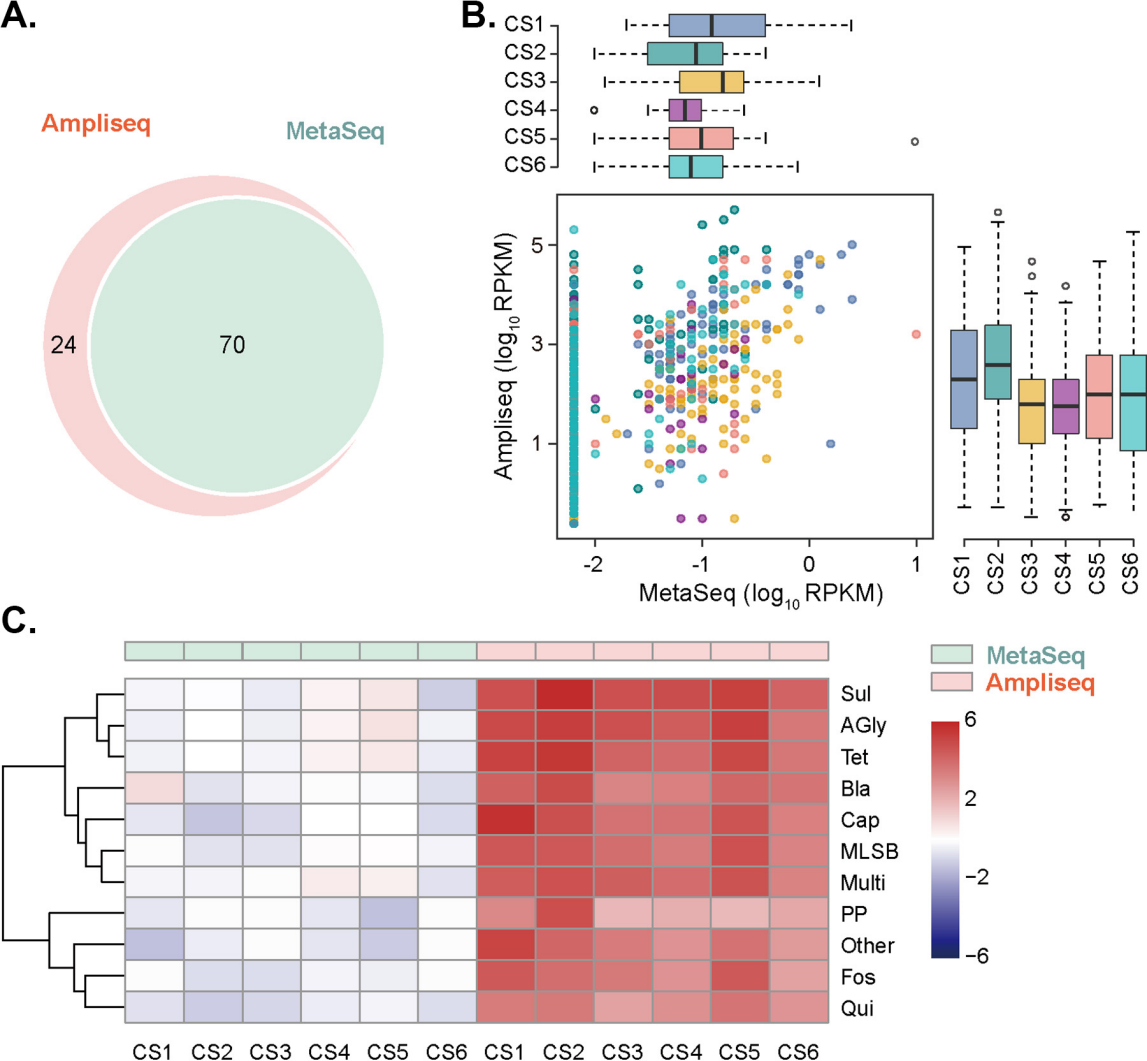
Performance summary. (A) Venn diagrams between AmpliSeq and MetaSeq of differentially detected mapping gene clusters against the custom database in this study. Each mapping gene cluster refers to a set of alleles/genes detected by a set of reads. (B) Reads per kilobase per million (RPKM) of reads mapping to each detected gene between MetaSeq and AmpliSeq. Genes only screened by AmpliSeq are shown in the initial values of the abscissa axis. (C) Abundance (RPKM) of each ARG family between AmpliSeq and MetaSeq. ARGs were classified into 10 families (Sul, sulfonamides; AGly, aminoglycosides; Tet, tetracyclines; Bla, β-lactams; Cap, chloramphenicol; MLSB, macrolide-lincosamide-streptogramin B; Multi, multidrug; PP, polypeptide; Fos, fosfomycin; Qui, quinolone). RPKM was used to normalize read counts and log-transformed to produce the heat map.

The set of variants/genes detected by a set of reads was defined as a “mapping gene cluster” (MGC), which includes hundreds of genes or just one gene (17). A certain number of MGCs captured by AmpliSeq (25.53%, 24/94) against the custom database were not captured by MetaSeq (Fig. 2A). Besides, 80 additional MGCs were only detected by the MetaSeq method, which was not included in the custom panel designed in this study. Reads per kb per million reads (RPKMs) and upper quartile (UQ) normalization were used to demonstrate the gene abundance in total sequencing data, and better recovery of target genes was observed in AmpliSeq compared with MetaSeq (Fig. 2B and Fig. S3A in Supplemental File 1). Furthermore, the abundance of each antibiotic resistance family was analyzed, which demonstrated the enhanced sensitivity of the approach (Fig. 2C and Fig. S3B in Supplemental File 1).

### Target amplification achieved higher gene diversity and highly informative SNPs.

**FIG 3:**
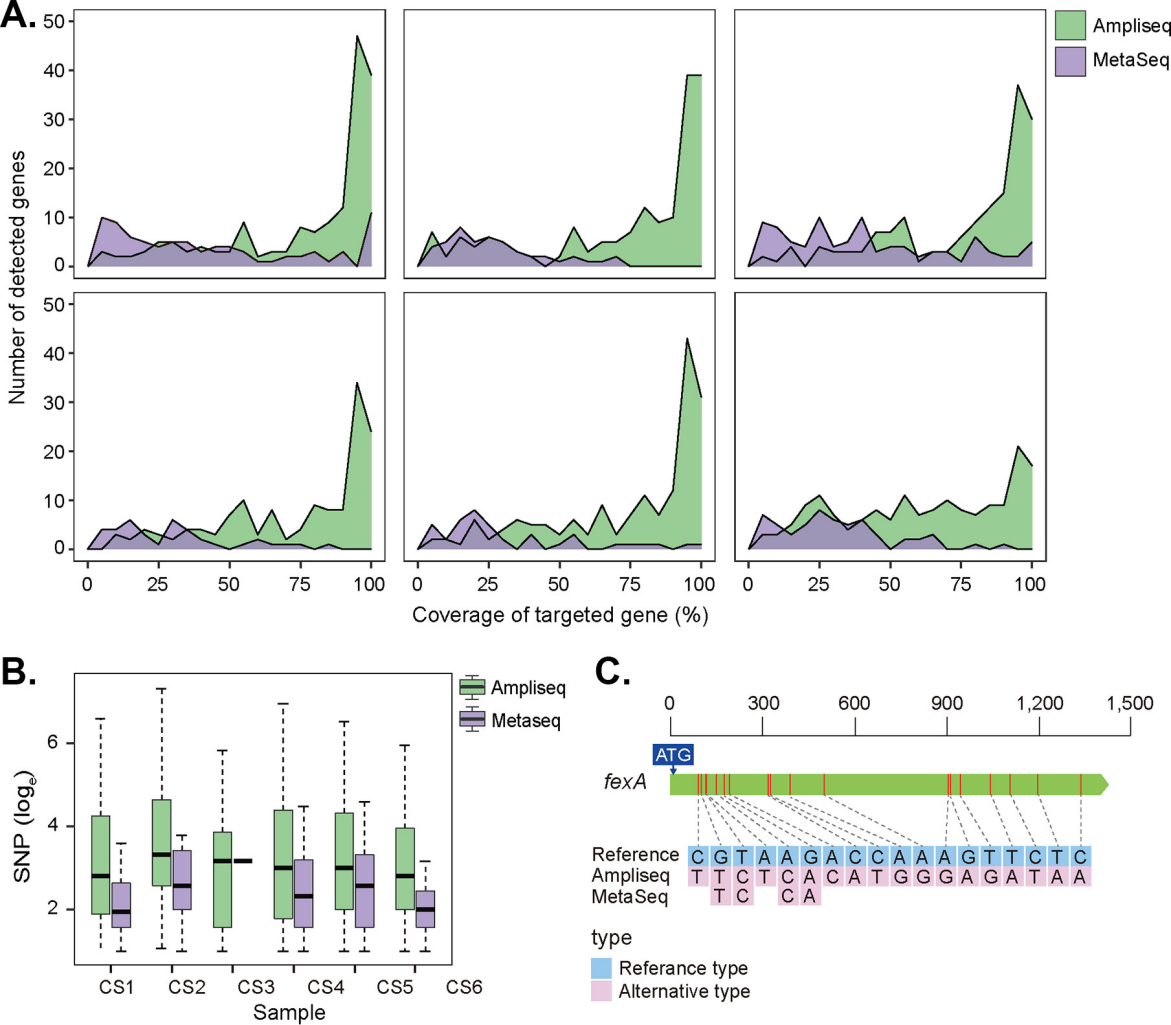
Comparison of AmpliSeq and MetaSeq in gene diversity. (A) Diversity and coverage distribution of target genes. The figures show the comparison of the length coverage distribution of targeted genes between MetaSeq and AmpliSeq in each sample (*n* = 6). (B) Distribution was represented by the density parameter and expressed by the length coverage of each detected gene (abscissa) and the number of detected genes (ordinate). Comparison of single nucleotide polymorphisms (SNPs) distributions between MetaSeq and AmpliSeq in each sample (*n* = 6). Distributions were represented by the number of SNPs detected in each targeted gene in different samples. (C) Nucleotide variant depiction for the *fexA* gene (the full length of which is represented in the figure) across all 12 samples. SNPs identified in AmpliSeq (*n* = 6) and MetaSeq (*n* = 6) were combined, respectively, and exhibited in the figure.

One advantage of amplicon sequencing is to amplify the signal of low-abundance target genes and greatly improves the sensitivity of the method. Higher gene diversity was indeed observed when using AmpliSeq compared to MetaSeq (Fig. 3A). Across all 6 sequenced libraries, we identified 203 unique ARGs. Specifically, the median number of target genes identified were 166.50 (IQR, 160.50 to 170.50) for AmpliSeq and 38 (IQR, 30.25 to 59.25) for MetaSeq, which increased ~4-fold (*P* < 0.01). We defined ARGs with fewer reads than low-quartiles in the MetaSeq method as low-abundance ARGs and observed their performance in the AmpliSeq approach. The median reads mapping to low-abundance ARGs were 1.28 (IQR, 0.71 to 1.42) for AmpliSeq and 3.60 × 10^2^ (IQR, 1.23 × 10^2^ to 1.59 × 10^3^) for MetaSeq, which increased ~283-fold. Besides, compared with MetaSeq, an additional 92 unique genes were identified by AmpliSeq (Table S2 in Supplemental File 1). Cumulatively, reads aligning with these 92 unique genes accounted for only 1.43% of all reads that mapped to the custom database across all 6 sequenced libraries, suggesting that these genes were part of the low-abundance ARGs. These results indicated that the AmpliSeq method performed better in the recovery of low-abundance ARGs in environmental samples. In addition, coverage across target genes was further investigated in our research. Fig. 3A illustrated that most of the gene full-length coverage was over 80% by the AmpliSeq method, while the gene coverage by the MetaSeq method was generally within 50%. The sequencing depth was generally increased by amplicon sequencing, and the number of reads mapped to target genes increased by ~3.1 × 10^3^ times (Fig. 2B) (*P* < 0.01).

In addition, the single nucleotide polymorphisms (SNPs) patterns of AMR genes were compared in all test samples, focusing only on the genes identified in all 12 samples. Our results suggested that targeted amplification before sequencing could significantly elevate SNPs detection in metagenomes (Fig. 3B). Major facilitator superfamily (MFS) antibiotic efflux pump gene, *fexA*, conferring combined resistance to phenicols antibiotics, was identified in all 12 sequenced libraries and selected for further SNP analysis (Fig. 3C). Fifteen more specific SNPs were found in AmpliSeq data set, and the allele frequency was 52.7% on average, which suggested the environment samples may harbor more ARG variants than estimated.

### Application of the designed method in various metagenomic samples.

After the establishment of the method, 48 environmental metagenomic samples (21 feces, 20 soils, and 7 sewage samples) from animal farms and 16 clinical feces samples were analyzed. An average of 1.12 × 10^6^ paired reads (median [IQR], 1.10 × 10^6^ [5.92 × 10^5^ to 1.48 × 10^6^]) were obtained from all 64 sequencing libraries, and the concentrations of the constructed library and the amount of sequence data generated were consistent among different sample types. Analysis of ARGs abundance (expressed in RPKMs and UQ) showed an improved recovery of targeted genes (median 9.43 × 10^2^ reads [IQR, 1.67 × 10^2^ to 6.72 × 10^3^] normalized by RPKM, median 1.14 × 10^2^ reads [IQR, 1.93 × 10^2^ to 6.55 × 10^3^] normalized by UQ) and most ARGs detected achieved 80% coverage of the gene in full-length (unpublished data). In addition, all samples tested in this study showed a high diversity of target genes (ranging from 65 to 147).

**FIG 4:**
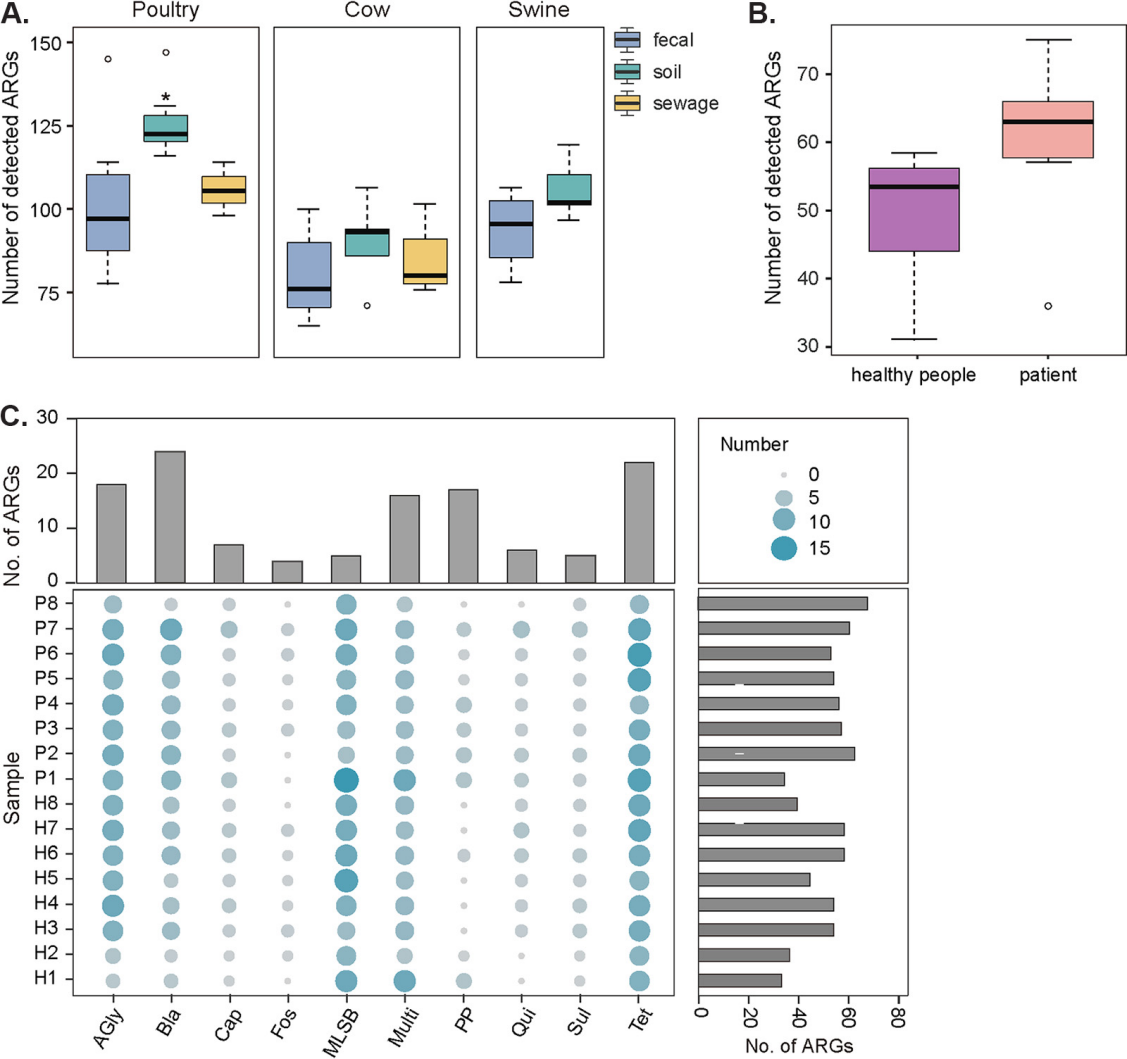
Application of the method in environmental and clinical samples. (A) Diversity of ARGs among different environmental samples. Diversity was measured as the number of targeted genes detected in each sample. (B) Comparison of the diversity of ARGs between healthy individuals and patients. (C) Statistical combination diagrams of ARGs diversity of clinical samples. Balloon plot depicting the number of targeted ARGs detected in each sample (*n* = 16). The size of the circles represented the number of detected ARGs and the details were annotated in the legend. H1 to H8, healthy individuals. P1 to P8, patients.

Our metagenomic analysis shows sample types may have an impact on ARGs diversity. For instance, human gut metagenome contained a lower ARGs diversity than environmental samples from farms (*P* < 0.05) (Fig. 4A and B), suggesting the environment is an important reservoir of ARGs. Different AMR patterns were presented among different patients and healthy individuals, for example, the median number of ARGs identified was 63 (IQR, 57.75 to 66) in patients and 53.5 (IQR, 44 to 56.25) in healthy individuals. Besides, tetracycline resistance genes and β-lactam resistance genes identified in patient guts were significantly higher compared with healthy individuals (Fig. 4C).

Antibiotic efflux pump mechanisms are ubiquitous among bacteria and greatly contribute to antimicrobial resistance, which is a major challenge for the development of new antibiotics. A total of 55 antibiotic efflux pump genes were detected in 64 metagenomic samples, and the efflux pump genes detected in human samples (*n* = 26) were fewer than in poultry (*n* = 53), swine (*n* = 38), and cow (*n* = 42) samples (Table S3 in Supplemental File 1). Tetracycline, chloramphenicol, macrolide-lincosamide-streptogramin B (MLSB), bacitracin, and multidrug efflux were identified using AmpliSeq method, among which the dominant efflux pump gene was the phenicols resistance gene *floR* (100%) followed by the tetracycline efflux gene *tetA* and multidrug efflux gene *tolC* (96.88%) (Table S3 in Supplemental File 1). High detection rates (>80%) were found in 11 efflux pump genes, suggesting the widespread antibiotic efflux pump genes in the environment.

**FIG 5:**
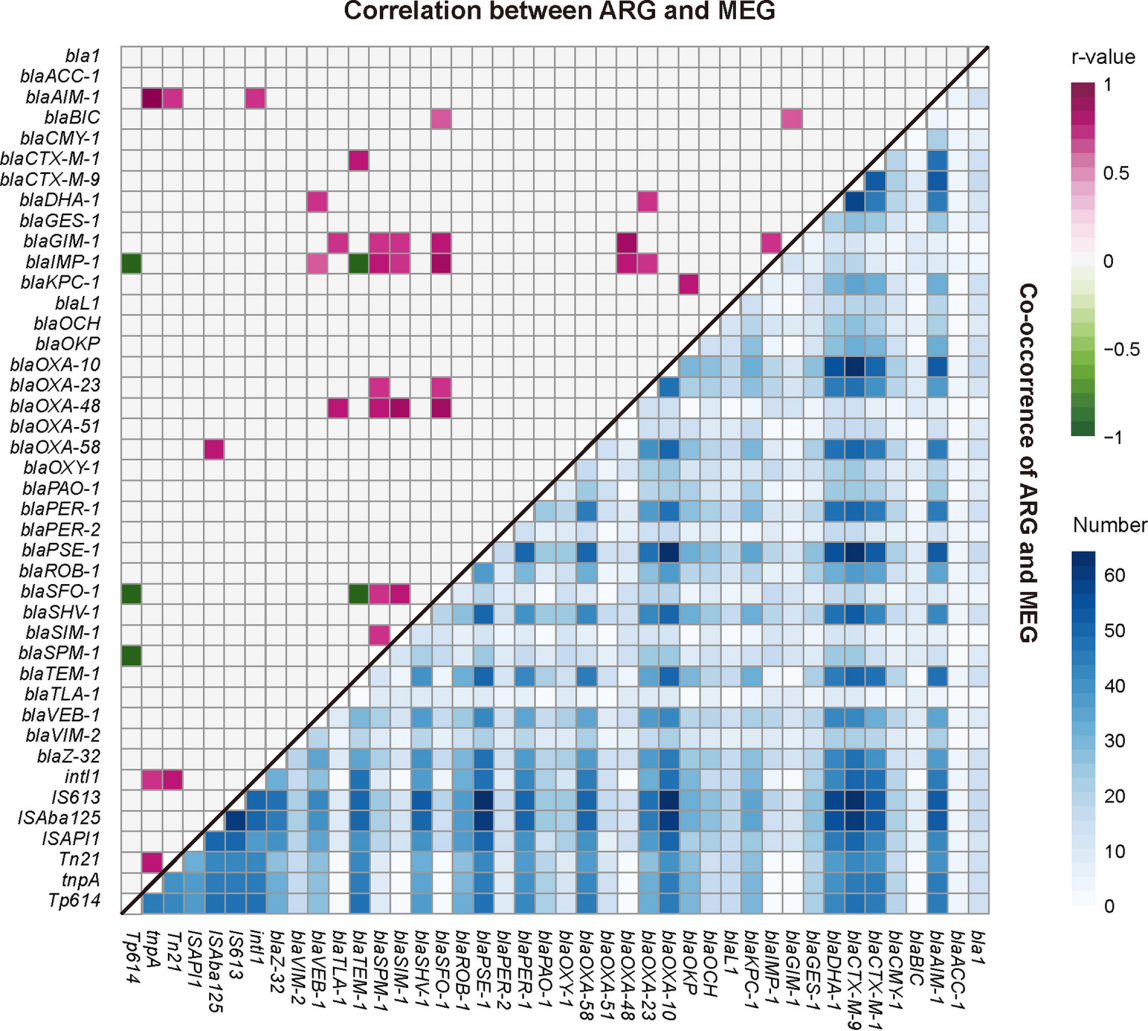
Correlation (upper triangle) and co-occurrence of ARGs and MGEs (lower triangle) (*n* = 64). r, correlation coefficient, which was calculated by gene abundance (RPKM). Only correlations that were found to be statistically significant (the absolute value of *r* > 0.7 and *P* < 0.01) were shown (upper triangle). The low triangle exhibited a pairwise co-occurrence matrix of all ARGs and MGEs detected. The colors denote the cases when two genes coexisted.

Furthermore, the relationship between ARGs and MGEs was also analyzed for 64 metagenomic samples. Fig. 5 illustrated several potential associations between β-lactam resistance genes and 7 MGEs, suggesting the cotransfer of ARGs and mobile genetic elements in metagenomic samples. Three MGEs, including *Tn*21, *int*I1, and *tnpA*, showed significant association to β-lactam resistance gene *bla*_AIM-1_, respectively, and to each other (Fig. 5). It is noteworthy that a strong correlation was observed between carbapenem resistance gene *bla*_OXA-48_ and insertional sequences *ISA*ba125 (*r* = 0.8, *P* < 0.01), which was thought to play important roles in the dissemination of new metallobeta-lactamase gene *bla*_NDM_ (30) (Fig. 5).

### Comparison of designed method in different platforms.

We further compared the performance of AmpliSeq (Thermo Fisher Scientific, USA) with ATOPlex technology (MGI, China) by sequencing AMR genes in 4 environmental samples using the amplicon sequencing method. First, high repeatability of the ATOPlex method was observed in a library in two trials (*R* = 0.984 for RPKM, *R* = 0.984 for UQ) (Fig. S4A and B in Supplemental File 1). Second, high mapping rates against the custom database (84 ARGs and 3 virulence factors) were found in both AmpliSeq and ATOPlex methods, which were 85.10% and 82.87%, respectively, indicating high specificity of the multiplex PCR targeted amplicon sequencing (Table S4 in Supplemental File 1).

**FIG 6:**
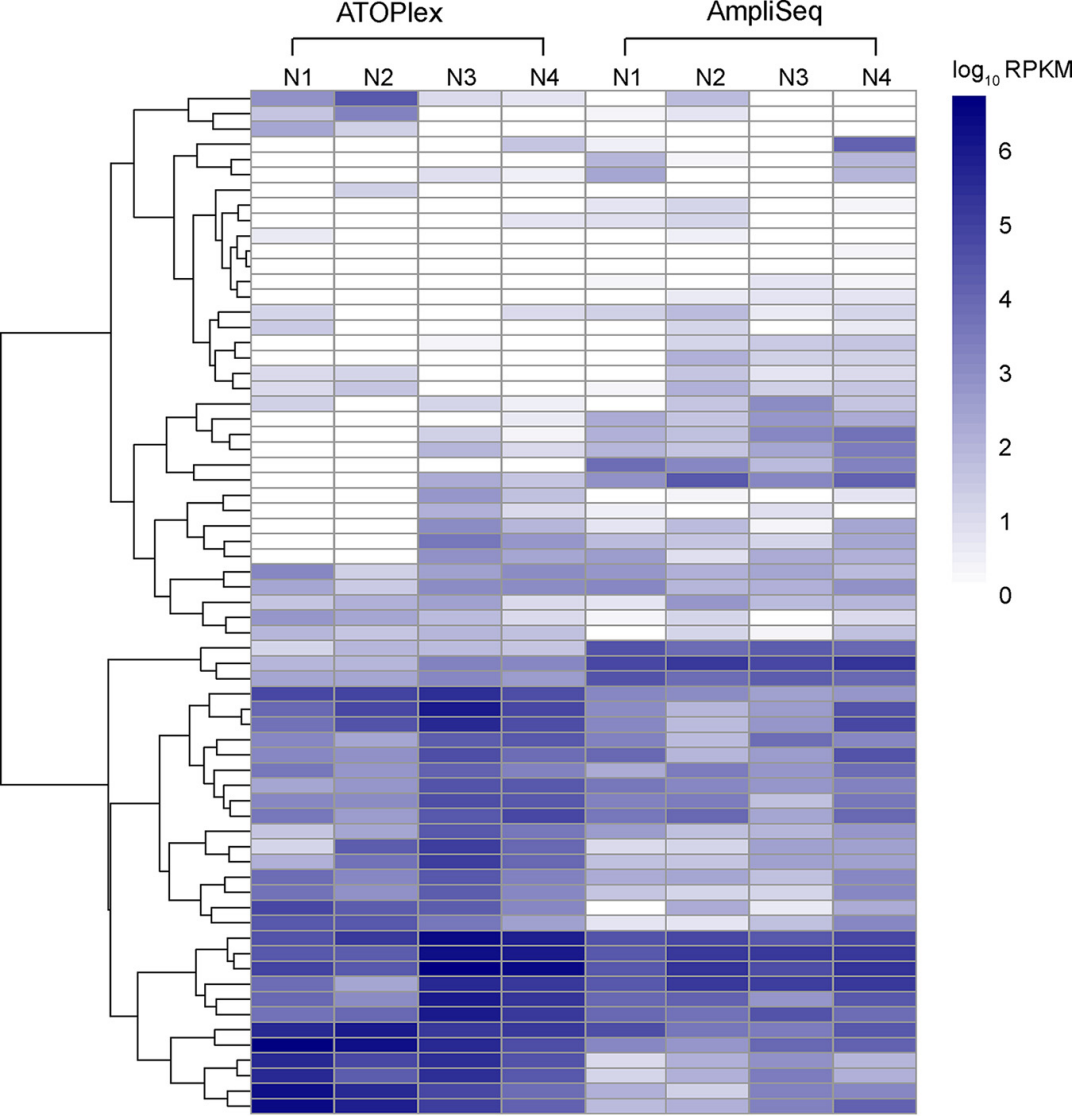
Application of designed approach using AmpliSeq and ATOPlex platforms. The colors denote the counts of reads mapping to each detected gene between MetaSeq and AmpliSeq. RPKM was used to normalize read counts and log-transformed to produce the heat map.

Most target genes could be detected by both the Amplicon sequencing approach and amplicon sequencing methods enabling high recovery of target genes (Fig. 6 and Fig. S4D in Supplemental File 1). Although only one amplicon was designed for each target gene with the ATOPlex platform, the high detection rates of target genes were also observed, indicating the high sensitivity of the amplicon sequencing method for AMR genes even with one primer pair. Gene diversity was slightly higher when using AmpliSeq against ATOPlex technology (Fig. S4C in Supplemental File 1). The differences between the two platforms may be due to the library construction differences from primer specificity and sample heterogeneity. The limited sample size can also be an influencing factor and further studies will be conducted in the future.

Three hypervirulence factors in Klebsiella pneumoniae, *kpiD* and *rmpA/rmpA2*, were also screened using the ATOPlex method, which is considered a dire threat to public health (31, 32). *kpiD* were identified in 13 of 208 environmental samples (6.25%), indicating the environment is a potential reservoir of virulence factors. However, these virulence genes present in very low abundance within each sample. The ability of the AmpliSeq method to amplify the signal of low-abundance target genes could provide a better assessment of the transmission of high-risk virulence genes in the environment.

## DISCUSSION

Recently, targeted sequencing has been widely applied for the detection of bacteria, viruses, and human diseases (33–35). Antimicrobial resistance has been considered a serious threat to global public health. Conventional metagenomic approaches have approved its vital role in understanding the resistome in a variety of environments. However, the complex variation and composition of the microbiome in the different environments could cause bias in the understanding of the resistome because ARGs were not evenly distributed in the bacteria host, and some ARGs more important to public health were usually underrepresented through the MetaSeq approach. To overcome this issue, this study describes the development of a novel multiplex PCR-based sequencing method for ARGs in metagenomes and its comparative evaluation with metagenomic shotgun sequencing. Our results suggest this method is promising in describing resistomes in complex environmental and clinical metagenomes and provides the possibility for further studies on the dissemination and diversity of antibiotic resistance, and even diagnosis in clinical settings.

Compared with metagenomic shotgun sequencing, several advantages were observed in the approach for studying resistomes in diverse environments. First, targeted amplification before sequencing enables better recovery of resistance genes, even low-abundance proportional resistomes in complex metagenomes, which greatly improved the sensitivity. Second, amplicon sequencing achieves higher horizontal alignment coverage of ARGs identified, suggesting higher specificity of this method. Third, our approach identified a greater diversity of antimicrobial resistance genes, which is a cost-effective and time-effective alternative to metagenomic shotgun sequencing for the analysis of resistomes.

Lately, multiplex PCR-based amplicon sequencing methods are increasingly used to describe AMR in complex samples. In a pilot study, the amplicon sequencing method of Neisseria gonorrhoeae AMR genes was established and evaluated. The method is based on Nanopore sequencing and can simultaneously sequence 13 genes within 11 h for clinical samples and 2 h for isolates (36). Amplicon sequencing was also tested on MinION platforms for describing 9 known drug resistance determinations and novel variations in tuberculosis directly from clinical samples. After calibrating the variant call, the method demonstrated 100% agreement between Illumina whole-genome sequencing (WGS) and MinION (37). A descriptive study investigated feces samples from clinically healthy infants and pregnant women using both MetaSeq and AmpliSeq methods (Ion Torrent platform). Results generated from the AmpliSeq method were consistent with ARGs detected by MetaSeq, and resistance to 2 additional antibiotic classes was further identified with the AmpliSeq method (38). To date, amplicon sequencing methods for AMR have been applied in multiple platforms and samples. However, most research only focuses on a small number of ARGs (36, 37), and amplicon sequencing methods for large-scale AMR screening are still lacking. In this study, we described a custom amplicon sequencing approach for large-scale detection and characterization of AMR determinations, including 251 clinically important ARGs, 8 MGEs, and 19 MRGs. Compared with the current commercially available community panel for ARGs (38), this highly customized approach has added several newly discovered important ARGs (such as tigecycline resistance genes *tet*X3 and *tet*X4) and significant increase the coverage of targeted genes, which can significantly elevate SNPs analysis. This approach has been successfully performed in 48 environmental (37 feces, 20 soils, and 7 sewage) and 16 clinical metagenomic samples, which only requires a small amount (1 ng) of input DNA and can simultaneously identify 278 genes with 24h for multiple metagenomic samples.

In addition, we also further compared the performance of MetaSeq and AmpliSeq in SNPs detection for ARGs. In the current study, we demonstrated that amplicon sequencing is an efficient tool for analyzing the presence and frequency of SNPs in metagenomic samples and performed better than MetaSeq for specifically targeted genes, presumably because of the fewer reads generated by the shallower sequencing depth in MetaSeq method. Multiple previous studies focused on the use of deep amplicon sequencing for SNP genotyping and mutation detection (39–41). Iawo et al. (39) reported that nested multiplex PCR with amplicon sequencing allows rapidly and accurately determination of Mycobacterium leprae drug resistance and SNP genotype directly from clinical samples, and three distinct mutations were identified in dapsone resistance gene *folP1*. Onda et al. (40) assessed the practical usability of the multiplex PCR targeted amplicon sequencing method in SNP genotyping. The assessment results indicated that multiplex PCR targeted amplicon sequencing enables accurately and stably perform genotyping and allows the easy design of custom target SNP-marker panels for various organisms. Guérillot et al. (41) presented resistance mutation sequencing, a new amplicon-based deep sequencing workflow, which will facilitate comprehensive detection, assessment, and surveillance of mutational resistance from bacterial populations. The environment acts as an important reservoir of ARGs, which are increasingly recognized as environmental contaminants. A customized panel can be used to assess the variation and evolution of specific ARGs in the environment through SNP analysis and provides a theoretical basis for rational utilization of antibiotics in clinical and aquaculture environments.

In this study, the application of this method was successfully applied to feces, soil, and sewage samples. This workflow could easily be extended to other sample types, such as saliva, blood, or other metagenomic samples. One limitation of our approach was that a finite number of antibiotic resistance genes were included. However, other interesting genes could be further introduced if necessary, and the flexible combination of the sequencing methods does have its advantage, considering the number of novel ARGs has been growing. Another limitation is that this approach is only being currently investigated at the laboratory, lacking systematic validation when applied to different situations (such as a clinical application). While the problem of sequencing contamination, which often arises in clinical samples, has been considered in this study. First, the laboratory environment was stringently cleaned and disinfected to eliminate nucleic acid contamination. Second, negative controls and technical replicates have been introduced to ensure accurate and stable target detection. Besides, all operations were carried out in separate regions to prevent cross-contamination throughout the process.

This study applied targeted amplification for the enrichment of AMR genes in metagenomes, aiming to achieve rapid and high-throughput detection of antibiotic resistance. We believe that amplicon sequencing will play a key role in the monitoring and surveillance of AMR in complex environmental and clinical samples. In the future, we could apply this method to serve bacterial species classification, antibiotic resistance, and virulence gene identification, which will be strong support for clinical diagnosis, antimicrobial drug selection, and antimicrobial-resistant bacteria surveillance.

## MATERIALS AND METHODS

### AmpliSeq panel design.

The reference database is homemade, which comprised both known ARGs and transfer elements for carrying ARGs and will be available upon request. The nucleotide acid sequences of ARGs determinants were downloaded from several ARGs database, including Resfinder, CARD, and AMRFinderPlus database, mobile genetic elements (MGEs) from the MGEfinder database, and metal resistance genes (MRGs) from the BacMet database (30, 42–44). All ARG nucleic acid sequences were pooled, a similarity search was conducted, and the redundant sequence was removed according to predetermined criteria (sequence identity >90%).

A custom database with 278 nucleic acid sequences was determined based on the antibiotics category. We selected genes that confer resistance to 11 different classes of antibiotics: aminoglycoside (*n* = 29), bacitracin (*n* = 3), β-lactams (*n* = 43), chloramphenicol (*n* = 11), fosfomycin (*n* = 5), macrolide-lincosamide-streptogramin B (MLSB, *n* = 43), nitroimidazoles (*n* = 1), polypeptide (*n* = 36), a quinolone (*n* = 11), sulfonamides (*n* = 8), tetracycline (*n* = 29). In addition, ARGs confer resistance to multidrug (*n* = 28), and several other antibiotics categories (*n* = 9) were also included. For each antibiotics category, genes were selected based on ARGs prevalence according to multiple previous studies and existing ARGs targeting methods (45–50). These sequences were then provided for primer design and screening using the Ion AmpliSeq strategy and the Ion AmpliSeq software (Thermo Fisher Scientific, USA) (27). Several rounds of primer optimization had gone through to come up with a set of primers with high specificity and efficiency. To prevent off-target hybridization of primers, BLAST analysis was used to compare the candidate primers with the human reference genome and GenBank’s nonredundant nucleotide database. Primers with high sequence similarity (>80%) were discarded. All primer pairs were combined to evaluate the presence of secondary structures, such as primer dimer. Besides, the control assay was conducted using positive templates to confirm the efficiency and feasibility of the final primer pools. The sequences for amplicons generated in this study are provided (Table S5 in Supplemental File 1).

### Sample preparation and DNA isolation.

Feces, soil, and wastewater samples from animal (swine, cow, and poultry) farms, and feces samples from humans (hospital) were collected which was approved by the ethics committee. Feces samples were collected immediately after defecation and soil samples were taken from the 0 to 10 cm depth, as previously described (51). All samples were kept frozen during transportation and stored in a −80°C freezer until DNA extraction. PowerSoil DNA isolation kit (Qiagen, Germany) was used to isolate metagenomic DNA from feces and soil samples. Qubit Fluorometer and Qubit dsDNA HS assay kit (Thermo Fisher Scientific, USA) were used to estimate the quantity and quality of extracted DNA. Genomic DNA is stored at −20°C until library preparation.

### Targeted amplification and library construction.

The full process of amplicon library preparation and sequencing is demonstrated in Fig. 1A. A custom AmpliSeq Panel designed in this study was used to amplify the AMR and MGE genes from the genomic DNA extracted from each sample. The 5× Ion AmpliSeq HiFi Mix (Thermo Fisher Scientific, USA) was used to perform 19 cycles of PCR amplification, with denaturing at 99°C for 2 min, denaturing at 99°C for 15 s, and annealing and extension at 60°C for 4 min. Amplicon libraries were prepared from the amplified DNA following standard commercial kit instructions for Ion AmpliSeq Library kit 2.0 (Thermo Fisher Scientific, USA). Barcoded adapters were used in the second PCR to group the samples. DNA ligase (Thermo Fisher Scientific, USA) was used to perform the second PCR, and KAPA Pure Beads (KAPA Biosystems) was used to purify the resistomes libraries.

### Amplicon sequencing.

The quality and quantity of constructed libraries were evaluated by the qPCR quantification method according to the instructions of the Ion Library TaqMan Quantitation kit (Thermo Fisher Scientific, USA), and all individual libraries were diluted to ~50 pM concentrations. All libraries were then pooled in equimolar concentration before sequencing. Ion S5 sequencing kit (Thermo Fisher Scientific, USA) on Ion 530 chip (Thermo Fisher Scientific, USA) was used for amplicon libraries sequencing. Sequencing was performed according to the manufacturer’s instructions, and all operations were conducted in separate regions to prevent cross-contamination throughout the process.

### Comparison of AmpliSeq using different sequencing platforms.

To further evaluate AMR amplicon sequencing method application on different platforms, a pool of 84 AMR genes for both AmpliSeq and ATOPlex panel (MGI, China), and 3 additional virulence factors (*rmpA*, *rmpA2*, and *kpiD*) from hypervirulence in Klebsiella pneumoniae were added to ATOPlex panel (MGI, China) for primer design. A primer set with high specificity and efficiency was generated after multiple primer optimization, as described above. Amplicons were prepared by ATOPlex customized Library Prep Set (MGI, China) following the manufacturer’s instructions, and DNA nanoballs were generated by rolling circle amplification, which greatly improved sequencing accuracy, using the MGIEasy Circularization kit (MGI, China) (52). The quality and quantity of libraries were checked using Agilent BioAnalyzer and Qubit Fluorometer (Thermo Fisher Scientific, USA), with the final concentration at ~10ng/μL. MGISEQ-200RS High-throughput Sequencing platform (MGI, China) was used for amplicon libraries sequencing.

### Bioinformatic analysis of MetaSeq and AmpliSeq data.

The raw sequencing data for MetaSeq and AmpliSeq was generated using Illumina CASAVA 1.8 pipeline and AmpliSeq pipeline (Thermo Fisher, USA), respectively (51). Quality control of all sequencing data was conducted as previous study (51). Format conversion of raw FASTQ files was performed using SeqKit v0.13.2 (53). The paired-end reads were merged using FLASH-1.2.11 (http://ccb.jhu.edu/software/FLASH/). We established a bioinformatics analysis pipeline for in-depth analysis of ARGs, MGEs, and MRGs from the metagenome sequencing data. First, sequence reads were mapped against our custom database sequence, ARGs (*n* = 251), MGEs (*n* = 7), and MRGs (*n* = 19), using pblat v2.1 software (54). The output PSL file was parsed for overall mapping statistics, including query name, reference name, mapping position, mapping length, block count, etc.

A customized Perl program was utilized to filter and count reads that mapped to target genes. Subsequently, the depth, breadth, and evenness of coverage were compared to assess the efficiency of the method to cover the target genes completely and uniformly. Samtools was used to analyze the depth in each region using the BAM files (genome mapping results) to evaluate sequencing quality (55). The average percent coverage per target gene with different depths was counted using a homemade Perl script. The presence and frequency of SNPs identified in each gene were compared to assess the ability of the AmpliSeq method in SNP analysis. To identify variants in AMR and MGE gene sequences, samtools mpileup was used to identify single nucleotide polymorphisms (SNPs) in mapping reads and vcftools was used to summarize and filter out low-quality data.

### Statistical analysis.

Mapping reads were analyzed by two types of global normalization: (i) total counts, as in RPKM (17), and (ii) upper quartile (UQ) of counts (56). All statistical analyses were performed using GraphPad Prism v8.00 (La Jolla, CA, USA) and R v4.0.3 (Vienna, Australia). The correlation between trials was analyzed using Pearson’s test and the correlation between ARGs and MGEs was evaluated by Spearman’s test in the R program. *P* < 0.01 was considered statistically significant.

### Data availability.

Genome sequencing data that support the findings of this study have been deposited in the NCBI database under BioProject accession number PRJNA787361. Extra data supporting the findings of this study are available from the corresponding author upon reasonable request. The complete code used to generate the analysis reported in the manuscript is publicly available at the GitHub repository, https://github.com/liyiming-cau/AmpliSeq.
