# Supplementary material for: Multiplexed Target Enrichment Enables Efficient and In-Depth Analysis of Antimicrobial Resistome in Metagenomes
Source: Microbiol Spectr. 2022 Oct 26;10(6):e02297-22. doi: 10.1128/spectrum.02297-22 (PMC9769626; doi:10.1128/spectrum.02297-22)
Supplement: Supplemental file 1 — Supplemental material. Download spectrum.02297-22-s0001.pdf, PDF file, 2.3 MB [file spectrum.02297-22-s0001.pdf]

SUPPLEMENTARY MATERIALS

Supplementary Figure S1

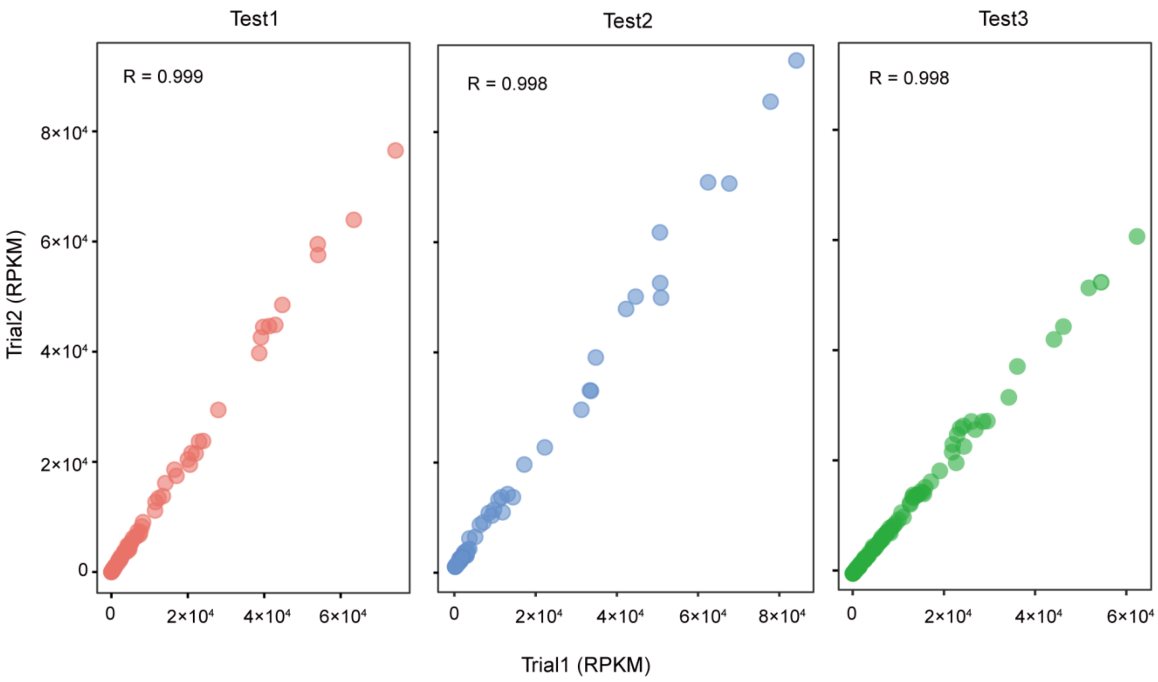

Figure S1. Consistency of amplicon sequencing method in different trials. Reads from targeted amplicons of three individual test samples in Tial 2 were subsampled to same depth as reads in Trail 1. Pearson correlation coefficients are shown in the diagram.

Supplementary Figure S2

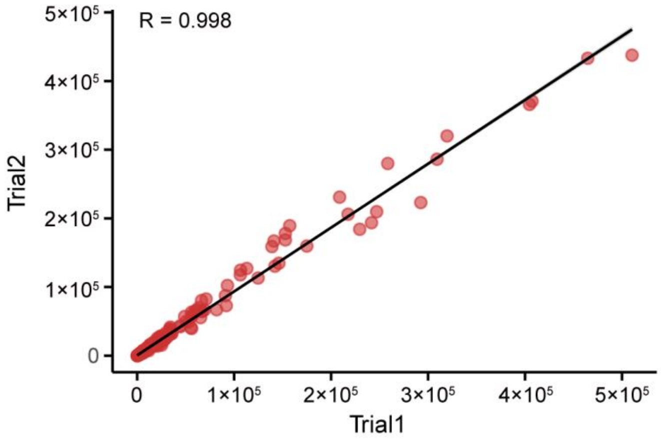

Figure S2. **Consistency of amplicon sequencing method in different trials using upper quartile (UQ) normalization.** Reads from targeted amplicons of three individual test samples in Trial 2 were subsampled to same depth as reads in Trail 1. Pearson correlation coefficients are shown in the diagram.

Supplementary Figure S3

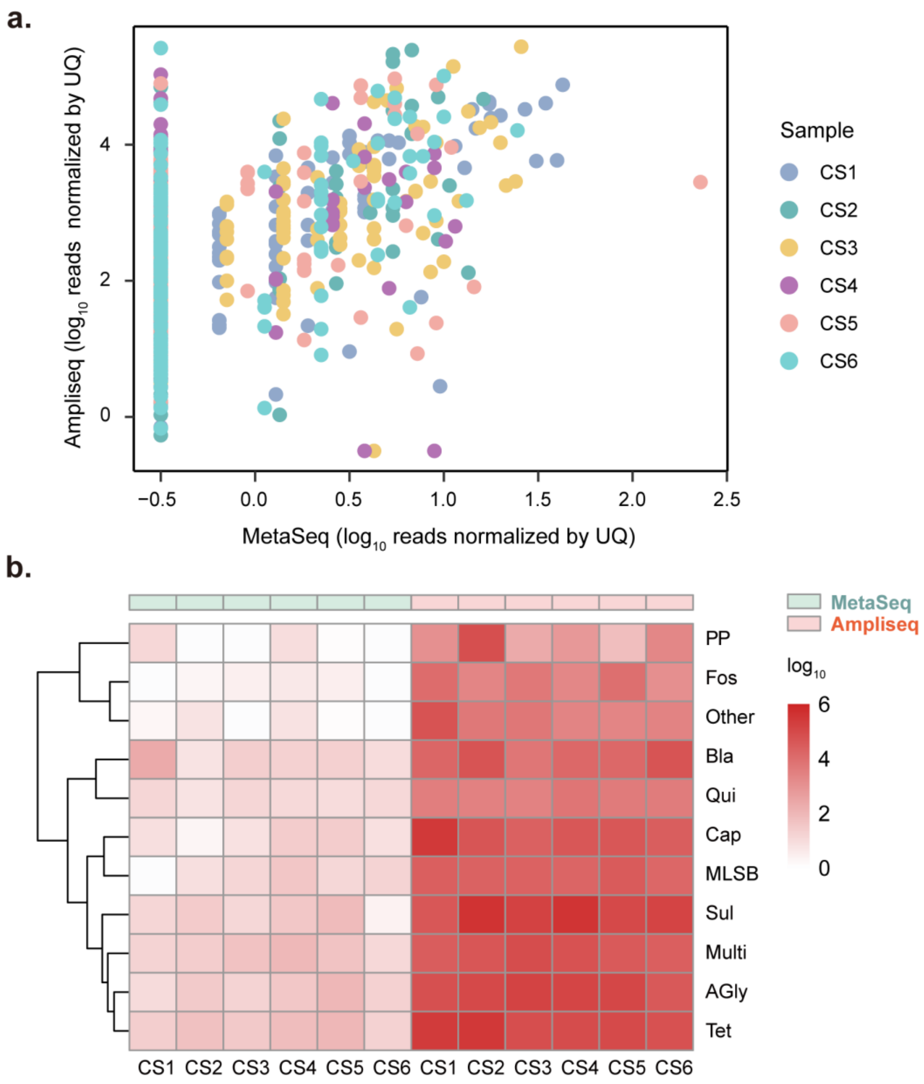

Figure S3. (a) Reads (normalized by upper quartile) mapping to the detected genes between MetaSeq and AmpliSeq. Genes only screened by AmpliSeq are showed in initial values of the abscissa axis. (b) Abundance of ARGs in AmpliSeq and MetaSeq. ARGs were classified into ten families (Sul: sulphonamides, AGly: aminoglycosides, Tet: tetracyclines, Bla: β-lactams, Cap: chloramphenicol, MLSB: macrolide-lincosamide-streptogramin B, Multi: multi-drug, PP: polypeptide, Fos: Fosfomycin, Qui: quinolone). Upper quartile (UQ) was used to normalized read counts and log-transformed to produce the heatmap.

Supplementary Figure S4

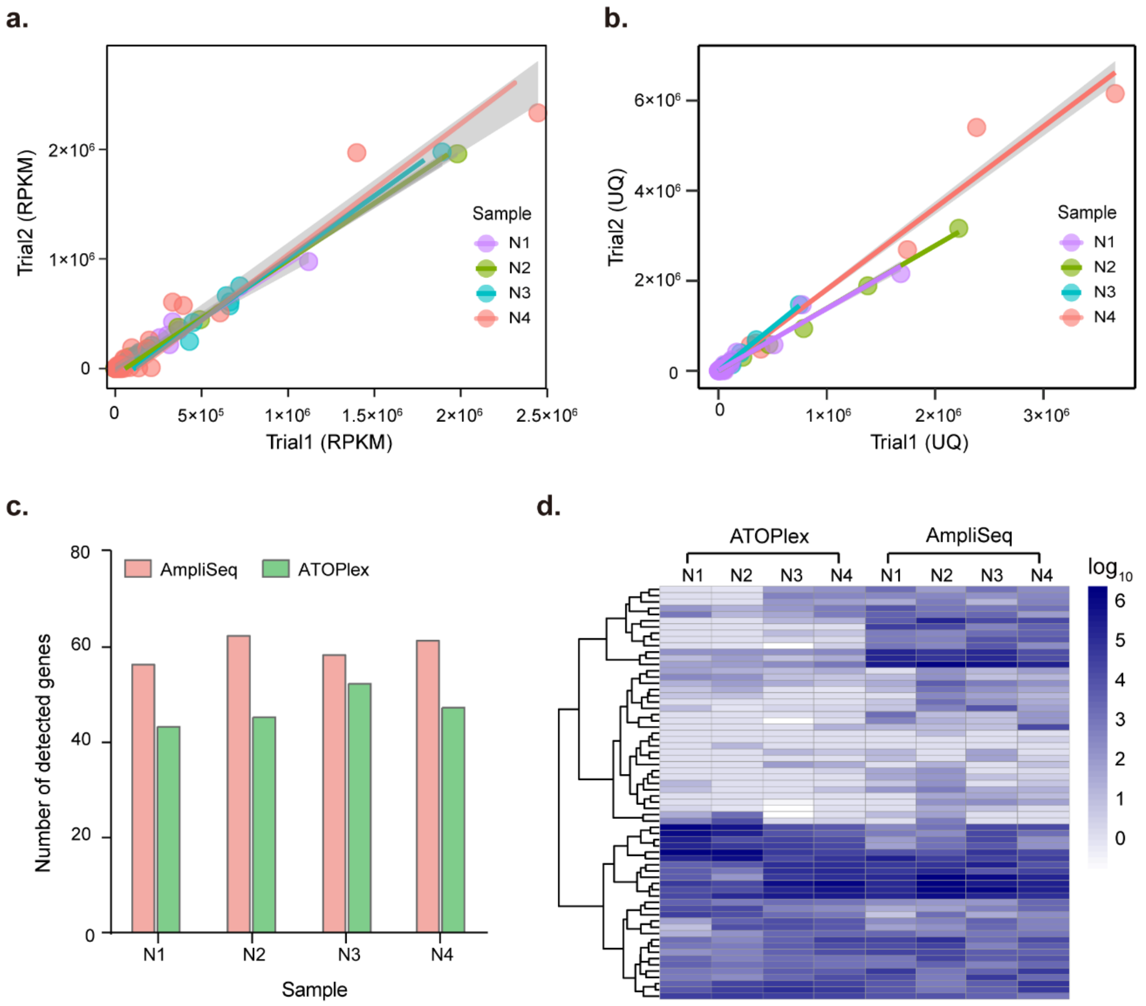

Figure S4. Application of designed method in ATOPlex platform. (a) Assessment of the consistency of ATOPlex method in different trials (normalized by UQ). (b) Assessment of the consistency of ATOPlex method in different trials (normalized by RPKM). (c) Comparison of AmpliSeq and ATOPlex methods in diversity of target genes. (d) Application of designed approach using AmpliSeq and ATOPlex platforms. UQ was used to normalized read counts and log-transformed to produce the heat map.

Table S1. Details of the 278 target genes included in the custom amplicon sequencing panel.

Gene	Classification	AMR Gene Family	Resistance Mechanism	Accession number
gyrA	Quinilone	fluoroquinolone resistant gyrA	antibiotic target alteration	MG995190
qepA	Quinilone	fluoroquinolone efflux MFS transporter QepA gene family	antibiotic efflux	AB263754
qnrA1	Quinilone	quinolone resistance protein (qnr)	antibiotic target protection	AY070235
qnrB1	Quinilone	quinolone resistance protein (qnr)	antibiotic target protection	EF682133
qnrB4	Quinilone	quinolone resistance protein (qnr)	antibiotic target protection	DQ303921
qnrB8	Quinilone	quinolone resistance protein (qnr)	antibiotic target protection	EU043312
qnrC	Quinilone	quinolone resistance protein (qnr)	antibiotic target protection	EU917444
qnrD	Quinilone	quinolone resistance protein (qnr)	antibiotic target protection	FJ228229
qnrS1	Quinilone	quinolone resistance protein (qnr)	antibiotic target protection	AB187515
qnrS2	Quinilone	quinolone resistance protein (qnr)	antibiotic target protection	JF261185
qnrVC1	Quinilone	quinolone resistance protein (qnr)	antibiotic target protection	EU436855
optrA	Chloramphenicol	ATP-binding cassette (ABC) antibiotic efflux pump	antibiotic efflux	NG_048023
catA1	Chloramphenicol	chloramphenicol acetyltransferase (CAT)	antibiotic inactivation	V00622
catB3	Chloramphenicol	chloramphenicol acetyltransferase (CAT)	antibiotic inactivation	AJ009818
catB8	Chloramphenicol	chloramphenicol acetyltransferase (CAT)	antibiotic inactivation	AF227506
cmlA1	Chloramphenicol	major facilitator superfamily (MFS) antibiotic efflux pump	antibiotic efflux	M64556
fexA	Chloramphenicol	major facilitator superfamily (MFS) antibiotic efflux pump	antibiotic efflux	AJ549214
floR	Chloramphenicol	major facilitator superfamily (MFS) antibiotic efflux pump	antibiotic efflux	AF071555
pexA	Chloramphenicol	major facilitator superfamily (MFS) antibiotic efflux pump	antibiotic efflux	NG_048035
fabK	Chloramphenicol	Others	antibiotic inactivation	FJ390165
cfr	Chloramphenicol	Cfr 23S ribosomal RNA methyltransferase	antibiotic target alteration	AM408573

cmx	Chloramphenicol	major facilitator superfamily (MFS) antibiotic efflux pump	antibiotic efflux	U85507
aac6-II	Aminoglycoside	AAC(6')	antibiotic inactivation	L12710
aac6-Ia	Aminoglycoside	AAC(6')	antibiotic inactivation	M18967
aac6-Ib	Aminoglycoside	AAC(6')	antibiotic inactivation	JN108884
aac6-Iy	Aminoglycoside	AAC(6')	antibiotic inactivation	AF144880
aacA_aphD	Aminoglycoside	APH(2''), AAC(6')	antibiotic inactivation	GU565967
aacC1	Aminoglycoside	AAC(3)	antibiotic inactivation	U04610
aacC2	Aminoglycoside	AAC(3)	antibiotic inactivation	AY138987
aac	Aminoglycoside	AAC(6')	antibiotic inactivation	AJ628983
aadA1	Aminoglycoside	ANT(3'')	antibiotic inactivation	KR610434
aadA2	Aminoglycoside	ANT(3'')	antibiotic inactivation	JQ364967
aadA5	Aminoglycoside	ANT(3'')	antibiotic inactivation	KT175895
aadA9	Aminoglycoside	ANT(3'')	antibiotic inactivation	AJ420072
aadA	Aminoglycoside	ANT(3'')	antibiotic inactivation	MH257908
aadD-1	Aminoglycoside	ANT(4')	antibiotic inactivation	AF181950
aadE	Aminoglycoside	ANT(6)	antibiotic inactivation	AB699882
aph2-Id	Aminoglycoside	APH(2'')	antibiotic inactivation	AF016483
aph6-Ia	Aminoglycoside	APH(6)	antibiotic inactivation	AY971801
aphA1	Aminoglycoside	APH(3')	antibiotic inactivation	HM146784
armA	Aminoglycoside	16S rRNA methyltransferase (G1405)	antibiotic target alteration	AY220558
rmtA	Aminoglycoside	16S rRNA methyltransferase (G1405)	antibiotic target alteration	AB120321
rmtB2	Aminoglycoside	16S rRNA methyltransferase (G1405)	antibiotic target alteration	JN968578
rmtC	Aminoglycoside	16S rRNA methyltransferase (G1405)	antibiotic target alteration	AB194779
rmtD	Aminoglycoside	16S rRNA methyltransferase (G1405)	antibiotic target alteration	DQ914960
rmtE	Aminoglycoside	16S rRNA methyltransferase (G1405)	antibiotic target alteration	GU201947
rmtG	Aminoglycoside	16S rRNA methyltransferase (G1405)	antibiotic target alteration	KJ004567

rmtH	Aminoglycoside	16S rRNA methyltransferase (G1405)	antibiotic target alteration	KC544262
rmtf	Aminoglycoside	16S rRNA methyltransferase (G1405)	antibiotic target alteration	JQ808129
spcN	Aminoglycoside	spectinomycin phosphotransferase	antibiotic inactivation	AF170704
strA	Aminoglycoside	APH(3'')	antibiotic inactivation	M96392
strB	Aminoglycoside	Aminoglycoside	antibiotic inactivation	EF493834
str	Aminoglycoside	Aminoglycoside	antibiotic inactivation	X92946
npmA	Aminoglycoside	16S rRNA methyltransferase (A1408)	antibiotic target alteration	AB261016
bla1	Beta_Lactamase	class A Bacillus anthracis Bla beta-lactamase	antibiotic inactivation	AF367983
blaACC-1	Beta_Lactamase	ACC beta-lactamase，classC	antibiotic inactivation	NG_048588
blaAIM-1	Beta_Lactamase	AIM beta-lactamase，classB	antibiotic inactivation	AM998375
blaBIC	Beta_Lactamase	BIC Beta-lactamase，classA	antibiotic inactivation	GQ260093
blaCMY	Beta_Lactamase	CMY beta-lactamase，classC	antibiotic inactivation	X92508
blaCTX-M-1	Beta_Lactamase	CTX-M beta-lactamase，classA	antibiotic inactivation	DQ915955
blaCTX-M-9	Beta_Lactamase	CTX-M beta-lactamase，classA	antibiotic inactivation	HM569735
blaDHA	Beta_Lactamase	DHA beta-lactamase，classC	antibiotic inactivation	Y16410
blaGES-1	Beta_Lactamase	GES beta-lactamase，classA	antibiotic inactivation	HQ170511
blaGIM-1	Beta_Lactamase	GIM beta-lactamase，classB	antibiotic inactivation	JF414726
blaIMP-1	Beta_Lactamase	IMP beta-lactamase，classA	antibiotic inactivation	DQ522237
blaKPC-1	Beta_Lactamase	KPC beta-lactamase，classB	antibiotic inactivation	AF297554
blaL1	Beta_Lactamase	L1 beta-lactamase	antibiotic inactivation	EF126059
blaNDM-5	Beta_Lactamase	NDM beta-lactamase，classB	antibiotic inactivation	NG_049337
blaOCH	Beta_Lactamase	OCH beta-lactamase，classC	antibiotic inactivation	LT840075
blaOKP	Beta_Lactamase	OKP beta-lactamase，classA	antibiotic inactivation	NG_049360
blaOXA-10	Beta_Lactamase	OXA beta-lactamase，classD	antibiotic inactivation	EU708817
blaOXA-23	Beta_Lactamase	OXA beta-lactamase，classD	antibiotic inactivation	FJ959346
blaOXA-48	Beta_Lactamase	OXA beta-lactamase，classD	antibiotic inactivation	AY236073

blaOXA-51	Beta_Lactamase	OXA beta-lactamase，classD	antibiotic inactivation	AJ309734
blaOXA-58	Beta_Lactamase	OXA beta-lactamase，classD	antibiotic inactivation	AY665723
blaOXY-1	Beta_Lactamase	OXY beta-lactamase，classA	antibiotic inactivation	Z30177
blaPAO-1	Beta_Lactamase	PDC beta-lactamase，classC	antibiotic inactivation	AY083595
blaPER-1	Beta_Lactamase	PER beta-lactamase，classA	antibiotic inactivation	GU944725
blaPER-2	Beta_Lactamase	PER beta-lactamase，classA	antibiotic inactivation	X93314
blaPSE-1	Beta_Lactamase	CARB beta-lactamase，classA	antibiotic inactivation	M69058
blaROB-1	Beta_Lactamase	ROB beta-lactamase，classA	antibiotic inactivation	DQ840517
blaSFO-1	Beta_Lactamase	FONA beta-lactamase，classA	antibiotic inactivation	AB003148
blaSHV-1	Beta_Lactamase	SHV beta-lactamase，classA	antibiotic inactivation	AF148850
blaSIM-1	Beta_Lactamase	SIM beta-lactamase，classB	antibiotic inactivation	JF731030
blaSPM-1	Beta_Lactamase	SPM beta-lactamase，classB	antibiotic inactivation	AY341249
blaTEM-1A	Beta_Lactamase	TEM beta-lactamase，classA	antibiotic inactivation	HM749966
blaTLA-1	Beta_Lactamase	TLA beta-lactamase，class A	antibiotic inactivation	AF148067
blaVEB-1	Beta_Lactamase	VEB beta-lactamase，classA	antibiotic inactivation	HM370393
blaVIM-2	Beta_Lactamase	VIM beta-lactamase，classB	antibiotic inactivation	KC907378
blaZ-32	Beta_Lactamase	blaZ beta-lactamase，classA	antibiotic inactivation	AP004832
cepA	Beta_Lactamase	cepA beta-lactamase	antibiotic inactivation	L13472
mecA	Beta_Lactamase	peptidoglycan synthesis	antibiotic target protection	NC_000913
carB-1	Beta_Lactamase	PSE family	antibiotic inactivation	HQ616211
pbp1a	Beta_Lactamase	penicillin-binding protein	antibiotic target protection	AF446215
pbp2a	Beta_Lactamase	penicillin-binding protein	antibiotic target protection	AF101780
pbp3	Beta_Lactamase	penicillin-binding protein	antibiotic target protection	AJ243120
pbp5	Beta_Lactamase	penicillin-binding protein	antibiotic target protection	AF375986
tet32	Tetracycline	tetracycline-resistant ribosomal protection protein	antibiotic target protection	EU722333
tet34	Tetracycline	tetracycline-resistant ribosomal protection protein	antibiotic target protection	AB061440

tet35	Tetracycline	tetracycline-resistant ribosomal protection protein	antibiotic target protection	AF353562
tet36	Tetracycline	tetracycline-resistant ribosomal protection protein	antibiotic target protection	AJ514254
tet37	Tetracycline	tetracycline-resistant ribosomal protection protein	antibiotic target protection	AF540889
tetAP	Tetracycline	major facilitator superfamily (MFS) antibiotic efflux pump	antibiotic efflux	AB054980
tetA	Tetracycline	major facilitator superfamily (MFS) antibiotic efflux pump	antibiotic efflux	X00006
tetBP	Tetracycline	tetracycline-resistant ribosomal protection protein	antibiotic target protection	NC_010937
tetB	Tetracycline	major facilitator superfamily (MFS) antibiotic efflux pump	antibiotic efflux	AP000342
tetC	Tetracycline	major facilitator superfamily (MFS) antibiotic efflux pump	antibiotic efflux	NC_003123
tetD	Tetracycline	major facilitator superfamily (MFS) antibiotic efflux pump	antibiotic efflux	AF467077
tetE	Tetracycline	major facilitator superfamily (MFS) antibiotic efflux pump	antibiotic efflux	L06940
tetG	Tetracycline	major facilitator superfamily (MFS) antibiotic efflux pump	antibiotic efflux	S52437
tetH	Tetracycline	major facilitator superfamily (MFS) antibiotic efflux pump	antibiotic efflux	Y16103
tetJ-1	Tetracycline	major facilitator superfamily (MFS) antibiotic efflux pump	antibiotic efflux	ACLE01000065
tetK	Tetracycline	major facilitator superfamily (MFS) antibiotic efflux pump	antibiotic efflux	U38428
tetL	Tetracycline	major facilitator superfamily (MFS) antibiotic efflux pump	antibiotic efflux	HM235948
tetM	Tetracycline	tetracycline-resistant ribosomal protection protein	antibiotic target protection	X92947
tetO	Tetracycline	tetracycline-resistant ribosomal protection protein	antibiotic target protection	M18896
tetQ-3	Tetracycline	tetracycline-resistant ribosomal protection protein	antibiotic target protection	U73497
tetR	Tetracycline	major facilitator superfamily (MFS) antibiotic efflux pump	antibiotic target alteration, antibiotic efflux	KF697108
tetS-3	Tetracycline	tetracycline-resistant ribosomal protection protein	antibiotic target protection	X92946
tetT	Tetracycline	tetracycline-resistant ribosomal protection protein	antibiotic target protection	L42544
tetU	Tetracycline	major facilitator superfamily (MFS) antibiotic efflux pump	antibiotic efflux	U01917
tetV	Tetracycline	major facilitator superfamily (MFS) antibiotic efflux pump	antibiotic efflux	AF030344
tetW	Tetracycline	tetracycline-resistant ribosomal protection protein	antibiotic target protection	DQ060146
tetX	Tetracycline	tetracycline inactivation enzyme	antibiotic inactivation	MT022428

tetX3	Tetracycline	tetracycline inactivation enzyme	antibiotic inactivation	MK134375
tetX4	Tetracycline	tetracycline inactivation enzyme	antibiotic inactivation	MK134376
vanA	Polypeptide	glycopeptide resistance gene cluster, van ligase	antibiotic target alteration	FJ866609
vanB	Polypeptide	glycopeptide resistance gene cluster, van ligase	antibiotic target alteration	AF192329
vanC	Polypeptide	glycopeptide resistance gene cluster, van ligase	antibiotic target alteration	EU151752
vanG-1	Polypeptide	glycopeptide resistance gene cluster, van ligase	antibiotic target alteration	AY271782
vanHB-1	Polypeptide	vanH, glycopeptide resistance gene cluster	antibiotic target alteration	AF192329
vanHD-1	Polypeptide	vanH, glycopeptide resistance gene cluster	antibiotic target alteration	AB242319
vanM	Polypeptide	glycopeptide resistance gene cluster, van ligase	antibiotic target alteration	AF288163
vanRA	Polypeptide	glycopeptide resistance gene cluster, vanR	antibiotic target alteration	FJ866609
vanRB	Polypeptide	glycopeptide resistance gene cluster, vanR	antibiotic target alteration	AF192329
vanRC-1	Polypeptide	glycopeptide resistance gene cluster, vanR	antibiotic target alteration	AF162694
vanRD-1	Polypeptide	glycopeptide resistance gene cluster, vanR	antibiotic target alteration	AB242319
vanSB-1	Polypeptide	vanS, glycopeptide resistance gene cluster	antibiotic target alteration	AF192329
vanTC	Polypeptide	glycopeptide resistance gene cluster, vanT	antibiotic target alteration	AF162694
vanTG-1	Polypeptide	glycopeptide resistance gene cluster, vanT	antibiotic target alteration	AY271782
vanWB	Polypeptide	vanW, glycopeptide resistance gene cluster	antibiotic target alteration	AF192329
vanWG	Polypeptide	vanW, glycopeptide resistance gene cluster	antibiotic target alteration	AY271782
vanXA-1	Polypeptide	vanX, glycopeptide resistance gene cluster	antibiotic target alteration	FJ866609
vanXB-1	Polypeptide	vanX, glycopeptide resistance gene cluster	antibiotic target alteration	AF192329
vanXD-1	Polypeptide	vanX, glycopeptide resistance gene cluster	antibiotic target alteration	AB242319
vanYB-1	Polypeptide	vanY, glycopeptide resistance gene cluster	antibiotic target alteration	AF192329
vanYD-1	Polypeptide	vanY, glycopeptide resistance gene cluster	antibiotic target alteration	AB242319
vanRC	Polypeptide	glycopeptide resistance gene cluster, vanR	antibiotic target alteration	EU151753
vanSA	Polypeptide	vanS, glycopeptide resistance gene cluster	antibiotic target alteration	M97297
vanSC	Polypeptide	vanS, glycopeptide resistance gene cluster	antibiotic target alteration	AF162694

vanSE	Polypeptide	vanS, glycopeptide resistance gene cluster	antibiotic target alteration	AY700375
vanTE	Polypeptide	glycopeptide resistance gene cluster, vanT	antibiotic target alteration	FJ872411
mcr-1	Polypeptide	MCR phosphoethanolamine transferase	antibiotic target alteration	KY853650
mcr-2	Polypeptide	MCR phosphoethanolamine transferase	antibiotic target alteration	LT598652
mcr-3-17	Polypeptide	MCR phosphoethanolamine transferase	antibiotic target alteration	MH332767
mcr-3-1	Polypeptide	MCR phosphoethanolamine transferase	antibiotic target alteration	KY924928
mcr-4	Polypeptide	MCR phosphoethanolamine transferase	antibiotic target alteration	MF543359
mcr-5	Polypeptide	MCR phosphoethanolamine transferase	antibiotic target alteration	KY807921
mcr-6	Polypeptide	MCR phosphoethanolamine transferase	antibiotic target alteration	MF176240
mcr-7	Polypeptide	MCR phosphoethanolamine transferase	antibiotic target alteration	MG267386
mcr-8	Polypeptide	MCR phosphoethanolamine transferase	antibiotic target alteration	MG736312
mcr-9	Polypeptide	MCR phosphoethanolamine transferase	antibiotic target alteration	MK791138
dfrA1	Sulfonamides	trimethoprim resistant dihydrofolate reductase dfr	antibiotic target replacement	MH574896
dfrA	Sulfonamides	trimethoprim resistant dihydrofolate reductase dfr	antibiotic target replacement	GU565967
dfrK	Sulfonamides	trimethoprim resistant dihydrofolate reductase dfr	antibiotic target replacement	FN390947
drfA12	Sulfonamides	trimethoprim resistant dihydrofolate reductase dfr	antibiotic target replacement	JN108892
folA	Sulfonamides	ATP-binding cassette (ABC) antibiotic efflux pump	antibiotic efflux	EU871432
sul1	Sulfonamides	sulfonamide resistant sul	antibiotic target replacement	EF667294
sul2	Sulfonamides	sulfonamide resistant sul	antibiotic target replacement	DQ464881
sulA	Sulfonamides	sulfonamide resistant sul	antibiotic inactivation	KJ831060
fosA3	Fosfomycin	glutathione S-transferase	antibiotic inactivation	KX245440
fosA	Fosfomycin	glutathione S-transferase	antibiotic inactivation	M85195
fosB	Fosfomycin	fosfomycin thiol transferase	antibiotic inactivation	X89875
fosXCC	Fosfomycin	fosfomycin thiol transferase	antibiotic inactivation	KC876749
fosX	Fosfomycin	fosfomycin thiol transferase	antibiotic inactivation	NG_047899
vatA	MLSB	streptogramin vat acetyltransferase	antibiotic inactivation	L07778

vatB	MLSB	streptogramin vat acetyltransferase	antibiotic inactivation	U19459
vatC	MLSB	streptogramin vat acetyltransferase	antibiotic inactivation	AF015628
vatE	MLSB	streptogramin vat acetyltransferase	antibiotic inactivation	AF242872
vgaA	MLSB	ATP-binding cassette (ABC) antibiotic efflux pump	antibiotic efflux	M90056
vgaA-V	MLSB	ATP-binding cassette (ABC) antibiotic efflux pump	antibiotic efflux	AF186237
vgaB	MLSB	ATP-binding cassette (ABC) antibiotic efflux pump	antibiotic efflux	U82085
vgaC	MLSB	ATP-binding cassette (ABC) antibiotic efflux pump	antibiotic efflux	NG_048559
vgaE	MLSB	ATP-binding cassette (ABC) antibiotic efflux pump	antibiotic efflux	FR772051
vgbB	MLSB	streptogramin vgb lyase	antibiotic inactivation	AF015628
lnuA	MLSB	lincosamide nucleotidyltransferase (LNU)	antibiotic inactivation	M14039
lnuB	MLSB	lincosamide nucleotidyltransferase (LNU)	antibiotic inactivation	AJ238249
lnuC	MLSB	lincosamide nucleotidyltransferase (LNU)	antibiotic inactivation	AY928180
lmrA	MLSB	lmrAB opero	antibiotic target protection	X59926
lsaB	MLSB	ATP-binding cassette (ABC) antibiotic efflux pump	antibiotic efflux	NG_047933
lsaE	MLSB	ATP-binding cassette (ABC) antibiotic efflux pump	antibiotic efflux	JX560992
speA	MLSB	other/efflux	antibiotic efflux	KY594413
ereA-2	MLSB	macrolide esterase	antibiotic inactivation	AF099140
ereB	MLSB	macrolide esterase	antibiotic inactivation	X03988
mefB	MLSB	major facilitator superfamily (MFS) antibiotic efflux pump	antibiotic efflux	FJ196385
mphA	MLSB	macrolide phosphotransferase (MPH)	antibiotic inactivation	D16251
mphB	MLSB	macrolide phosphotransferase (MPH)	antibiotic inactivation	D85892
mphC	MLSB	macrolide phosphotransferase (MPH)	antibiotic inactivation	AB013298
mefA	MLSB	major facilitator superfamily (MFS) antibiotic efflux pump	antibiotic efflux	AF227521
msrD	MLSB	ATP-binding cassette (ABC) antibiotic efflux pump	antibiotic efflux	AF274302
msrA	MLSB	ATP-binding cassette (ABC) antibiotic efflux pump	antibiotic efflux	X52085
msrC	MLSB	ATP-binding cassette (ABC) antibiotic efflux pump	antibiotic efflux	AY004350

erm34	MLSB	Erm 23S ribosomal RNA methyltransferase	antibiotic target alteration	AY234334
erm35	MLSB	Erm 23S ribosomal RNA methyltransferase	antibiotic target alteration	AF319779
erm36	MLSB	Erm 23S ribosomal RNA methyltransferase	antibiotic target alteration	AF462611
ermA	MLSB	Erm 23S ribosomal RNA methyltransferase	antibiotic target alteration	X03216
ermB-18	MLSB	Erm 23S ribosomal RNA methyltransferase	antibiotic target alteration	X66468
ermC	MLSB	Erm 23S ribosomal RNA methyltransferase	antibiotic target alteration	V01278
ermD	MLSB	Erm 23S ribosomal RNA methyltransferase	antibiotic target alteration	M29832
ermE	MLSB	Erm 23S ribosomal RNA methyltransferase	antibiotic target alteration	X51891
ermF	MLSB	Erm 23S ribosomal RNA methyltransferase	antibiotic target alteration	M17124
ermG	MLSB	Erm 23S ribosomal RNA methyltransferase	antibiotic target alteration	M15332
ermK	MLSB	Erm 23S ribosomal RNA methyltransferase	antibiotic target alteration	M77505
ermT	MLSB	Erm 23S ribosomal RNA methyltransferase	antibiotic target alteration	M64090
ermX	MLSB	Erm 23S ribosomal RNA methyltransferase	antibiotic target alteration	M36726
ermY	MLSB	Erm 23S ribosomal RNA methyltransferase	antibiotic target alteration	AB014481
mel	MLSB	ATP-binding cassette (ABC) antibiotic efflux pump	antibiotic efflux	KU984333
oleC	MLSB	ATP-binding cassette (ABC) antibiotic efflux pump	antibiotic efflux	L06249
oqxA	Multidrug	resistance-nodulation-cell division (RND) antibiotic efflux pump	antibiotic efflux	EU370913
oqxB	Multidrug	resistance-nodulation-cell division (RND) antibiotic efflux pump	antibiotic efflux	EU370913
cmeA	Multidrug	resistance-nodulation-cell division (RND) antibiotic efflux pump	antibiotic efflux	AB894099
cmeB	Multidrug	resistance-nodulation-cell division (RND) antibiotic efflux pump	antibiotic efflux	AB894099
cmeC	Multidrug	resistance-nodulation-cell division (RND) antibiotic efflux pump	antibiotic efflux	AB894099
RE-cmeA	Multidrug	resistance-nodulation-cell division (RND) antibiotic efflux pump	antibiotic efflux	KT778507
RE-cmeB	Multidrug	resistance-nodulation-cell division (RND) antibiotic efflux pump	antibiotic efflux	KT778507
RE-cmeC	Multidrug	resistance-nodulation-cell division (RND) antibiotic efflux pump	antibiotic efflux	KT778507
tolC	Multidrug	resistance-nodulation-cell division (RND) antibiotic efflux pump	antibiotic efflux	FJ768952
mepA	Multidrug	multidrug and toxic compound extrusion (MATE) transporter	antibiotic efflux	AY661734

ceoA	Multidrug	resistance-nodulation-cell division (RND) antibiotic efflux pump	antibiotic efflux	U97042
adeA	Multidrug	resistance-nodulation-cell division (RND) antibiotic efflux pump	antibiotic efflux	KX154813
cmr	Multidrug	major facilitator superfamily (MFS) antibiotic efflux pump	antibiotic efflux	U44900
mdetl1	Multidrug	major facilitator superfamily (MFS) antibiotic efflux pump	antibiotic inactivation	AJ012115
mdtE	Multidrug	resistance-nodulation-cell division (RND) antibiotic efflux pump	antibiotic inactivation	NC_000913
pikR1	Multidrug	erm_methylase	antibiotic inactivation	AF079138
pikR2	Multidrug	erm_methyltransferase	antibiotic inactivation	AF079138
mtrC	Multidrug	resistance-nodulation-cell division (RND) antibiotic efflux pump	antibiotic efflux	HQ712081
mtrD	Multidrug	resistance-nodulation-cell division (RND) antibiotic efflux pump	antibiotic efflux	HQ706341
qacH	Multidrug	small multidrug resistanc (SMR) efflux pump	antibiotic efflux	AY816216
qac	Multidrug	small multidrug resistanc (SMR) efflux pump	antibiotic efflux	U81980
rarD	Multidrug	major facilitator superfamily (MFS) antibiotic efflux pump	antibiotic efflux	L02122
sdeB	Multidrug	resistance-nodulation-cell division (RND) antibiotic efflux pump	antibiotic efflux	CP033162
ttgA	Multidrug	resistance-nodulation-cell division (RND) antibiotic efflux pump	antibiotic efflux	AF031417
ttgB	Multidrug	resistance-nodulation-cell division (RND) antibiotic efflux pump	antibiotic efflux	AF031417
qacA	Multidrug	small multidrug resistanc (SMR) efflux pump	antibiotic efflux	NG_061385
qacE	Multidrug	small multidrug resistanc (SMR) efflux pump	antibiotic efflux	FJ663011
nisB	Multidrug	resistance-nodulation-cell division (RND) antibiotic efflux pump	antibiotic efflux	X68307
bacA	Bacitracin	undecaprenol kinase	antibiotic inactivation	AF169967
bcrB	Bacitracin	ATP-binding cassette (ABC) efflux pump	antibiotic efflux	KX772240
bcrR	Bacitracin	ATP-binding cassette (ABC) efflux pump	antibiotic efflux	KX772240
nimE	Nitroimidazoles	pyridoxamine 5'-phosphate oxidase protein	antibiotic inactivation	NG_048015
pncA	Antituberculosis	Other/efflux	antibiotic inactivation	KY659393
sat	Others	Transposase	transposase	AB699882
IS613	Others	Transposase	transposase	MH025900
ISAPI1	Others	Transposase	transposase	MF175187

ISAba125	Others	Transposase	transposase	NC_022740
Tn21	Others	Transposase	transposase	KY913897
Tp614	Others	Transposase	transposase	DQ831146
tnpA	Others	Transposase	transposase	JX570731
intI1	Others	integron integrase	integron	NZ_CP039294
merA	Hg	mercury(II) reductase	metal ions reduction	AB024961
merB	Hg	Organomercurial lyase	organometallics reduction	HQ144191
pbrD	Pb	Lead binding protein	metal ions binding	X71400
cadB	Cd	Cadmium binding protein	metal ions binding	M67452
cadD	Cd	Cadmium binding protein	metal ions binding	AF134905
copA	Co	P-type ATPases	metal accumulation	AF187849
copB	Co	P-type ATPases	metal accumulation	AF187849
cueO	Co	Copper detoxification	metal ions oxidation	NC_000913
cusF	Co	Copper accumulation	metal binding	KC146966
chrA	Cr	Chromate reductase	metal ions binding	NC_000964
NiCotT	Ni	Nickel cobalt transferase	metal accumulation	FUGF01000049
yfeA	Zn	periplasmic-binding protein; ABC transporter system	metal transport	U50597
zntA	Zn	Zn(II)-export protein	metal transport	NC_000913
zntB	Zn	Zn(II)-export protein	metal transport	NC_000913
ceuE	Fe	siderophore transport protein	metal transport	FN555004
czcA	Co/Zn/Cd	resistance-nodulation-cell division (RND) antibiotic efflux pump	metal transport	D67044
czcC	Co/Zn/Cd	outer membrane protein	target protection	D67044
arsA	As	ATPase arsA	metal transport	KY271405
arsB	As	a membrane pump that functions alone or with ATPase arsA	metal transport	AB004659

Table S2. 92 unique genes identified by AmpliSeq compared with MetaSeq.

Classification	Resistance gene	Mechanism	Accession number
Quinilone	*qnrA1*	antibiotic target protection	AY070235
	qnrC	antibiotic target protection	EU917444
Aminoglycoside	*aac6-II*	antibiotic inactivation	L12710
	aac6-Iy	antibiotic inactivation	AF144880
	aadA	antibiotic inactivation	MH257908
	aadD-1	antibiotic inactivation	AF181950
	aph2-Id	antibiotic inactivation	AF016483
	armA	antibiotic target alteration	AY220558
	rmtf	antibiotic target alteration	JQ808129
	spcN	antibiotic inactivation	AF170704
	strB	antibiotic inactivation	EF493834
	npmA	antibiotic target alteration	AB261016
β-Lactamase	*bla1*	antibiotic inactivation	AF367983
	bla_{CMY}	antibiotic inactivation	X92508
	bla_{DHA}	antibiotic inactivation	Y16410
	bla_{GES-1}	antibiotic inactivation	HQ170511
	bla_{KPC-1}	antibiotic inactivation	AF297554
	bla_{OCH}	antibiotic inactivation	LT840075
	bla_{OKP}	antibiotic inactivation	NG_049360
	bla_{OXA-23}	antibiotic inactivation	FJ959346

	bla$_{OXA-51}$	antibiotic inactivation	AJ309734
	bla$_{OXA-58}$	antibiotic inactivation	AY665723
	bla$_{OXY-1}$	antibiotic inactivation	Z30177
	bla$_{PAO-1}$	antibiotic inactivation	AY083595
	bla$_{PER-1}$	antibiotic inactivation	GU944725
	bla$_{PER-2}$	antibiotic inactivation	X93314
	bla$_{PSE-1}$	antibiotic inactivation	M69058
	bla$_{ROB-1}$	antibiotic inactivation	DQ840517
	bla$_{SHV-1}$	antibiotic inactivation	AF148850
	bla$_{SPM-1}$	antibiotic inactivation	AY341249
	bla$_{VIM-2}$	antibiotic inactivation	KC907378
	cepA	antibiotic inactivation	L13472
	pbp2a	antibiotic target protection	AF101780
	pbp5	antibiotic target protection	AF375986
	tet32	antibiotic target protection	EU722333
Tetracycline	*tetB*	antibiotic efflux	AP000342
	tetD	antibiotic efflux	AF467077
	tetE	antibiotic efflux	L06940
	tetH	antibiotic efflux	Y16103
	tetJ-1	antibiotic efflux	ACLE01000065
	tetS-3	antibiotic target protection	X92946
	tetU	antibiotic efflux	U01917
Polypeptide	*vanA*	antibiotic target alteration	FJ866609

	vanB	antibiotic target alteration	AF192329
	vanC	antibiotic target alteration	EU151752
	vanHB-1	antibiotic target alteration	AF192329
	vanRA	antibiotic target alteration	FJ866609
	vanRB	antibiotic target alteration	AF192329
	vanSB-1	antibiotic target alteration	AF192329
	vanTC	antibiotic target alteration	AF162694
	vanTG-1	antibiotic target alteration	AY271782
	vanXA-1	antibiotic target alteration	FJ866609
	vanRC	antibiotic target alteration	EU151753
	vanSA	antibiotic target alteration	M97297
	vanSC	antibiotic target alteration	AF162694
	mcr-2	antibiotic target alteration	LT598652
	mcr-4	antibiotic target alteration	MF543359
	mcr-5	antibiotic target alteration	KY807921
	mcr-6	antibiotic target alteration	MF176240
	mcr-7	antibiotic target alteration	MG267386
	mcr-8	antibiotic target alteration	MG736312
Sulfonamides	*dfrA1*	antibiotic target replacement	MH574896
MLSB	*vatA*	antibiotic inactivation	L07778
	vatC	antibiotic inactivation	AF015628
	vgaA	antibiotic efflux	M90056
	vgaA-V	antibiotic efflux	AF186237
	vgaC	antibiotic efflux	NG_048559

	vgaE	antibiotic efflux	FR772051
	vgbB	antibiotic inactivation	AF015628
	lnuC	antibiotic inactivation	AY928180
	lmrA	antibiotic target protection	X59926
	lsaB	antibiotic efflux	NG_047933
	ereA-2	antibiotic inactivation	AF099140
	ereB	antibiotic inactivation	X03988
	mphB	antibiotic inactivation	D85892
	msrA	antibiotic efflux	X52085
	msrC	antibiotic efflux	AY004350
	erm34	antibiotic target alteration	AY234334
	erm35	antibiotic target alteration	AF319779
	ermD	antibiotic target alteration	M29832
	ermK	antibiotic target alteration	M77505
	cmeB	antibiotic efflux	AB894099
	RE-cmeA	antibiotic efflux	KT778507
	RE-cmeB	antibiotic efflux	KT778507
Multidrug	*RE-cmeC*	antibiotic efflux	KT778507
	adeA	antibiotic efflux	KX154813
	mdetl1	antibiotic inactivation	AJ012115
	pikR1	antibiotic inactivation	AF079138
	pikR2	antibiotic inactivation	AF079138

	qac	antibiotic efflux	U81980
	qacA	antibiotic efflux	NG_061385
	nisB	antibiotic efflux	X68307

Table S3. Identification of antibiotic efflux pump genes in poultry, swine, cow, and human samples.

Classification	Resistance gene	Mechanism	Poultry (20 samples)	Swine (16 samples)	Cow (12 samples)	Human (16 samples)	Total
Tetracycline	*tetA*		100%	93.75%	100%	93.75%	**96.88%**
	tetAP		70.00%	93.75%	100%	50.00%	76.56%
	tetB		100%	75.00%	83.33%	43.75%	76.56%
	tetBP		85.00%	93.75%	100%	75.00%	87.50%
	tetD		45.00%	—	41.67%	18.75%	26.56%
	tetE		30.00%	6.25%	16.67%	—	14.06%
	tetG		100%	81.25%	100%	6.25%	71.88%
	tetH		85.00%	75.00%	66.67%	—	57.81%
	tetK		85.00%	18.75%	75.00%	—	45.31%
	tetL		100%	75.00%	100%	—	68.75%
	tetU		—	—	—	6.25%	1.56%
	tetV		10.00%	—	25.00%	—	7.81%
Chloramphenicol	*optrA*	ABC[b]	100%	43.75%	100%	—	60.94%
	cmlA1	MFS[c]	100%	93.75%	100%	87.50%	95.31%
	cmx		100%	93.75%	100%	6.25%	75.00%

	fexA		100%	62.50%	100%	12.50%	68.75%
	floR		100%	100%	100%	100%	**100%**
	lsaB		50.00%	18.75%	58.33%	—	31.25%
	lsaE		95.00%	100%	100%	18.75%	78.13%
	mel		100%	93.75%	91.67%	6.25%	73.44%
	msrA		50.00%	6.25%	41.67%	—	25.00%
	msrC	ABC[b]	35.00%	18.75%	—	31.25%	23.44%
	msrD		90.00%	93.75%	100%	93.75%	93.75%
MLSB[a]	*vgaA*		15.00%	—	—	—	4.69%
	vgaAV		20.00%	—	8.33%	—	7.81%
	vgaC		75.00%	—	8.33%	—	25.00%
	vgaE		90.00%	6.25%	66.67%	—	42.19%
	mefA		95.00%	93.75%	100%	93.75%	95.31%
	mefB	MFS[c]	85.00%	50.00%	100%	18.75%	62.50%
	oleC		30.00%	18.75%	50.00%	—	23.44%
Bacitracin	*bcrB*	ABC[b]	100%	12.50%	100%	75.00%	71.88%
	bcrR		90.00%	12.50%	100%	—	50.00%
	cmr	MFS[c]	95.00%	93.75%	91.67%	93.75%	93.75%
	rarD		90.00%	87.50%	91.67%	93.75%	90.63%
Multidrug	*adeA*		5.00%	—	—	—	1.56%
	ceoA	RND[d]	15.00%	—	—	—	4.69%
	cmeA		15.00%	—	—	—	4.69%
	cmeB		20.00%	—	—	—	6.25%

cmeC		10.00%	—	—	—	3.13%
mdtE		100%	93.75%	91.67%	87.50%	93.75%
mtrD		—	—	16.67%	—	3.13%
nisB		5.00%	6.25%	—	—	3.13%
oqxA		95.00%	56.25%	66.67%	93.75%	79.69%
oqxB		100%	87.50%	91.67%	93.75%	93.75%
RE-cmeA		20.00%	—	—	—	4.69%
RE-cmeB		20.00%	—	—	—	6.25%
RE-cmeC		20.00%	—	—	—	6.25%
sdeB		60.00%	31.25%	50.00%	37.50%	45.31%
tolC		100%	93.75%	91.67%	100%	**96.88%**
ttgA		25.00%	6.25%	41.67%	—	17.19%
ttgB		35.00%	18.75%	58.33%	—	26.56%
qac	SMR[e]	5.00%	—	—	—	1.56%
qacA		5.00%	—	8.33%	—	3.13%
qacE		10.00%	31.25%	25.00%	—	17.19%
qacH		100%	56.25%	91.67%	12.50%	65.63%

a: MLSB: Macrolide-lincosamide-streptogramin B; b: ABC: ATP-binding cassette antibiotic efflux pump; c: MFS: major facilitator superfamily antibiotic efflux pump; d: RND: resistance-nodulation-cell division antibiotic efflux pump; e: SMR: major facilitator superfamily antibiotic efflux pump.

Table S4. Comparison of amplicon sequencing in AmpliSeq and ATOPlex technology

Sample	AmpliSeq			ATOPlex			Gain[a]
	No. of raw reads	No. of aligned reads	Percent (%)	No. of raw reads	No. of aligned reads	Percent (%)	
N1	441,462	332,059	75.22	10,566,235	9,304,330	88.06	1.17
N2	887,932	781,502	88.01	4,963,350	4,352,514	87.69	1.00
N3	662,783	517,486	78.08	18,731,004	14,626,298	78.09	1.00
N4	1,085,548	988,067	91.02	5,295,532	4,497,448	84.93	0.93
Average	769,431	654,779	85.10	9,889,030	8195147.5	82.87	0.97

a: The results of dividing on-target percentage of AmpliSeq by ATOPlex.

Table S5. The information of amplicon sequences

Target gene	Start	End		Pool
IS613_MH025900	32	215	AMPL3931677	Pool=1
IS613_MH025900	204	429	AMPL3931678	Pool=2
IS613_MH025900	418	638	AMPL3931679	Pool=1
IS613_MH025900	627	846	AMPL3931680	Pool=2
IS613_MH025900	835	1057	AMPL3931681	Pool=1
IS613_MH025900	1046	1247	AMPL3931682	Pool=2
ISAPI1_MF175187	24	233	AMPL3931683	Pool=1
ISAPI1_MF175187	222	426	AMPL3931684	Pool=2
ISAPI1_MF175187	415	555	AMPL3931685	Pool=1

ISAPI1_MF175187	544	662	AMPL3931686	Pool=2
ISAPI1_MF175187	651	873	AMPL3931687	Pool=1
ISAba125_NC_022740	51	155	AMPL3932028	Pool=1
ISAba125_NC_022740	144	364	AMPL3932029	Pool=2
ISAba125_NC_022740	353	568	AMPL3932030	Pool=1
ISAba125_NC_022740	557	767	AMPL3932031	Pool=2
ISAba125_NC_022740	756	950	AMPL3932032	Pool=1
QnrA1_1_AY070235	42	249	AMPL3931106	Pool=1
QnrA1_1_AY070235	238	454	AMPL3931107	Pool=2
QnrA1_1_AY070235	443	625	AMPL3931108	Pool=1
QnrB1_1_EF682133	54	182	AMPL3931109	Pool=1
QnrB1_1_EF682133	171	393	AMPL3931110	Pool=2
QnrB1_1_EF682133	382	605	AMPL3931111	Pool=1
QnrB4_1_DQ303921	58	154	AMPL3931112	Pool=1
QnrB4_1_DQ303921	143	365	AMPL3931113	Pool=2
QnrB4_1_DQ303921	354	582	AMPL3931114	Pool=1
QnrB8_1_EU043312	25	160	AMPL3931115	Pool=2
QnrB8_1_EU043312	149	373	AMPL3931116	Pool=1
QnrB8_1_EU043312	362	580	AMPL3931117	Pool=2
QnrC_1_EU917444	63	212	AMPL3931118	Pool=1
QnrC_1_EU917444	201	377	AMPL3931119	Pool=2
QnrC_1_EU917444	366	594	AMPL3931120	Pool=1
QnrD_1_FJ228229	36	223	AMPL3931121	Pool=2
QnrD_1_FJ228229	212	420	AMPL3931122	Pool=1
QnrD_1_FJ228229	409	575	AMPL3931123	Pool=2
QnrS1_1_AB187515	35	187	AMPL3931124	Pool=1

QnrS1_1_AB187515	176	375	AMPL3931125	Pool=2
QnrS1_1_AB187515	364	587	AMPL3931126	Pool=1
QnrS2_1_JF261185	22	225	AMPL3931127	Pool=2
QnrS2_1_JF261185	214	374	AMPL3931128	Pool=1
QnrS2_1_JF261185	363	587	AMPL3931129	Pool=2
QnrVC1_1_EU436855	52	189	AMPL3931130	Pool=1
QnrVC1_1_EU436855	178	380	AMPL3931131	Pool=2
QnrVC1_1_EU436855	369	587	AMPL3931132	Pool=1
RE_cmeA_KT778507	55	191	AMPL3931590	Pool=2
RE_cmeA_KT778507	180	390	AMPL3931591	Pool=1
RE_cmeA_KT778507	373	558	AMPL3931592	Pool=2
RE_cmeA_KT778507	544	733	AMPL3931593	Pool=1
RE_cmeA_KT778507	804	888	AMPL3931595	Pool=2
RE_cmeA_KT778507	862	1072	AMPL3931596	Pool=1
RE_cmeB_KT778507	50	125	AMPL3931597	Pool=2
RE_cmeB_KT778507	114	326	AMPL3931598	Pool=1
RE_cmeB_KT778507	315	521	AMPL3931599	Pool=2
RE_cmeB_KT778507	519	686	AMPL3931600	Pool=1
RE_cmeB_KT778507	675	880	AMPL3931601	Pool=2
RE_cmeB_KT778507	869	1032	AMPL3931602	Pool=1
RE_cmeB_KT778507	1070	1214	AMPL3931604	Pool=2
RE_cmeB_KT778507	1200	1344	AMPL3931605	Pool=1
RE_cmeB_KT778507	1333	1546	AMPL3931606	Pool=2
RE_cmeB_KT778507	1512	1704	AMPL3931607	Pool=1
RE_cmeB_KT778507	1693	1883	AMPL3931608	Pool=2
RE_cmeB_KT778507	1872	2083	AMPL3931609	Pool=1

RE_cmeB_KT778507	2072	2258	AMPL3931610	Pool=2
RE_cmeB_KT778507	2237	2440	AMPL3931611	Pool=1
RE_cmeB_KT778507	2429	2515	AMPL3931612	Pool=2
RE_cmeB_KT778507	2504	2725	AMPL3931613	Pool=1
RE_cmeB_KT778507	2714	2939	AMPL3931614	Pool=2
RE_cmeC_KT778507	44	159	AMPL3929745	Pool=1
RE_cmeC_KT778507	148	298	AMPL3931619	Pool=2
RE_cmeC_KT778507	287	472	AMPL3931620	Pool=1
RE_cmeC_KT778507	461	671	AMPL3931621	Pool=2
RE_cmeC_KT778507	660	859	AMPL3931622	Pool=1
RE_cmeC_KT778507	848	1045	AMPL3931623	Pool=2
RE_cmeC_KT778507	1034	1247	AMPL3931624	Pool=1
RE_cmeC_KT778507	1236	1446	AMPL3931625	Pool=2
Tn21_KY913897	50	253	AMPL3931688	Pool=1
Tn21_KY913897	85	311	AMPL3931689	Pool=2
Tp614_DQ831146	46	260	AMPL3931706	Pool=1
Tp614_DQ831146	248	469	AMPL3931707	Pool=2
Tp614_DQ831146	458	687	AMPL3931708	Pool=1
Tp614_DQ831146	678	856	AMPL3931709	Pool=2
Tp614_DQ831146	845	1071	AMPL3931710	Pool=1
Tp614_DQ831146	1060	1254	AMPL3931711	Pool=2
VanB_1_AF192329	59	255	AMPL3931269	Pool=1
VanB_1_AF192329	244	382	AMPL3931270	Pool=2
VanB_1_AF192329	371	574	AMPL3931271	Pool=1
VanB_1_AF192329	563	786	AMPL3931272	Pool=2
VanB_1_AF192329	775	986	AMPL3931273	Pool=1

VanC_3_EU151752	44	268	AMPL3931278	Pool=2
VanC_3_EU151752	257	478	AMPL3931279	Pool=1
VanC_3_EU151752	467	589	AMPL3931280	Pool=2
VanC_3_EU151752	578	802	AMPL3931281	Pool=1
VanC_3_EU151752	791	1005	AMPL3931282	Pool=2
VanG_1_AY271782	41	235	AMPL3931283	Pool=1
VanG_1_AY271782	224	411	AMPL3931284	Pool=2
VanG_1_AY271782	400	619	AMPL3931285	Pool=1
VanG_1_AY271782	608	826	AMPL3931286	Pool=2
VanG_1_AY271782	815	980	AMPL3931287	Pool=1
VanHB_1_AF192329	47	273	AMPL3931288	Pool=1
VanHB_1_AF192329	262	437	AMPL3931289	Pool=2
VanHB_1_AF192329	420	630	AMPL3931290	Pool=1
VanHB_1_AF192329	608	810	AMPL3931291	Pool=2
VanHB_1_AF192329	799	924	AMPL3931292	Pool=1
VanHD_1_AB242319	38	238	AMPL3931293	Pool=2
VanHD_1_AB242319	227	365	AMPL3931294	Pool=1
VanHD_1_AB242319	354	568	AMPL3931295	Pool=2
VanHD_1_AB242319	557	700	AMPL3931296	Pool=1
VanHD_1_AB242319	689	907	AMPL3931297	Pool=2
VanM_AF288163	50	241	AMPL3932015	Pool=1
VanM_AF288163	230	417	AMPL3932016	Pool=2
VanM_AF288163	406	583	AMPL3932017	Pool=1
VanM_AF288163	572	794	AMPL3932018	Pool=2
VanM_AF288163	783	1000	AMPL3932019	Pool=1
VanM_AF288163	989	1158	AMPL3932020	Pool=2

VanRA_1_FJ866609	45	257	AMPL3931298	Pool=1
VanRA_1_FJ866609	246	416	AMPL3931299	Pool=2
VanRA_1_FJ866609	405	625	AMPL3931300	Pool=1
VanRB_1_AF192329	26	249	AMPL3931301	Pool=1
VanRB_1_AF192329	238	449	AMPL3931302	Pool=2
VanRB_1_AF192329	438	604	AMPL3931303	Pool=1
VanRC_1_AF162694	44	251	AMPL3931304	Pool=2
VanRC_1_AF162694	240	406	AMPL3931305	Pool=1
VanRC_1_AF162694	395	624	AMPL3931306	Pool=2
VanRD_1_AB242319	38	208	AMPL3931310	Pool=1
VanRD_1_AB242319	197	423	AMPL3931311	Pool=2
VanRD_1_AB242319	412	627	AMPL3931312	Pool=1
VanSA_M97297	53	177	AMPL3932009	Pool=2
VanSA_M97297	166	300	AMPL3932010	Pool=1
VanSA_M97297	289	507	AMPL3932011	Pool=2
VanSA_M97297	496	721	AMPL3932012	Pool=1
VanSA_M97297	710	932	AMPL3932013	Pool=2
VanSA_M97297	921	1100	AMPL3932014	Pool=1
VanSB_1_AF192329	30	242	AMPL3931313	Pool=2
VanSB_1_AF192329	231	414	AMPL3931314	Pool=1
VanSB_1_AF192329	403	605	AMPL3931315	Pool=2
VanSB_1_AF192329	594	761	AMPL3931316	Pool=1
VanSB_1_AF192329	750	966	AMPL3931317	Pool=2
VanSB_1_AF192329	955	1122	AMPL3931318	Pool=1
VanSB_1_AF192329	1111	1297	AMPL3931319	Pool=2
VanTC_1_AF162694	70	274	AMPL3931329	Pool=1

VanTC_1_AF162694	263	486	AMPL3931330	Pool=2
VanTC_1_AF162694	475	628	AMPL3931331	Pool=1
VanTC_1_AF162694	617	838	AMPL3931332	Pool=2
VanTC_1_AF162694	827	1052	AMPL3931333	Pool=1
VanTC_1_AF162694	1041	1238	AMPL3931334	Pool=2
VanTC_1_AF162694	1227	1416	AMPL3931335	Pool=1
VanTC_1_AF162694	1405	1624	AMPL3931336	Pool=2
VanTC_1_AF162694	1613	1831	AMPL3931337	Pool=1
VanTC_1_AF162694	1823	2033	AMPL3931338	Pool=2
VanTG_1_AY271782	69	288	AMPL3931350	Pool=1
VanTG_1_AY271782	277	395	AMPL3931351	Pool=2
VanTG_1_AY271782	384	590	AMPL3931352	Pool=1
VanTG_1_AY271782	572	771	AMPL3931353	Pool=2
VanTG_1_AY271782	760	955	AMPL3931354	Pool=1
VanTG_1_AY271782	944	1163	AMPL3931355	Pool=2
VanTG_1_AY271782	1152	1376	AMPL3931356	Pool=1
VanTG_1_AY271782	1365	1565	AMPL3931357	Pool=2
VanTG_1_AY271782	1554	1779	AMPL3931358	Pool=1
VanTG_1_AY271782	1768	1858	AMPL3931359	Pool=2
VanTG_1_AY271782	1847	2069	AMPL3931360	Pool=1
VanWB_1_AF192329	28	169	AMPL3931361	Pool=1
VanWB_1_AF192329	158	378	AMPL3931362	Pool=2
VanWB_1_AF192329	367	559	AMPL3931363	Pool=1
VanWB_1_AF192329	548	759	AMPL3931364	Pool=2
VanWG_1_AY271782	53	271	AMPL3931365	Pool=1
VanWG_1_AY271782	260	446	AMPL3931366	Pool=2

VanWG_1_AY271782	431	597	AMPL3931367	Pool=1
VanWG_1_AY271782	586	779	AMPL3931368	Pool=2
VanXA_1_FJ866609	49	147	AMPL3929264	Pool=1
VanXA_1_FJ866609	136	359	AMPL3931369	Pool=2
VanXA_1_FJ866609	348	540	AMPL3931370	Pool=1
VanXB_1_AF192329	44	214	AMPL3931371	Pool=1
VanXB_1_AF192329	203	355	AMPL3931372	Pool=2
VanXB_1_AF192329	344	539	AMPL3931373	Pool=1
VanXD_1_AB242319	36	257	AMPL3931374	Pool=1
VanXD_1_AB242319	246	414	AMPL3931375	Pool=2
VanXD_1_AB242319	398	539	AMPL3931376	Pool=1
VanYB_1_AF192329	33	199	AMPL3931377	Pool=2
VanYB_1_AF192329	188	372	AMPL3931378	Pool=1
VanYB_1_AF192329	361	588	AMPL3931379	Pool=2
VanYB_1_AF192329	577	749	AMPL3931380	Pool=1
VanYD_1_AB242319	28	194	AMPL3931381	Pool=2
VanYD_1_AB242319	183	408	AMPL3931382	Pool=1
VanYD_1_AB242319	397	582	AMPL3931383	Pool=2
VanYD_1_AB242319	571	788	AMPL3931384	Pool=1
VanYD_1_AB242319	777	1004	AMPL3931385	Pool=2
aac6_II_L12710	67	278	AMPL3931489	Pool=1
aac6_II_L12710	267	492	AMPL3931490	Pool=2
aac6_Ia_1_M18967	34	103	AMPL3930982	Pool=1
aac6_Ia_1_M18967	92	286	AMPL3930983	Pool=2
aac6_Ia_1_M18967	275	490	AMPL3930984	Pool=1
aac6_Ib_JN108884	66	291	AMPL3931470	Pool=1

aac6_Ib_JN108884	280	501	AMPL3931471	Pool=2
aac6_Iy_AF144880	25	223	AMPL3931487	Pool=1
aac6_Iy_AF144880	212	368	AMPL3931488	Pool=2
aacA_aphD_GU565967	87	190	AMPL3929480	Pool=1
aacA_aphD_GU565967	179	381	AMPL3931472	Pool=2
aacA_aphD_GU565967	342	421	AMPL3931473	Pool=1
aacA_aphD_GU565967	646	848	AMPL3931475	Pool=2
aacA_aphD_GU565967	656	868	AMPL3931476	Pool=1
aacA_aphD_GU565967	1082	1235	AMPL3931478	Pool=2
aacA_aphD_GU565967	1195	1384	AMPL3931479	Pool=1
aacC1_U04610	59	192	AMPL3931402	Pool=2
aacC1_U04610	181	409	AMPL3931403	Pool=1
aacC1_U04610	398	482	AMPL3929333	Pool=2
aacC2_AY138987	26	188	AMPL3931492	Pool=1
aacC2_AY138987	174	375	AMPL3931493	Pool=2
aacC2_AY138987	309	536	AMPL3931494	Pool=1
aacC2_AY138987	507	617	AMPL3931495	Pool=2
aacC2_AY138987	606	839	AMPL3931496	Pool=1
aac_1_AJ628983	70	286	AMPL3930980	Pool=1
aac_1_AJ628983	275	503	AMPL3931491	Pool=2
aadA1_KR610434	59	283	AMPL3931503	Pool=1
aadA1_KR610434	272	482	AMPL3931504	Pool=2
aadA1_KR610434	471	685	AMPL3931505	Pool=1
aadA1_KR610434	674	759	AMPL3929539	Pool=2
aadA2_2_JQ364967	28	154	AMPL3928543	Pool=1
aadA2_2_JQ364967	143	346	AMPL3930988	Pool=2

aadA2_2_JQ364967	335	498	AMPL3930989	Pool=1
aadA2_2_JQ364967	487	685	AMPL3930990	Pool=2
aadA2_2_JQ364967	674	752	AMPL3928550	Pool=1
aadA5_KT175895	24	194	AMPL3931506	Pool=2
aadA5_KT175895	152	376	AMPL3931507	Pool=1
aadA5_KT175895	365	591	AMPL3931508	Pool=2
aadA5_KT175895	580	743	AMPL3931509	Pool=1
aadA9_1_AJ420072	26	227	AMPL3930991	Pool=2
aadA9_1_AJ420072	216	417	AMPL3930992	Pool=1
aadA9_1_AJ420072	406	559	AMPL3930993	Pool=2
aadA9_1_AJ420072	548	772	AMPL3930994	Pool=1
aadA_MH257908	68	277	AMPL3931497	Pool=1
aadA_MH257908	265	411	AMPL3931498	Pool=2
aadA_MH257908	400	607	AMPL3931499	Pool=1
aadA_MH257908	596	734	AMPL3931500	Pool=2
aadD_1_AF181950	61	270	AMPL3930995	Pool=1
aadD_1_AF181950	259	481	AMPL3930996	Pool=2
aadD_1_AF181950	473	701	AMPL3930997	Pool=1
aadE_AB699882	53	210	AMPL3931510	Pool=2
aadE_AB699882	199	421	AMPL3931511	Pool=1
aadE_AB699882	410	484	AMPL3931512	Pool=2
aadE_AB699882	473	689	AMPL3931513	Pool=1
aadE_AB699882	678	863	AMPL3931514	Pool=2
adeA_KX154813	29	149	AMPL3931515	Pool=1
adeA_KX154813	138	343	AMPL3931516	Pool=2
adeA_KX154813	332	538	AMPL3931517	Pool=1

adeA_KX154813	527	734	AMPL3931518	Pool=2
adeA_KX154813	723	943	AMPL3931519	Pool=1
adeA_KX154813	932	1143	AMPL3931520	Pool=2
aph2_Id_1_AF016483	32	252	AMPL3930998	Pool=1
aph2_Id_1_AF016483	241	317	AMPL3930999	Pool=2
aph2_Id_1_AF016483	306	507	AMPL3931000	Pool=1
aph2_Id_1_AF016483	502	694	AMPL3931001	Pool=2
aph2_Id_1_AF016483	683	844	AMPL3931002	Pool=1
aph6_Ia_1_AY971801	19	211	AMPL3931003	Pool=1
aph6_Ia_1_AY971801	140	330	AMPL3931004	Pool=2
aph6_Ia_1_AY971801	314	497	AMPL3931005	Pool=1
aph6_Ia_1_AY971801	486	723	AMPL3931006	Pool=2
aph6_Ia_1_AY971801	719	889	AMPL3931007	Pool=1
aphA1_HM146784	25	204	AMPL3931521	Pool=2
aphA1_HM146784	193	420	AMPL3931522	Pool=1
aphA1_HM146784	409	569	AMPL3931523	Pool=2
aphA1_HM146784	558	772	AMPL3931524	Pool=1
armA_1_AY220558	33	230	AMPL3931011	Pool=2
armA_1_AY220558	189	397	AMPL3931012	Pool=1
armA_1_AY220558	376	573	AMPL3931013	Pool=2
armA_1_AY220558	562	717	AMPL3931014	Pool=1
bacA_D85752	78	286	AMPL3931714	Pool=2
bcrB_KX772240	63	230	AMPL3931880	Pool=1
bcrB_KX772240	219	433	AMPL3931881	Pool=2
bcrB_KX772240	422	645	AMPL3931882	Pool=1
bcrR_KX772240	53	274	AMPL3931712	Pool=1

bcrR_KX772240	263	489	AMPL3931713	Pool=2
bla1_AF367983	57	196	AMPL3930712	Pool=1
bla1_AF367983	185	399	AMPL3930713	Pool=2
bla1_AF367983	388	549	AMPL3930714	Pool=1
bla1_AF367983	515	702	AMPL3930715	Pool=2
bla1_AF367983	691	807	AMPL3930716	Pool=1
bla1_AF367983	796	1016	AMPL3930717	Pool=2
bla1_AF367983	1005	1184	AMPL3930718	Pool=1
bla1_AF367983	1173	1379	AMPL3930719	Pool=2
bla1_AF367983	1368	1580	AMPL3930720	Pool=1
blaACC_1_NG_048588	34	136	AMPL3930453	Pool=2
blaACC_1_NG_048588	125	351	AMPL3931999	Pool=1
blaACC_1_NG_048588	340	568	AMPL3932000	Pool=2
blaACC_1_NG_048588	557	730	AMPL3932001	Pool=1
blaACC_1_NG_048588	719	941	AMPL3932002	Pool=2
blaACC_1_NG_048588	930	1132	AMPL3932003	Pool=1
blaAIM_1_AM998375	425	577	AMPL3930721	Pool=1
blaAIM_1_AM998375	591	823	AMPL3930722	Pool=2
blaAIM_1_AM998375	832	1071	AMPL3930723	Pool=1
blaAIM_1_AM998375	1060	1146	AMPL3930724	Pool=2
blaAIM_1_AM998375	1135	1308	AMPL3930725	Pool=1
blaAIM_1_AM998375	1297	1514	AMPL3930726	Pool=2
blaAIM_1_AM998375	1500	1604	AMPL3930727	Pool=1
blaAIM_1_AM998375	1576	1802	AMPL3930728	Pool=2
blaAIM_1_AM998375	1791	1969	AMPL3930729	Pool=1
blaAIM_1_AM998375	1909	2077	AMPL3930730	Pool=2

blaAIM_1_AM998375	2094	2248	AMPL3930732	Pool=1
blaBIC_GQ260093	23	145	AMPL3930740	Pool=2
blaBIC_GQ260093	134	299	AMPL3930741	Pool=1
blaBIC_GQ260093	288	488	AMPL3930742	Pool=2
blaBIC_GQ260093	477	631	AMPL3930743	Pool=1
blaBIC_GQ260093	706	822	AMPL3928060	Pool=2
blaCMY_1_X92508	23	246	AMPL3931896	Pool=1
blaCMY_1_X92508	203	358	AMPL3931897	Pool=2
blaCMY_1_X92508	336	442	AMPL3931898	Pool=1
blaCMY_1_X92508	461	662	AMPL3931900	Pool=2
blaCMY_1_X92508	650	768	AMPL3931901	Pool=1
blaCMY_1_X92508	751	926	AMPL3931902	Pool=2
blaCMY_1_X92508	915	1122	AMPL3931903	Pool=1
blaCTX_M_1_6_DQ915955	69	276	AMPL3930745	Pool=1
blaCTX_M_1_6_DQ915955	265	416	AMPL3930746	Pool=2
blaCTX_M_1_6_DQ915955	406	628	AMPL3930747	Pool=1
blaCTX_M_1_6_DQ915955	605	820	AMPL3930748	Pool=2
blaCTX_M_9_HM569735	62	291	AMPL3930749	Pool=1
blaCTX_M_9_HM569735	275	479	AMPL3930750	Pool=2
blaCTX_M_9_HM569735	457	673	AMPL3930751	Pool=1
blaCTX_M_9_HM569735	663	887	AMPL3930752	Pool=2
blaCTX_M_9_HM569735	876	1101	AMPL3930753	Pool=1
blaCTX_M_9_HM569735	1090	1313	AMPL3930754	Pool=2
blaCTX_M_9_HM569735	1302	1424	AMPL3928083	Pool=1
blaCTX_M_9_HM569735	1413	1627	AMPL3930755	Pool=2
blaCTX_M_9_HM569735	1621	1846	AMPL3930756	Pool=1

blaCTX_M_9_HM569735	1828	1979	AMPL3930757	Pool=2
blaDHA_1_Y16410	33	184	AMPL3931906	Pool=1
blaDHA_1_Y16410	173	364	AMPL3931907	Pool=2
blaDHA_1_Y16410	319	525	AMPL3931908	Pool=1
blaDHA_1_Y16410	514	733	AMPL3931909	Pool=2
blaDHA_1_Y16410	721	893	AMPL3931910	Pool=1
blaDHA_1_Y16410	882	1060	AMPL3931911	Pool=2
blaGES_1_HQ170511	24	217	AMPL3931912	Pool=1
blaGES_1_HQ170511	206	419	AMPL3931913	Pool=2
blaGES_1_HQ170511	408	538	AMPL3931914	Pool=1
blaGES_1_HQ170511	527	676	AMPL3931915	Pool=2
blaGES_1_HQ170511	665	792	AMPL3930308	Pool=1
blaGIM_1_JF414726	64	285	AMPL3931916	Pool=1
blaGIM_1_JF414726	274	491	AMPL3931917	Pool=2
blaGIM_1_JF414726	480	701	AMPL3931918	Pool=1
blaIMP_1_DQ522237	65	285	AMPL3931919	Pool=2
blaIMP_1_DQ522237	275	495	AMPL3931920	Pool=1
blaIMP_1_DQ522237	487	671	AMPL3931921	Pool=2
blaKPC_1_AF297554	23	205	AMPL3931922	Pool=1
blaKPC_1_AF297554	178	402	AMPL3931923	Pool=2
blaKPC_1_AF297554	393	610	AMPL3931924	Pool=1
blaKPC_1_AF297554	582	715	AMPL3931925	Pool=2
blaKPC_1_AF297554	702	860	AMPL3931926	Pool=1
blaL1_3_EF126059	48	281	AMPL3930758	Pool=1
blaL1_3_EF126059	267	470	AMPL3930759	Pool=2
blaL1_3_EF126059	451	630	AMPL3930760	Pool=1

blaL1_3_EF126059	574	744	AMPL3930761	Pool=2
blaL1_3_EF126059	677	819	AMPL3930762	Pool=1
blaOCH_LT840075	30	253	AMPL3930766	Pool=2
blaOCH_LT840075	242	445	AMPL3930767	Pool=1
blaOCH_LT840075	434	602	AMPL3930768	Pool=2
blaOCH_LT840075	591	751	AMPL3930769	Pool=1
blaOCH_LT840075	740	873	AMPL3930770	Pool=2
blaOCH_LT840075	862	1085	AMPL3930771	Pool=1
blaOKP_NG_049360	25	121	AMPL3928115	Pool=2
blaOKP_NG_049360	107	286	AMPL3930772	Pool=1
blaOKP_NG_049360	257	455	AMPL3930773	Pool=2
blaOKP_NG_049360	444	671	AMPL3930774	Pool=1
blaOKP_NG_049360	636	805	AMPL3930775	Pool=2
blaOXA_10_EU708817	31	215	AMPL3931386	Pool=1
blaOXA_10_EU708817	204	402	AMPL3931387	Pool=2
blaOXA_10_EU708817	391	589	AMPL3931388	Pool=1
blaOXA_10_EU708817	578	742	AMPL3931389	Pool=2
blaOXA_23_FJ959346	23	236	AMPL3930776	Pool=1
blaOXA_23_FJ959346	229	371	AMPL3930777	Pool=2
blaOXA_23_FJ959346	360	526	AMPL3930778	Pool=1
blaOXA_23_FJ959346	515	716	AMPL3930779	Pool=2
blaOXA_23_FJ959346	705	920	AMPL3930780	Pool=1
blaOXA_23_FJ959346	909	1111	AMPL3930781	Pool=2
blaOXA_48_2_AY236073	29	214	AMPL3930782	Pool=1
blaOXA_48_2_AY236073	203	428	AMPL3930783	Pool=2
blaOXA_48_2_AY236073	417	592	AMPL3930784	Pool=1

blaOXA_48_2_AY236073	581	751	AMPL3930785	Pool=2
blaOXA_51_AJ309734	61	170	AMPL3929334	Pool=1
blaOXA_51_AJ309734	159	363	AMPL3931404	Pool=2
blaOXA_51_AJ309734	352	559	AMPL3931405	Pool=1
blaOXA_51_AJ309734	548	754	AMPL3931406	Pool=2
blaOXA_58_1_AY665723	69	196	AMPL3928143	Pool=1
blaOXA_58_1_AY665723	185	381	AMPL3930786	Pool=2
blaOXA_58_1_AY665723	370	580	AMPL3930787	Pool=1
blaOXA_58_1_AY665723	569	779	AMPL3930788	Pool=2
blaOXY_1_Z30177	23	195	AMPL3931927	Pool=1
blaOXY_1_Z30177	180	276	AMPL3931928	Pool=2
blaOXY_1_Z30177	265	477	AMPL3931929	Pool=1
blaOXY_1_Z30177	450	625	AMPL3931930	Pool=2
blaOXY_1_Z30177	644	818	AMPL3931932	Pool=1
blaPAO_1_AY083595	56	275	AMPL3930789	Pool=1
blaPAO_1_AY083595	264	454	AMPL3930790	Pool=2
blaPAO_1_AY083595	442	631	AMPL3930791	Pool=1
blaPAO_1_AY083595	618	780	AMPL3930792	Pool=2
blaPAO_1_AY083595	801	1015	AMPL3930794	Pool=1
blaPAO_1_AY083595	859	1085	AMPL3930795	Pool=2
blaPER_1_GU944725	44	265	AMPL3931933	Pool=1
blaPER_1_GU944725	254	479	AMPL3931934	Pool=2
blaPER_1_GU944725	468	697	AMPL3931935	Pool=1
blaPER_1_GU944725	686	868	AMPL3931936	Pool=2
blaPER_2_X93314	58	273	AMPL3931937	Pool=1
blaPER_2_X93314	260	484	AMPL3931938	Pool=2

blaPER_2_X93314	473	700	AMPL3931939	Pool=1
blaPER_2_X93314	689	898	AMPL3931940	Pool=2
blaPSE_1_M69058	28	211	AMPL3930797	Pool=1
blaPSE_1_M69058	200	423	AMPL3930798	Pool=2
blaPSE_1_M69058	412	630	AMPL3930799	Pool=1
blaPSE_1_M69058	619	753	AMPL3930800	Pool=2
blaPSE_1_M69058	742	894	AMPL3930801	Pool=1
blaPSE_1_M69058	883	1049	AMPL3930802	Pool=2
blaROB_1_DQ840517	70	292	AMPL3931941	Pool=1
blaROB_1_DQ840517	276	474	AMPL3931942	Pool=2
blaROB_1_DQ840517	463	652	AMPL3931943	Pool=1
blaROB_1_DQ840517	641	859	AMPL3931944	Pool=2
blaSFO_1_AB003148	38	171	AMPL3931390	Pool=1
blaSFO_1_AB003148	160	380	AMPL3931391	Pool=2
blaSFO_1_AB003148	369	578	AMPL3931392	Pool=1
blaSFO_1_AB003148	567	781	AMPL3931393	Pool=2
blaSFO_1_AB003148	770	847	AMPL3929310	Pool=1
blaSHV_1_AF148850	27	205	AMPL3930803	Pool=1
blaSHV_1_AF148850	196	429	AMPL3930804	Pool=2
blaSHV_1_AF148850	391	488	AMPL3930805	Pool=1
blaSHV_1_AF148850	471	676	AMPL3930806	Pool=2
blaSHV_1_AF148850	635	807	AMPL3930807	Pool=1
blaSIM_1_JF731030	51	159	AMPL3931945	Pool=1
blaSIM_1_JF731030	148	376	AMPL3931946	Pool=2
blaSIM_1_JF731030	365	560	AMPL3931947	Pool=1
blaSIM_1_JF731030	549	676	AMPL3931948	Pool=2

blaSPM_1_AY341249	36	213	AMPL3931949	Pool=1
blaSPM_1_AY341249	202	412	AMPL3931950	Pool=2
blaSPM_1_AY341249	401	589	AMPL3931951	Pool=1
blaTEM_1A_4_HM749966	33	182	AMPL3930808	Pool=1
blaTEM_1A_4_HM749966	171	398	AMPL3930809	Pool=2
blaTEM_1A_4_HM749966	387	604	AMPL3930810	Pool=1
blaTEM_1A_4_HM749966	593	791	AMPL3930811	Pool=2
blaTLA_1_AF148067	73	201	AMPL3931952	Pool=1
blaTLA_1_AF148067	190	365	AMPL3931953	Pool=2
blaTLA_1_AF148067	354	528	AMPL3931954	Pool=1
blaTLA_1_AF148067	517	723	AMPL3931955	Pool=2
blaTLA_1_AF148067	712	875	AMPL3931956	Pool=1
blaVEB_1_HM370393	72	164	AMPL3930383	Pool=1
blaVEB_1_HM370393	153	323	AMPL3931959	Pool=2
blaVEB_1_HM370393	312	513	AMPL3931960	Pool=1
blaVEB_1_HM370393	502	687	AMPL3931961	Pool=2
blaVEB_1_HM370393	676	852	AMPL3931962	Pool=1
blaVIM_2_KC907378	47	229	AMPL3931394	Pool=2
blaVIM_2_KC907378	218	444	AMPL3931395	Pool=1
blaVIM_2_KC907378	307	535	AMPL3931396	Pool=2
blaVIM_2_KC907378	524	750	AMPL3931397	Pool=1
blaZ_32_AP004832	68	159	AMPL3928189	Pool=2
blaZ_32_AP004832	148	314	AMPL3930812	Pool=1
blaZ_32_AP004832	298	410	AMPL3930813	Pool=2
blaZ_32_AP004832	531	616	AMPL3928196	Pool=1
carB_HQ616211	104	291	AMPL3931989	Pool=2

carB_HQ616211	280	499	AMPL3931990	Pool=1
carB_HQ616211	488	626	AMPL3931991	Pool=2
carB_HQ616211	615	826	AMPL3931992	Pool=1
carB_HQ616211	815	1005	AMPL3931993	Pool=2
carB_HQ616211	994	1186	AMPL3931994	Pool=1
carB_HQ616211	1175	1338	AMPL3931995	Pool=2
carB_HQ616211	1327	1510	AMPL3931996	Pool=1
catA1_1_V00622	35	244	AMPL3931209	Pool=1
catA1_1_V00622	233	381	AMPL3931210	Pool=2
catA1_1_V00622	370	594	AMPL3931211	Pool=1
catB3_1_AJ009818	44	271	AMPL3931212	Pool=2
catB3_1_AJ009818	260	465	AMPL3931213	Pool=1
catB3_1_AJ009818	454	573	AMPL3928965	Pool=2
catB8_1_AF227506	29	209	AMPL3931214	Pool=1
catB8_1_AF227506	198	406	AMPL3931215	Pool=2
catB8_1_AF227506	395	581	AMPL3931216	Pool=1
ceoA_U97042	32	255	AMPL3931716	Pool=2
ceoA_U97042	218	445	AMPL3931717	Pool=1
ceoA_U97042	434	658	AMPL3931718	Pool=2
ceoA_U97042	647	781	AMPL3931719	Pool=1
ceoA_U97042	761	998	AMPL3931720	Pool=2
ceoA_U97042	987	1198	AMPL3931721	Pool=1
cepA_1_L13472	64	255	AMPL3930815	Pool=2
cepA_1_L13472	244	463	AMPL3930816	Pool=1
cepA_1_L13472	452	661	AMPL3930817	Pool=2
cepA_1_L13472	649	833	AMPL3930818	Pool=1

cfr_1_AM408573	68	173	AMPL3928971	Pool=1
cfr_1_AM408573	162	289	AMPL3931217	Pool=2
cfr_1_AM408573	278	489	AMPL3931218	Pool=1
cfr_1_AM408573	478	599	AMPL3931219	Pool=2
cfr_1_AM408573	576	796	AMPL3931220	Pool=1
cfr_1_AM408573	785	865	AMPL3928979	Pool=2
cmeA_AB894099	55	183	AMPL3931626	Pool=1
cmeA_AB894099	174	368	AMPL3931627	Pool=2
cmeA_AB894099	357	559	AMPL3931628	Pool=1
cmeA_AB894099	544	734	AMPL3931629	Pool=2
cmeA_AB894099	789	886	AMPL3931631	Pool=1
cmeA_AB894099	861	1069	AMPL3931632	Pool=2
cmeB_AB894099	42	173	AMPL3931633	Pool=1
cmeB_AB894099	162	374	AMPL3931634	Pool=2
cmeB_AB894099	363	547	AMPL3931635	Pool=1
cmeB_AB894099	536	700	AMPL3931636	Pool=2
cmeB_AB894099	689	909	AMPL3931637	Pool=1
cmeB_AB894099	898	1114	AMPL3931638	Pool=2
cmeB_AB894099	1103	1291	AMPL3931639	Pool=1
cmeB_AB894099	1280	1494	AMPL3931640	Pool=2
cmeB_AB894099	1483	1703	AMPL3931641	Pool=1
cmeB_AB894099	1692	1887	AMPL3931642	Pool=2
cmeB_AB894099	1876	2069	AMPL3931643	Pool=1
cmeB_AB894099	2058	2273	AMPL3931644	Pool=2
cmeB_AB894099	2257	2399	AMPL3931645	Pool=1
cmeB_AB894099	2388	2600	AMPL3931646	Pool=2

cmeB_AB894099	2592	2807	AMPL3931647	Pool=1
cmeB_AB894099	2796	2942	AMPL3931648	Pool=2
cmeC_AB894099	54	267	AMPL3931652	Pool=1
cmeC_AB894099	263	480	AMPL3931653	Pool=2
cmeC_AB894099	469	668	AMPL3931654	Pool=1
cmeC_AB894099	657	868	AMPL3931655	Pool=2
cmeC_AB894099	857	994	AMPL3931656	Pool=1
cmeC_AB894099	983	1202	AMPL3931657	Pool=2
cmeC_AB894099	1191	1402	AMPL3931658	Pool=1
cmlA1_1_M64556	36	198	AMPL3931221	Pool=1
cmlA1_1_M64556	190	419	AMPL3931222	Pool=2
cmlA1_1_M64556	416	647	AMPL3931223	Pool=1
cmlA1_1_M64556	646	867	AMPL3931224	Pool=2
cmlA1_1_M64556	856	1054	AMPL3931225	Pool=1
cmlA1_1_M64556	1024	1220	AMPL3931226	Pool=2
cmr_U44900	54	196	AMPL3931723	Pool=1
cmr_U44900	185	346	AMPL3931724	Pool=2
cmr_U44900	332	549	AMPL3931725	Pool=1
cmr_U44900	539	754	AMPL3931726	Pool=2
cmr_U44900	743	882	AMPL3931727	Pool=1
cmr_U44900	871	1033	AMPL3931728	Pool=2
cmr_U44900	1022	1199	AMPL3931729	Pool=1
cmx_1_U85507	58	257	AMPL3931227	Pool=1
cmx_1_U85507	234	320	AMPL3931228	Pool=2
cmx_1_U85507	314	514	AMPL3931229	Pool=1
cmx_1_U85507	503	738	AMPL3931230	Pool=2

cmx_1_U85507	678	901	AMPL3931231	Pool=1
cmx_1_U85507	890	1115	AMPL3931232	Pool=2
dfrA1_MH574896	39	201	AMPL3932021	Pool=1
dfrA1_MH574896	190	416	AMPL3932022	Pool=2
dfrA_GU565967	30	131	AMPL3931536	Pool=1
dfrA_GU565967	120	298	AMPL3931537	Pool=2
dfrA_GU565967	287	439	AMPL3931538	Pool=1
dfrK_FN390947	39	260	AMPL3931539	Pool=1
dfrK_FN390947	249	462	AMPL3931540	Pool=2
drfA12_JN108892	22	236	AMPL3931663	Pool=1
drfA12_JN108892	223	442	AMPL3931664	Pool=2
ereA_2_AF099140	67	292	AMPL3930830	Pool=1
ereA_2_AF099140	281	508	AMPL3930831	Pool=2
ereA_2_AF099140	497	641	AMPL3930832	Pool=1
ereA_2_AF099140	630	809	AMPL3930833	Pool=2
ereA_2_AF099140	798	1023	AMPL3930834	Pool=1
ereA_2_AF099140	1012	1156	AMPL3930835	Pool=2
ereB_2_X03988	22	226	AMPL3930836	Pool=1
ereB_2_X03988	215	425	AMPL3930837	Pool=2
ereB_2_X03988	414	603	AMPL3930838	Pool=1
ereB_2_X03988	592	814	AMPL3930839	Pool=2
ereB_2_X03988	803	1022	AMPL3930840	Pool=1
ereB_2_X03988	1011	1190	AMPL3930841	Pool=2
erm34_AY234334	54	270	AMPL3931407	Pool=1
erm34_AY234334	259	471	AMPL3931408	Pool=2
erm34_AY234334	460	637	AMPL3931409	Pool=1

erm34_AY234334	626	798	AMPL3931410	Pool=2
erm35_AF319779	54	217	AMPL3931411	Pool=1
erm35_AF319779	206	421	AMPL3931412	Pool=2
erm35_AF319779	411	614	AMPL3931413	Pool=1
erm35_AF319779	531	753	AMPL3931414	Pool=2
erm36_AF462611	60	221	AMPL3931415	Pool=1
erm36_AF462611	196	419	AMPL3931416	Pool=2
erm36_AF462611	375	494	AMPL3931417	Pool=1
erm36_AF462611	483	642	AMPL3931418	Pool=2
erm36_AF462611	628	786	AMPL3931419	Pool=1
erm36_AF462611	775	963	AMPL3931420	Pool=2
erm36_AF462611	952	1118	AMPL3931421	Pool=1
ermA_1_X03216	34	161	AMPL3930842	Pool=1
ermA_1_X03216	150	372	AMPL3930843	Pool=2
ermA_1_X03216	361	437	AMPL3928258	Pool=1
ermA_1_X03216	426	642	AMPL3930844	Pool=2
ermB_18_X66468	29	139	AMPL3928264	Pool=1
ermB_18_X66468	128	316	AMPL3930847	Pool=2
ermB_18_X66468	305	500	AMPL3930848	Pool=1
ermB_18_X66468	489	692	AMPL3930849	Pool=2
ermC_1_V01278	31	207	AMPL3930850	Pool=1
ermC_1_V01278	196	408	AMPL3930851	Pool=2
ermC_1_V01278	397	497	AMPL3930852	Pool=1
ermC_1_V01278	494	681	AMPL3930853	Pool=2
ermD_1_M29832	69	250	AMPL3930854	Pool=1
ermD_1_M29832	239	380	AMPL3930855	Pool=2

ermD_1_M29832	388	601	AMPL3930856	Pool=1
ermD_1_M29832	623	795	AMPL3930858	Pool=2
ermE_1_X51891	20	250	AMPL3930861	Pool=1
ermE_1_X51891	236	324	AMPL3928291	Pool=2
ermE_1_X51891	312	546	AMPL3930862	Pool=1
ermE_1_X51891	538	714	AMPL3930863	Pool=2
ermE_1_X51891	700	850	AMPL3930864	Pool=1
ermE_1_X51891	839	1025	AMPL3930865	Pool=2
ermF_2_M17124	54	262	AMPL3930985	Pool=1
ermF_2_M17124	251	465	AMPL3930986	Pool=2
ermF_2_M17124	454	673	AMPL3930987	Pool=1
ermF_2_M17124	665	753	AMPL3928542	Pool=2
ermG_1_M15332	117	296	AMPL3930867	Pool=1
ermG_1_M15332	285	483	AMPL3930868	Pool=2
ermG_1_M15332	472	670	AMPL3930869	Pool=1
ermK_M77505	69	179	AMPL3929320	Pool=1
ermK_M77505	168	380	AMPL3931398	Pool=2
ermK_M77505	373	596	AMPL3931399	Pool=1
ermK_M77505	619	813	AMPL3931401	Pool=2
ermT_1_M64090	50	205	AMPL3930871	Pool=1
ermT_1_M64090	195	376	AMPL3930872	Pool=2
ermT_1_M64090	365	531	AMPL3930873	Pool=1
ermT_1_M64090	520	672	AMPL3930874	Pool=2
ermX_1_M36726	44	207	AMPL3930875	Pool=1
ermX_1_M36726	196	369	AMPL3930876	Pool=2
ermX_1_M36726	348	550	AMPL3930877	Pool=1

ermX_1_M36726	468	670	AMPL3930878	Pool=2
ermX_1_M36726	661	790	AMPL3928327	Pool=1
ermY_1_AB014481	125	317	AMPL3930879	Pool=1
ermY_1_AB014481	300	469	AMPL3930880	Pool=2
ermY_1_AB014481	453	662	AMPL3930881	Pool=1
fabK_FJ390165	27	179	AMPL3931730	Pool=1
fabK_FJ390165	168	391	AMPL3931731	Pool=2
fexA_1_AJ549214	73	155	AMPL3931233	Pool=1
fexA_1_AJ549214	144	366	AMPL3931234	Pool=2
fexA_1_AJ549214	355	550	AMPL3931235	Pool=1
fexA_1_AJ549214	539	764	AMPL3931236	Pool=2
fexA_1_AJ549214	753	974	AMPL3931237	Pool=1
fexA_1_AJ549214	963	1183	AMPL3931238	Pool=2
fexA_1_AJ549214	1172	1377	AMPL3931239	Pool=1
floR_1_AF071555	66	194	AMPL3931241	Pool=2
floR_1_AF071555	183	410	AMPL3931242	Pool=1
floR_1_AF071555	332	544	AMPL3931243	Pool=2
floR_1_AF071555	533	638	AMPL3931244	Pool=1
floR_1_AF071555	627	797	AMPL3931245	Pool=2
floR_1_AF071555	786	956	AMPL3931246	Pool=1
floR_1_AF071555	945	1054	AMPL3931247	Pool=2
floR_1_AF071555	1043	1195	AMPL3931248	Pool=1
folA_EU871432	61	271	AMPL3931665	Pool=1
folA_EU871432	260	451	AMPL3931666	Pool=2
fosA3_KX245440	23	179	AMPL3931541	Pool=1
fosA3_KX245440	164	351	AMPL3931542	Pool=2

fosA_M85195	22	225	AMPL3931544	Pool=1
fosA_M85195	212	398	AMPL3931545	Pool=2
fosB_X89875	103	242	AMPL3931546	Pool=1
fosB_X89875	232	374	AMPL3931547	Pool=2
fosX_CC_KC876749	33	153	AMPL3931551	Pool=1
fosX_CC_KC876749	142	346	AMPL3931552	Pool=2
fosX_CC_KC876749	321	395	AMPL3931553	Pool=1
fosX_NG_047899	57	281	AMPL3931550	Pool=2
gyrA_MG995190	59	268	AMPL3931576	Pool=1
gyrA_MG995190	254	444	AMPL3931577	Pool=2
gyrA_MG995190	433	574	AMPL3931578	Pool=1
gyrA_MG995190	565	763	AMPL3931579	Pool=2
gyrA_MG995190	740	930	AMPL3931580	Pool=1
gyrA_MG995190	919	1102	AMPL3931581	Pool=2
gyrA_MG995190	1093	1300	AMPL3931582	Pool=1
gyrA_MG995190	1289	1389	AMPL3931583	Pool=2
gyrA_MG995190	1377	1584	AMPL3931584	Pool=1
gyrA_MG995190	1573	1703	AMPL3929684	Pool=2
gyrA_MG995190	1700	1915	AMPL3931585	Pool=1
gyrA_MG995190	1904	2106	AMPL3931586	Pool=2
gyrA_MG995190	2126	2293	AMPL3931588	Pool=1
gyrA_MG995190	2267	2469	AMPL3931589	Pool=2
imiR_KR057494	25	139	AMPL3931732	Pool=1
imiR_KR057494	128	329	AMPL3931733	Pool=2
imiR_KR057494	318	548	AMPL3931734	Pool=1
imiR_KR057494	537	676	AMPL3931735	Pool=2

imiR_KR057494	665	837	AMPL3931736	Pool=1
intI1_NZ_CP039294	53	217	AMPL3932033	Pool=1
intI1_NZ_CP039294	199	300	AMPL3930518	Pool=2
intI1_NZ_CP039294	275	468	AMPL3932034	Pool=1
intI1_NZ_CP039294	458	686	AMPL3932035	Pool=2
intI1_NZ_CP039294	551	779	AMPL3932036	Pool=1
intI1_NZ_CP039294	768	965	AMPL3932037	Pool=2
lmrA_1_X59926	34	211	AMPL3930886	Pool=1
lmrA_1_X59926	201	351	AMPL3930887	Pool=2
lmrA_1_X59926	442	552	AMPL3930889	Pool=1
lmrA_1_X59926	542	688	AMPL3930890	Pool=2
lmrA_1_X59926	617	826	AMPL3930891	Pool=1
lmrA_1_X59926	908	1143	AMPL3930893	Pool=2
lmrA_1_X59926	1132	1338	AMPL3930894	Pool=1
lnuA_1_M14039	86	243	AMPL3930898	Pool=1
lnuA_1_M14039	232	424	AMPL3930899	Pool=2
lnuB_1_AJ238249	51	213	AMPL3930902	Pool=1
lnuB_1_AJ238249	202	344	AMPL3930903	Pool=2
lnuB_1_AJ238249	333	543	AMPL3930904	Pool=1
lnuB_1_AJ238249	532	740	AMPL3930905	Pool=2
lnuC_1_AY928180	43	259	AMPL3930906	Pool=1
lnuC_1_AY928180	248	425	AMPL3930907	Pool=2
lsaB_NG_047933	54	121	AMPL3929441	Pool=1
lsaB_NG_047933	108	301	AMPL3931455	Pool=2
lsaB_NG_047933	290	503	AMPL3931456	Pool=1
lsaB_NG_047933	491	709	AMPL3931457	Pool=2

lsaB_NG_047933	698	914	AMPL3931458	Pool=1
lsaB_NG_047933	903	1061	AMPL3931459	Pool=2
lsaB_NG_047933	1050	1222	AMPL3931460	Pool=1
lsaB_NG_047933	1211	1409	AMPL3931461	Pool=2
lsaE_JX560992	48	200	AMPL3931462	Pool=1
lsaE_JX560992	189	407	AMPL3931463	Pool=2
lsaE_JX560992	396	616	AMPL3931464	Pool=1
lsaE_JX560992	605	824	AMPL3931465	Pool=2
lsaE_JX560992	813	1028	AMPL3931466	Pool=1
lsaE_JX560992	1018	1233	AMPL3931467	Pool=2
lsaE_JX560992	1110	1285	AMPL3931468	Pool=1
mcr_1_11_1_KY853650	23	221	AMPL3931133	Pool=1
mcr_1_11_1_KY853650	210	375	AMPL3931134	Pool=2
mcr_1_11_1_KY853650	364	574	AMPL3931135	Pool=1
mcr_1_11_1_KY853650	563	783	AMPL3931136	Pool=2
mcr_1_11_1_KY853650	772	926	AMPL3931137	Pool=1
mcr_1_11_1_KY853650	915	1141	AMPL3931138	Pool=2
mcr_1_11_1_KY853650	1130	1357	AMPL3931139	Pool=1
mcr_1_11_1_KY853650	1346	1561	AMPL3931140	Pool=2
mcr_2_1_1_LT598652	26	171	AMPL3931141	Pool=1
mcr_2_1_1_LT598652	160	369	AMPL3931142	Pool=2
mcr_2_1_1_LT598652	361	579	AMPL3931143	Pool=1
mcr_2_1_1_LT598652	568	789	AMPL3931144	Pool=2
mcr_2_1_1_LT598652	769	953	AMPL3931145	Pool=1
mcr_2_1_1_LT598652	942	1129	AMPL3931146	Pool=2
mcr_2_1_1_LT598652	1118	1337	AMPL3931147	Pool=1

mcr_2_1_1_LT598652	1326	1549	AMPL3931148	Pool=2
mcr_3_17_1_MH332767	64	241	AMPL3931157	Pool=1
mcr_3_17_1_MH332767	230	451	AMPL3931158	Pool=2
mcr_3_17_1_MH332767	440	602	AMPL3931159	Pool=1
mcr_3_17_1_MH332767	595	805	AMPL3931160	Pool=2
mcr_3_17_1_MH332767	794	1015	AMPL3931161	Pool=1
mcr_3_17_1_MH332767	1004	1226	AMPL3931162	Pool=2
mcr_3_17_1_MH332767	1215	1429	AMPL3931163	Pool=1
mcr_3_17_1_MH332767	1419	1560	AMPL3931164	Pool=2
mcr_3_1_1_KY924928	50	220	AMPL3931149	Pool=1
mcr_3_1_1_KY924928	205	417	AMPL3931150	Pool=2
mcr_3_1_1_KY924928	406	598	AMPL3931151	Pool=1
mcr_3_1_1_KY924928	587	807	AMPL3931152	Pool=2
mcr_3_1_1_KY924928	796	1012	AMPL3931153	Pool=1
mcr_3_1_1_KY924928	1001	1223	AMPL3931154	Pool=2
mcr_3_1_1_KY924928	1212	1367	AMPL3931155	Pool=1
mcr_3_1_1_KY924928	1356	1560	AMPL3931156	Pool=2
mcr_4_1_1_MF543359	42	226	AMPL3931165	Pool=1
mcr_4_1_1_MF543359	215	405	AMPL3931166	Pool=2
mcr_4_1_1_MF543359	394	584	AMPL3931167	Pool=1
mcr_4_1_1_MF543359	573	794	AMPL3931168	Pool=2
mcr_4_1_1_MF543359	783	998	AMPL3931169	Pool=1
mcr_4_1_1_MF543359	987	1207	AMPL3931170	Pool=2
mcr_4_1_1_MF543359	1196	1350	AMPL3931171	Pool=1
mcr_4_1_1_MF543359	1339	1556	AMPL3931172	Pool=2
mcr_5_1_1_KY807921	63	280	AMPL3931173	Pool=1

mcr_5_1_1_KY807921	269	426	AMPL3931174	Pool=2
mcr_5_1_1_KY807921	415	614	AMPL3931175	Pool=1
mcr_5_1_1_KY807921	603	814	AMPL3931176	Pool=2
mcr_5_1_1_KY807921	845	980	AMPL3931178	Pool=1
mcr_5_1_1_KY807921	969	1106	AMPL3931179	Pool=2
mcr_5_1_1_KY807921	1095	1285	AMPL3931180	Pool=1
mcr_5_1_1_KY807921	1274	1476	AMPL3931181	Pool=2
mcr_5_1_1_KY807921	1465	1609	AMPL3931182	Pool=1
mcr_6_1_1_MF176240	22	211	AMPL3931183	Pool=1
mcr_6_1_1_MF176240	200	424	AMPL3931184	Pool=2
mcr_6_1_1_MF176240	413	639	AMPL3931185	Pool=1
mcr_6_1_1_MF176240	628	851	AMPL3931186	Pool=2
mcr_6_1_1_MF176240	837	1019	AMPL3931187	Pool=1
mcr_6_1_1_MF176240	1008	1149	AMPL3931188	Pool=2
mcr_6_1_1_MF176240	1138	1338	AMPL3931189	Pool=1
mcr_6_1_1_MF176240	1327	1550	AMPL3931190	Pool=2
mcr_7_1_1_MG267386	55	283	AMPL3931191	Pool=1
mcr_7_1_1_MG267386	272	498	AMPL3931192	Pool=2
mcr_7_1_1_MG267386	437	568	AMPL3931193	Pool=1
mcr_7_1_1_MG267386	697	783	AMPL3928928	Pool=2
mcr_7_1_1_MG267386	759	931	AMPL3931195	Pool=1
mcr_7_1_1_MG267386	883	1051	AMPL3931196	Pool=2
mcr_7_1_1_MG267386	1040	1256	AMPL3931197	Pool=1
mcr_7_1_1_MG267386	1245	1324	AMPL3931198	Pool=2
mcr_7_1_1_MG267386	1307	1501	AMPL3931199	Pool=1
mcr_8_1_MG736312	42	220	AMPL3931201	Pool=1

mcr_8_1_MG736312	209	420	AMPL3931202	Pool=2
mcr_8_1_MG736312	409	627	AMPL3931203	Pool=1
mcr_8_1_MG736312	616	842	AMPL3931204	Pool=2
mcr_8_1_MG736312	831	1046	AMPL3931205	Pool=1
mcr_8_1_MG736312	1028	1231	AMPL3931206	Pool=2
mcr_8_1_MG736312	1220	1435	AMPL3931207	Pool=1
mcr_8_1_MG736312	1424	1633	AMPL3931208	Pool=2
mdetl1_AJ012115	49	146	AMPL3929969	Pool=1
mdetl1_AJ012115	135	307	AMPL3931737	Pool=2
mdetl1_AJ012115	296	372	AMPL3931738	Pool=1
mdetl1_AJ012115	361	579	AMPL3931739	Pool=2
mdetl1_AJ012115	568	766	AMPL3931740	Pool=1
mdetl1_AJ012115	755	959	AMPL3931741	Pool=2
mdetl1_AJ012115	948	1128	AMPL3931742	Pool=1
mdtE_NC_000913	42	263	AMPL3931743	Pool=1
mdtE_NC_000913	254	477	AMPL3931744	Pool=2
mdtE_NC_000913	461	685	AMPL3931745	Pool=1
mdtE_NC_000913	674	893	AMPL3931746	Pool=2
mdtE_NC_000913	882	1111	AMPL3931747	Pool=1
mecA_10_AB512767	60	221	AMPL3930819	Pool=1
mecA_10_AB512767	209	354	AMPL3930820	Pool=2
mecA_10_AB512767	343	554	AMPL3930821	Pool=1
mecA_10_AB512767	543	759	AMPL3930822	Pool=2
mecA_10_AB512767	748	860	AMPL3930823	Pool=1
mecA_10_AB512767	849	1064	AMPL3930824	Pool=2
mecA_10_AB512767	1053	1269	AMPL3930825	Pool=1

mecA_10_AB512767	1257	1453	AMPL3930826	Pool=2
mecA_10_AB512767	1442	1610	AMPL3930827	Pool=1
mecA_10_AB512767	1599	1812	AMPL3930828	Pool=2
mecA_10_AB512767	1801	1953	AMPL3930829	Pool=1
mefA_3_AF227521	66	159	AMPL3930908	Pool=1
mefA_3_AF227521	148	369	AMPL3930909	Pool=2
mefA_3_AF227521	358	551	AMPL3930910	Pool=1
mefA_3_AF227521	540	755	AMPL3930911	Pool=2
mefA_3_AF227521	744	962	AMPL3930912	Pool=1
mefA_3_AF227521	951	1151	AMPL3930913	Pool=2
mefB_1_FJ196385	59	168	AMPL3930914	Pool=1
mefB_1_FJ196385	157	385	AMPL3930915	Pool=2
mefB_1_FJ196385	372	572	AMPL3930916	Pool=1
mefB_1_FJ196385	561	785	AMPL3930917	Pool=2
mefB_1_FJ196385	774	993	AMPL3930918	Pool=1
mefB_1_FJ196385	982	1165	AMPL3930919	Pool=2
mel_KU984333	38	198	AMPL3931569	Pool=1
mel_KU984333	187	410	AMPL3931570	Pool=2
mel_KU984333	399	590	AMPL3931571	Pool=1
mel_KU984333	579	799	AMPL3931572	Pool=2
mel_KU984333	788	1007	AMPL3931573	Pool=1
mel_KU984333	996	1221	AMPL3931574	Pool=2
mel_KU984333	1210	1425	AMPL3931575	Pool=1
mepA_AY661734	52	173	AMPL3931748	Pool=1
mepA_AY661734	162	335	AMPL3931749	Pool=2
mepA_AY661734	324	501	AMPL3931750	Pool=1

mepA_AY661734	488	710	AMPL3931751	Pool=2
mepA_AY661734	696	908	AMPL3931752	Pool=1
mepA_AY661734	897	1105	AMPL3931753	Pool=2
mepA_AY661734	1093	1305	AMPL3931754	Pool=1
mphA_1_D16251	53	182	AMPL3928402	Pool=1
mphA_1_D16251	171	325	AMPL3930920	Pool=2
mphA_1_D16251	314	538	AMPL3930921	Pool=1
mphA_1_D16251	468	679	AMPL3930922	Pool=2
mphA_1_D16251	635	861	AMPL3930923	Pool=1
mphB_1_D85892	69	220	AMPL3930924	Pool=2
mphB_1_D85892	209	431	AMPL3930925	Pool=1
mphB_1_D85892	420	627	AMPL3930926	Pool=2
mphB_1_D85892	616	839	AMPL3930927	Pool=1
mphC_1_AB013298	60	157	AMPL3930928	Pool=2
mphC_1_AB013298	146	365	AMPL3930929	Pool=1
mphC_1_AB013298	354	544	AMPL3930930	Pool=2
mphC_1_AB013298	533	628	AMPL3928424	Pool=1
mphC_1_AB013298	617	830	AMPL3930931	Pool=2
msrA_1_X52085	67	196	AMPL3930932	Pool=1
msrA_1_X52085	185	292	AMPL3930933	Pool=2
msrA_1_X52085	281	502	AMPL3930934	Pool=1
msrA_1_X52085	491	714	AMPL3930935	Pool=2
msrA_1_X52085	703	927	AMPL3930936	Pool=1
msrA_1_X52085	920	1070	AMPL3930937	Pool=2
msrA_1_X52085	1059	1275	AMPL3930938	Pool=1
msrA_1_X52085	1264	1423	AMPL3930939	Pool=2

msrC_1_AY004350	50	193	AMPL3930940	Pool=1
msrC_1_AY004350	182	403	AMPL3930941	Pool=2
msrC_1_AY004350	392	594	AMPL3930942	Pool=1
msrC_1_AY004350	583	803	AMPL3930943	Pool=2
msrC_1_AY004350	792	1004	AMPL3930944	Pool=1
msrC_1_AY004350	993	1211	AMPL3930945	Pool=2
msrC_1_AY004350	1200	1411	AMPL3930946	Pool=1
msrD_2_AF274302	42	170	AMPL3930947	Pool=1
msrD_2_AF274302	159	382	AMPL3930948	Pool=2
msrD_2_AF274302	371	591	AMPL3930949	Pool=1
msrD_2_AF274302	580	804	AMPL3930950	Pool=2
msrD_2_AF274302	793	1015	AMPL3930951	Pool=1
msrD_2_AF274302	1004	1225	AMPL3930952	Pool=2
msrD_2_AF274302	1214	1394	AMPL3930953	Pool=1
mtrC_HQ712081	30	246	AMPL3931755	Pool=1
mtrC_HQ712081	232	460	AMPL3931756	Pool=2
mtrC_HQ712081	449	670	AMPL3931757	Pool=1
mtrC_HQ712081	658	877	AMPL3931758	Pool=2
mtrC_HQ712081	867	1087	AMPL3931759	Pool=1
mtrC_HQ712081	1076	1266	AMPL3931760	Pool=2
mtrD_HQ706341	18	233	AMPL3931761	Pool=1
nimE_NG_048015	46	274	AMPL3931763	Pool=2
nimE_NG_048015	263	479	AMPL3931764	Pool=1
nisB_X68307	35	169	AMPL3931765	Pool=2
nisB_X68307	157	346	AMPL3931766	Pool=1
nisB_X68307	335	445	AMPL3931767	Pool=2

nisB_X68307	404	616	AMPL3931768	Pool=1
nisB_X68307	605	809	AMPL3931769	Pool=2
nisB_X68307	717	932	AMPL3931770	Pool=1
nisB_X68307	877	1068	AMPL3931771	Pool=2
nisB_X68307	985	1175	AMPL3931772	Pool=1
nisB_X68307	1164	1389	AMPL3931773	Pool=2
nisB_X68307	1378	1569	AMPL3931774	Pool=1
nisB_X68307	1558	1744	AMPL3931775	Pool=2
nisB_X68307	1651	1829	AMPL3931776	Pool=1
nisB_X68307	1797	1955	AMPL3931777	Pool=2
nisB_X68307	1927	2118	AMPL3931778	Pool=1
nisB_X68307	2107	2327	AMPL3931779	Pool=2
nisB_X68307	2316	2479	AMPL3931780	Pool=1
nisB_X68307	2443	2575	AMPL3931781	Pool=2
nisB_X68307	2552	2731	AMPL3931782	Pool=1
nisB_X68307	2652	2867	AMPL3931783	Pool=2
nisB_X68307	2856	2931	AMPL3931784	Pool=1
npmA_1_AB261016	29	197	AMPL3931015	Pool=1
npmA_1_AB261016	186	388	AMPL3931016	Pool=2
npmA_1_AB261016	377	592	AMPL3931017	Pool=1
oleC_1_L06249	30	183	AMPL3930954	Pool=2
oleC_1_L06249	151	274	AMPL3930955	Pool=1
oleC_1_L06249	263	501	AMPL3930956	Pool=2
oleC_1_L06249	470	593	AMPL3930957	Pool=1
oleC_1_L06249	575	801	AMPL3930958	Pool=2
oleC_1_L06249	777	959	AMPL3930959	Pool=1

optrA_NG_048023_1	64	282	AMPL3931966	Pool=1
optrA_NG_048023_1	269	479	AMPL3931967	Pool=2
optrA_NG_048023_1	468	593	AMPL3931968	Pool=1
optrA_NG_048023_1	582	778	AMPL3931969	Pool=2
optrA_NG_048023_1	758	823	AMPL3931970	Pool=1
optrA_NG_048023_1	994	1162	AMPL3931972	Pool=2
oqxA_1_EU370913	66	283	AMPL3931060	Pool=1
oqxA_1_EU370913	272	389	AMPL3931061	Pool=2
oqxA_1_EU370913	403	632	AMPL3931063	Pool=1
oqxA_1_EU370913	529	666	AMPL3931064	Pool=2
oqxA_1_EU370913	823	980	AMPL3931066	Pool=1
oqxA_1_EU370913	969	1121	AMPL3931067	Pool=2
oqxB_1_EU370913	40	231	AMPL3931068	Pool=1
oqxB_1_EU370913	227	446	AMPL3931069	Pool=2
oqxB_1_EU370913	435	571	AMPL3931070	Pool=1
oqxB_1_EU370913	557	725	AMPL3931071	Pool=2
oqxB_1_EU370913	714	899	AMPL3931072	Pool=1
oqxB_1_EU370913	891	1100	AMPL3931073	Pool=2
oqxB_1_EU370913	1085	1249	AMPL3931074	Pool=1
oqxB_1_EU370913	1223	1441	AMPL3931075	Pool=2
oqxB_1_EU370913	1417	1601	AMPL3931076	Pool=1
oqxB_1_EU370913	1587	1799	AMPL3931077	Pool=2
oqxB_1_EU370913	1730	1948	AMPL3931078	Pool=1
oqxB_1_EU370913	1910	2022	AMPL3931079	Pool=2
oqxB_1_EU370913	1988	2152	AMPL3931080	Pool=1
oqxB_1_EU370913	2073	2265	AMPL3931081	Pool=2

oqxB_1_EU370913	2253	2388	AMPL3931082	Pool=1
oqxB_1_EU370913	2377	2579	AMPL3931083	Pool=2
oqxB_1_EU370913	2568	2780	AMPL3931084	Pool=1
oqxB_1_EU370913	2747	2941	AMPL3931085	Pool=2
oqxB_1_EU370913	2862	3085	AMPL3931086	Pool=1
pbp1a_AF446215	31	139	AMPL3931854	Pool=1
pbp1a_AF446215	128	352	AMPL3931855	Pool=2
pbp1a_AF446215	341	539	AMPL3931856	Pool=1
pbp1a_AF446215	528	731	AMPL3931857	Pool=2
pbp1a_AF446215	720	938	AMPL3931858	Pool=1
pbp1a_AF446215	927	1139	AMPL3931859	Pool=2
pbp1a_AF446215	1128	1349	AMPL3931860	Pool=1
pbp1a_AF446215	1338	1555	AMPL3931861	Pool=2
pbp1a_AF446215	1544	1723	AMPL3931862	Pool=1
pbp2a_AF101780	70	167	AMPL3930257	Pool=1
pbp2a_AF101780	156	377	AMPL3931886	Pool=2
pbp2a_AF101780	366	583	AMPL3931887	Pool=1
pbp2a_AF101780	572	790	AMPL3931888	Pool=2
pbp2a_AF101780	779	965	AMPL3931889	Pool=1
pbp2a_AF101780	954	1181	AMPL3931890	Pool=2
pbp2a_AF101780	1170	1343	AMPL3931891	Pool=1
pbp2a_AF101780	1332	1553	AMPL3931892	Pool=2
pbp2a_AF101780	1542	1747	AMPL3931893	Pool=1
pbp2a_AF101780	1736	1937	AMPL3931894	Pool=2
pbp2a_AF101780	1926	2145	AMPL3931895	Pool=1
pbp3_AJ243120	89	218	AMPL3931863	Pool=2

pbp3_AJ243120	207	403	AMPL3931864	Pool=1
pbp3_AJ243120	392	599	AMPL3931865	Pool=2
pbp3_AJ243120	588	667	AMPL3931866	Pool=1
pbp3_AJ243120	656	874	AMPL3931867	Pool=2
pbp3_AJ243120	863	1054	AMPL3931868	Pool=1
pbp3_AJ243120	1043	1255	AMPL3931869	Pool=2
pbp3_AJ243120	1244	1456	AMPL3931870	Pool=1
pbp3_AJ243120	1445	1667	AMPL3931871	Pool=2
pbp3_AJ243120	1656	1824	AMPL3931872	Pool=1
pbp3_AJ243120	1813	2009	AMPL3931873	Pool=2
pbp5_AF375986	64	260	AMPL3931877	Pool=1
pbp5_AF375986	249	443	AMPL3931878	Pool=2
pbp5_AF375986	432	618	AMPL3931879	Pool=1
pexA_NG_048035	24	171	AMPL3931087	Pool=1
pexA_NG_048035	160	360	AMPL3931088	Pool=2
pexA_NG_048035	349	561	AMPL3931089	Pool=1
pexA_NG_048035	550	752	AMPL3931090	Pool=2
pexA_NG_048035	741	967	AMPL3931091	Pool=1
pexA_NG_048035	956	1183	AMPL3931092	Pool=2
pexA_NG_048035	1172	1397	AMPL3931093	Pool=1
pikR1_AF079138	21	186	AMPL3931556	Pool=2
pikR1_AF079138	179	399	AMPL3931557	Pool=1
pikR1_AF079138	385	605	AMPL3931558	Pool=2
pikR1_AF079138	567	759	AMPL3931559	Pool=1
pikR1_AF079138	748	976	AMPL3931560	Pool=2
pikR2_AF079138	44	229	AMPL3931561	Pool=1

pikR2_AF079138	71	308	AMPL3931562	Pool=2
pikR2_AF079138	266	452	AMPL3931563	Pool=1
pikR2_AF079138	534	768	AMPL3931565	Pool=2
pikR2_AF079138	755	905	AMPL3931566	Pool=1
pncA_KY659393	32	185	AMPL3931785	Pool=1
pncA_KY659393	154	346	AMPL3931786	Pool=2
pncA_KY659393	335	538	AMPL3931787	Pool=1
qacA_NG_061385	74	255	AMPL3931789	Pool=2
qacA_NG_061385	244	448	AMPL3931790	Pool=1
qacA_NG_061385	437	639	AMPL3931791	Pool=2
qacA_NG_061385	628	818	AMPL3931792	Pool=1
qacA_NG_061385	807	935	AMPL3931793	Pool=2
qacA_NG_061385	924	1145	AMPL3931794	Pool=1
qacA_NG_061385	1134	1345	AMPL3931795	Pool=2
qacA_NG_061385	1334	1494	AMPL3931796	Pool=1
qacE_FJ663011	67	285	AMPL3931799	Pool=2
qacH_AY816216	25	200	AMPL3931800	Pool=1
qacH_AY816216	189	273	AMPL3931801	Pool=2
qac_U81980	52	138	AMPL3930070	Pool=1
qac_U81980	127	283	AMPL3931788	Pool=2
qepA_1_AB263754	20	252	AMPL3931094	Pool=1
qepA_1_AB263754	110	282	AMPL3931095	Pool=2
qepA_1_AB263754	432	653	AMPL3931097	Pool=1
qepA_1_AB263754	642	863	AMPL3931098	Pool=2
qepA_1_AB263754	857	1013	AMPL3931099	Pool=1
qepA_1_AB263754	997	1116	AMPL3931100	Pool=2

qepA_1_AB263754	1130	1330	AMPL3931102	Pool=1
qepA_1_AB263754	1319	1488	AMPL3931103	Pool=2
rarD_L02122	52	282	AMPL3931802	Pool=1
rarD_L02122	271	439	AMPL3931803	Pool=2
rarD_L02122	428	657	AMPL3931804	Pool=1
rarD_L02122	646	840	AMPL3931805	Pool=2
rmtA_1_AB120321	40	266	AMPL3931018	Pool=1
rmtA_1_AB120321	229	312	AMPL3931019	Pool=2
rmtA_1_AB120321	482	686	AMPL3931021	Pool=1
rmtB2_1_JN968578	42	251	AMPL3931022	Pool=1
rmtB2_1_JN968578	243	452	AMPL3931023	Pool=2
rmtB2_1_JN968578	447	598	AMPL3931024	Pool=1
rmtB2_1_JN968578	587	686	AMPL3931025	Pool=2
rmtC_1_AB194779	49	226	AMPL3931026	Pool=1
rmtC_1_AB194779	215	420	AMPL3931027	Pool=2
rmtC_1_AB194779	409	611	AMPL3931028	Pool=1
rmtC_1_AB194779	600	776	AMPL3931029	Pool=2
rmtD_1_DQ914960	24	174	AMPL3931030	Pool=1
rmtD_1_DQ914960	102	279	AMPL3931031	Pool=2
rmtD_1_DQ914960	300	452	AMPL3931033	Pool=1
rmtD_1_DQ914960	436	632	AMPL3931034	Pool=2
rmtD_1_DQ914960	601	707	AMPL3931035	Pool=1
rmtE_1_GU201947	30	175	AMPL3931036	Pool=1
rmtE_1_GU201947	164	363	AMPL3931037	Pool=2
rmtE_1_GU201947	352	572	AMPL3931038	Pool=1
rmtE_1_GU201947	564	777	AMPL3931039	Pool=2

rmtG_1_KJ004567	51	267	AMPL3931044	Pool=1
rmtG_1_KJ004567	242	384	AMPL3931045	Pool=2
rmtG_1_KJ004567	373	514	AMPL3931046	Pool=1
rmtG_1_KJ004567	512	725	AMPL3931047	Pool=2
rmtH_1_KC544262	40	181	AMPL3931048	Pool=1
rmtH_1_KC544262	170	316	AMPL3931049	Pool=2
rmtH_1_KC544262	305	530	AMPL3931050	Pool=1
rmtH_1_KC544262	519	689	AMPL3931051	Pool=2
rmtf_1_JQ808129	63	295	AMPL3931040	Pool=1
rmtf_1_JQ808129	239	437	AMPL3931041	Pool=2
rmtf_1_JQ808129	426	536	AMPL3931042	Pool=1
rmtf_1_JQ808129	514	730	AMPL3931043	Pool=2
sat4_AB699882	22	123	AMPL3931554	Pool=1
sat4_AB699882	112	328	AMPL3931555	Pool=2
sat4_AB699882	317	416	AMPL3929630	Pool=1
sdeB_CP033162	32	242	AMPL3931806	Pool=1
sdeB_CP033162	200	400	AMPL3931807	Pool=2
sdeB_CP033162	353	575	AMPL3931808	Pool=1
sdeB_CP033162	564	735	AMPL3931809	Pool=2
sdeB_CP033162	628	857	AMPL3931810	Pool=1
sdeB_CP033162	857	1068	AMPL3931811	Pool=2
sdeB_CP033162	1043	1258	AMPL3931812	Pool=1
sdeB_CP033162	1246	1399	AMPL3931813	Pool=2
sdeB_CP033162	1311	1535	AMPL3931814	Pool=1
sdeB_CP033162	1501	1726	AMPL3931815	Pool=2
sdeB_CP033162	1720	1946	AMPL3931816	Pool=1

sdeB_CP033162	1899	2125	AMPL3931817	Pool=2
sdeB_CP033162	2115	2256	AMPL3931818	Pool=1
sdeB_CP033162	2245	2430	AMPL3931819	Pool=2
sdeB_CP033162	2414	2601	AMPL3931820	Pool=1
sdeB_CP033162	2581	2737	AMPL3931821	Pool=2
sdeB_CP033162	2704	2914	AMPL3931822	Pool=1
sdeB_CP033162	2913	3104	AMPL3931823	Pool=2
spcN_AF170704	25	169	AMPL3932004	Pool=1
spcN_AF170704	160	333	AMPL3932005	Pool=2
spcN_AF170704	629	721	AMPL3932006	Pool=1
spcN_AF170704	710	941	AMPL3932007	Pool=2
speA_KY594413	64	231	AMPL3931883	Pool=1
speA_KY594413	220	435	AMPL3931884	Pool=2
speA_KY594413	416	615	AMPL3931885	Pool=1
strA_1_M96392	44	271	AMPL3931052	Pool=1
strA_1_M96392	256	468	AMPL3931053	Pool=2
strA_1_M96392	457	563	AMPL3928669	Pool=1
strA_1_M96392	552	762	AMPL3931054	Pool=2
strB_EF493834	33	220	AMPL3931055	Pool=1
strB_EF493834	209	334	AMPL3931056	Pool=2
strB_EF493834	323	521	AMPL3931057	Pool=1
strB_EF493834	506	719	AMPL3931058	Pool=2
strB_EF493834	705	852	AMPL3931059	Pool=1
str_X92946	58	212	AMPL3931525	Pool=1
str_X92946	190	394	AMPL3931526	Pool=2
str_X92946	428	577	AMPL3931528	Pool=1

str_X92946	534	713	AMPL3931529	Pool=2
sul1_EF667294	29	219	AMPL3931667	Pool=1
sul1_EF667294	197	364	AMPL3931668	Pool=2
sul1_EF667294	351	571	AMPL3931669	Pool=1
sul1_EF667294	457	660	AMPL3931670	Pool=2
sul1_EF667294	646	791	AMPL3931671	Pool=1
sul2_DQ464881	28	223	AMPL3931672	Pool=2
sul2_DQ464881	204	432	AMPL3931673	Pool=1
sul2_DQ464881	398	616	AMPL3931674	Pool=2
sul2_DQ464881	507	727	AMPL3931675	Pool=1
sulA_KJ831060	29	168	AMPL3932023	Pool=1
sulA_KJ831060	157	367	AMPL3932024	Pool=2
sulA_KJ831060	356	576	AMPL3932025	Pool=1
sulA_KJ831060	565	782	AMPL3932026	Pool=2
sulA_KJ831060	771	902	AMPL3932027	Pool=1
tet32_1_EU722333	59	280	AMPL3930531	Pool=2
tet32_1_EU722333	269	482	AMPL3930532	Pool=1
tet32_1_EU722333	467	657	AMPL3930533	Pool=2
tet32_1_EU722333	646	868	AMPL3930534	Pool=1
tet32_1_EU722333	856	1066	AMPL3930535	Pool=2
tet32_1_EU722333	1052	1278	AMPL3930536	Pool=1
tet32_1_EU722333	1267	1478	AMPL3930537	Pool=2
tet32_1_EU722333	1467	1661	AMPL3930538	Pool=1
tet32_1_EU722333	1650	1872	AMPL3930539	Pool=2
tet34_1_AB061440	44	178	AMPL3930540	Pool=1
tet34_1_AB061440	167	395	AMPL3930541	Pool=2

tet35_1_AF353562	25	218	AMPL3930542	Pool=1
tet35_1_AF353562	207	431	AMPL3930543	Pool=2
tet35_1_AF353562	420	618	AMPL3930544	Pool=1
tet35_1_AF353562	607	832	AMPL3930545	Pool=2
tet35_1_AF353562	821	1040	AMPL3930546	Pool=1
tet36_1_AJ514254	45	254	AMPL3930547	Pool=1
tet36_1_AJ514254	243	450	AMPL3930548	Pool=2
tet36_1_AJ514254	424	601	AMPL3930549	Pool=1
tet36_1_AJ514254	590	769	AMPL3930550	Pool=2
tet36_1_AJ514254	758	942	AMPL3930551	Pool=1
tet36_1_AJ514254	931	1149	AMPL3930552	Pool=2
tet36_1_AJ514254	1138	1265	AMPL3930553	Pool=1
tet36_1_AJ514254	1254	1454	AMPL3930554	Pool=2
tet36_1_AJ514254	1443	1665	AMPL3930555	Pool=1
tet36_1_AJ514254	1654	1854	AMPL3930556	Pool=2
tet37_1_AF540889	54	273	AMPL3930557	Pool=1
tetAP_1_AB054980	66	173	AMPL3927682	Pool=2
tetAP_1_AB054980	162	352	AMPL3930558	Pool=1
tetAP_1_AB054980	341	541	AMPL3930559	Pool=2
tetAP_1_AB054980	503	690	AMPL3930560	Pool=1
tetAP_1_AB054980	720	806	AMPL3927698	Pool=2
tetAP_1_AB054980	795	988	AMPL3930562	Pool=1
tetAP_1_AB054980	1017	1207	AMPL3930564	Pool=2
tetA_2_X00006	39	249	AMPL3930577	Pool=1
tetA_2_X00006	169	401	AMPL3930578	Pool=2
tetA_2_X00006	383	533	AMPL3930579	Pool=1

tetA_2_X00006	524	660	AMPL3930580	Pool=2
tetA_2_X00006	660	869	AMPL3930581	Pool=1
tetA_2_X00006	857	1084	AMPL3930582	Pool=2
tetA_2_X00006	1073	1182	AMPL3927738	Pool=1
tetBP_3_NC_010937	53	260	AMPL3930565	Pool=1
tetBP_3_NC_010937	252	467	AMPL3930566	Pool=2
tetBP_3_NC_010937	433	630	AMPL3930567	Pool=1
tetBP_3_NC_010937	580	735	AMPL3930568	Pool=2
tetBP_3_NC_010937	715	928	AMPL3930569	Pool=1
tetBP_3_NC_010937	958	1049	AMPL3930571	Pool=2
tetBP_3_NC_010937	1025	1184	AMPL3930572	Pool=1
tetBP_3_NC_010937	1173	1327	AMPL3930573	Pool=2
tetBP_3_NC_010937	1316	1471	AMPL3930574	Pool=1
tetBP_3_NC_010937	1460	1655	AMPL3930575	Pool=2
tetBP_3_NC_010937	1644	1842	AMPL3930576	Pool=1
tetBP_3_NC_010937	1831	1927	AMPL3927725	Pool=2
tetB_3_AP000342	27	246	AMPL3930583	Pool=1
tetB_3_AP000342	235	437	AMPL3930584	Pool=2
tetB_3_AP000342	426	554	AMPL3930585	Pool=1
tetB_3_AP000342	543	754	AMPL3930586	Pool=2
tetB_3_AP000342	743	965	AMPL3930587	Pool=1
tetB_3_AP000342	954	1136	AMPL3930588	Pool=2
tetC_2_NC_003123	28	149	AMPL3927750	Pool=1
tetC_2_NC_003123	122	351	AMPL3930589	Pool=2
tetC_2_NC_003123	254	467	AMPL3930590	Pool=1
tetC_2_NC_003123	431	656	AMPL3930591	Pool=2

tetC_2_NC_003123	645	785	AMPL3930592	Pool=1
tetC_2_NC_003123	774	1001	AMPL3930593	Pool=2
tetC_2_NC_003123	990	1127	AMPL3930594	Pool=1
tetD_1_AF467077	70	300	AMPL3930597	Pool=1
tetD_1_AF467077	290	478	AMPL3930598	Pool=2
tetD_1_AF467077	530	678	AMPL3930600	Pool=1
tetD_1_AF467077	647	855	AMPL3930601	Pool=2
tetD_1_AF467077	833	978	AMPL3930602	Pool=1
tetD_1_AF467077	965	1132	AMPL3930603	Pool=2
tetE_3_L06940	32	241	AMPL3930604	Pool=1
tetE_3_L06940	230	390	AMPL3930605	Pool=2
tetE_3_L06940	379	591	AMPL3930606	Pool=1
tetE_3_L06940	580	798	AMPL3930607	Pool=2
tetE_3_L06940	787	954	AMPL3930608	Pool=1
tetE_3_L06940	943	1148	AMPL3930609	Pool=2
tetG_3_S52437	32	144	AMPL3930610	Pool=1
tetG_3_S52437	133	335	AMPL3930611	Pool=2
tetG_3_S52437	299	520	AMPL3930612	Pool=1
tetG_3_S52437	475	687	AMPL3930613	Pool=2
tetG_3_S52437	670	893	AMPL3930614	Pool=1
tetG_3_S52437	882	997	AMPL3927797	Pool=2
tetG_3_S52437	986	1126	AMPL3930615	Pool=1
tetH_1_Y16103	49	275	AMPL3930616	Pool=1
tetH_1_Y16103	264	484	AMPL3930617	Pool=2
tetH_1_Y16103	473	690	AMPL3930618	Pool=1
tetH_1_Y16103	679	894	AMPL3930619	Pool=2

tetH_1_Y16103	883	977	AMPL3927808	Pool=1
tetH_1_Y16103	966	1133	AMPL3930620	Pool=2
tetJ_1_ACLE01000065	47	265	AMPL3930621	Pool=1
tetJ_1_ACLE01000065	254	447	AMPL3930622	Pool=2
tetJ_1_ACLE01000065	435	620	AMPL3930623	Pool=1
tetJ_1_ACLE01000065	606	829	AMPL3930624	Pool=2
tetJ_1_ACLE01000065	818	968	AMPL3930625	Pool=1
tetJ_1_ACLE01000065	957	1129	AMPL3930626	Pool=2
tetK_4_U38428	56	173	AMPL3930627	Pool=1
tetK_4_U38428	162	355	AMPL3930628	Pool=2
tetK_4_U38428	344	549	AMPL3930629	Pool=1
tetK_4_U38428	531	719	AMPL3930630	Pool=2
tetK_4_U38428	774	887	AMPL3930632	Pool=1
tetK_4_U38428	876	1024	AMPL3930633	Pool=2
tetK_4_U38428	942	1151	AMPL3930634	Pool=1
tetK_4_U38428	1149	1348	AMPL3930635	Pool=2
tetL_1_HM235948	37	179	AMPL3930636	Pool=1
tetL_1_HM235948	168	304	AMPL3930637	Pool=2
tetL_1_HM235948	293	511	AMPL3930638	Pool=1
tetL_1_HM235948	500	718	AMPL3930639	Pool=2
tetL_1_HM235948	704	906	AMPL3930640	Pool=1
tetL_1_HM235948	895	1116	AMPL3930641	Pool=2
tetL_1_HM235948	1105	1313	AMPL3930642	Pool=1
tetM_1_X92947	42	125	AMPL3927857	Pool=1
tetM_1_X92947	114	315	AMPL3930643	Pool=2
tetM_1_X92947	304	432	AMPL3930644	Pool=1

tetM_1_X92947	421	616	AMPL3930645	Pool=2
tetM_1_X92947	605	824	AMPL3930646	Pool=1
tetM_1_X92947	813	1011	AMPL3930647	Pool=2
tetM_1_X92947	1000	1072	AMPL3930648	Pool=1
tetM_1_X92947	1061	1280	AMPL3930649	Pool=2
tetM_1_X92947	1269	1453	AMPL3930650	Pool=1
tetM_1_X92947	1442	1659	AMPL3930651	Pool=2
tetM_1_X92947	1648	1865	AMPL3930652	Pool=1
tetO_1_M18896	50	250	AMPL3930653	Pool=1
tetO_1_M18896	239	436	AMPL3930654	Pool=2
tetO_1_M18896	425	607	AMPL3930655	Pool=1
tetO_1_M18896	596	816	AMPL3930656	Pool=2
tetO_1_M18896	808	1020	AMPL3930657	Pool=1
tetO_1_M18896	1009	1206	AMPL3930658	Pool=2
tetO_1_M18896	1195	1401	AMPL3930659	Pool=1
tetO_1_M18896	1390	1595	AMPL3930660	Pool=2
tetO_1_M18896	1584	1660	AMPL3927892	Pool=1
tetO_1_M18896	1649	1871	AMPL3930661	Pool=2
tetQ_3_U73497	22	154	AMPL3930662	Pool=1
tetQ_3_U73497	143	360	AMPL3930663	Pool=2
tetQ_3_U73497	349	571	AMPL3930664	Pool=1
tetQ_3_U73497	560	773	AMPL3930665	Pool=2
tetQ_3_U73497	762	985	AMPL3930666	Pool=1
tetQ_3_U73497	974	1044	AMPL3927905	Pool=2
tetQ_3_U73497	1227	1441	AMPL3930668	Pool=1
tetQ_3_U73497	1430	1579	AMPL3930669	Pool=2

tetQ_3_U73497	1568	1771	AMPL3930670	Pool=1
tetQ_3_U73497	1760	1952	AMPL3930671	Pool=2
tetR_KF697108	31	190	AMPL3930672	Pool=1
tetR_KF697108	179	396	AMPL3930673	Pool=2
tetR_KF697108	385	580	AMPL3930674	Pool=1
tetS_3_X92946	56	129	AMPL3930675	Pool=1
tetS_3_X92946	118	308	AMPL3930676	Pool=2
tetS_3_X92946	297	519	AMPL3930677	Pool=1
tetS_3_X92946	508	715	AMPL3930678	Pool=2
tetS_3_X92946	696	900	AMPL3930679	Pool=1
tetS_3_X92946	876	1078	AMPL3930680	Pool=2
tetS_3_X92946	1067	1190	AMPL3930681	Pool=1
tetS_3_X92946	1179	1358	AMPL3930682	Pool=2
tetS_3_X92946	1347	1574	AMPL3930683	Pool=1
tetS_3_X92946	1563	1785	AMPL3930684	Pool=2
tetS_3_X92946	1774	1873	AMPL3927940	Pool=1
tetT_1_L42544	46	123	AMPL3927941	Pool=1
tetT_1_L42544	112	292	AMPL3930685	Pool=2
tetT_1_L42544	281	487	AMPL3930686	Pool=1
tetT_1_L42544	415	586	AMPL3930687	Pool=2
tetT_1_L42544	721	914	AMPL3930689	Pool=1
tetT_1_L42544	903	1116	AMPL3930690	Pool=2
tetT_1_L42544	1105	1322	AMPL3930691	Pool=1
tetT_1_L42544	1311	1480	AMPL3930692	Pool=2
tetT_1_L42544	1469	1685	AMPL3930693	Pool=1
tetT_1_L42544	1674	1889	AMPL3930694	Pool=2

tetU_1_U01917	57	268	AMPL3930695	Pool=1
tetV_1_AF030344	62	279	AMPL3930696	Pool=1
tetV_1_AF030344	272	508	AMPL3930697	Pool=2
tetV_1_AF030344	491	674	AMPL3930698	Pool=1
tetV_1_AF030344	663	830	AMPL3930699	Pool=2
tetV_1_AF030344	819	930	AMPL3930700	Pool=1
tetV_1_AF030344	911	1100	AMPL3930701	Pool=2
tetV_1_AF030344	1089	1223	AMPL3930702	Pool=1
tetW_1_DQ060146	39	158	AMPL3927986	Pool=2
tetW_1_DQ060146	147	353	AMPL3930703	Pool=1
tetW_1_DQ060146	342	555	AMPL3930704	Pool=2
tetW_1_DQ060146	544	677	AMPL3930705	Pool=1
tetW_1_DQ060146	669	900	AMPL3930706	Pool=2
tetW_1_DQ060146	891	1118	AMPL3930707	Pool=1
tetW_1_DQ060146	1111	1319	AMPL3930708	Pool=2
tetW_1_DQ060146	1305	1504	AMPL3930709	Pool=1
tetW_1_DQ060146	1493	1676	AMPL3930710	Pool=2
tetW_1_DQ060146	1665	1881	AMPL3930711	Pool=1
tetX	507	678	AMPL3931973	Pool=1
tetX	667	840	AMPL3931974	Pool=2
tetX	829	1044	AMPL3931975	Pool=1
tetX3_MK134375	507	696	AMPL3931257	Pool=2
tetX3_MK134375	685	894	AMPL3931258	Pool=1
tetX3_MK134375	883	1110	AMPL3931259	Pool=2
tetX4_MK134376	118	291	AMPL3931249	Pool=1
tetX4_MK134376	280	500	AMPL3931250	Pool=2

tetX4_MK134376	489	680	AMPL3931251	Pool=1
tnpA_JX570731	77	220	AMPL3931691	Pool=2
tnpA_JX570731	209	349	AMPL3931692	Pool=1
tnpA_JX570731	294	489	AMPL3931693	Pool=2
tnpA_JX570731	451	613	AMPL3931694	Pool=1
tnpA_JX570731	601	761	AMPL3931695	Pool=2
tnpA_JX570731	1053	1212	AMPL3931696	Pool=1
tnpA_JX570731	1220	1451	AMPL3931697	Pool=2
tolC_FJ768952	46	138	AMPL3930136	Pool=1
tolC_FJ768952	127	302	AMPL3931824	Pool=2
tolC_FJ768952	291	497	AMPL3931825	Pool=1
tolC_FJ768952	486	706	AMPL3931826	Pool=2
tolC_FJ768952	692	914	AMPL3931827	Pool=1
tolC_FJ768952	903	1084	AMPL3931828	Pool=2
tolC_FJ768952	1073	1261	AMPL3931829	Pool=1
tolC_FJ768952	1250	1449	AMPL3931830	Pool=2
ttgA_AF031417	22	232	AMPL3931831	Pool=1
ttgA_AF031417	221	382	AMPL3931832	Pool=2
ttgA_AF031417	371	585	AMPL3931833	Pool=1
ttgA_AF031417	583	735	AMPL3931834	Pool=2
ttgA_AF031417	723	946	AMPL3931835	Pool=1
ttgA_AF031417	941	1130	AMPL3931836	Pool=2
ttgB_AF031417	57	260	AMPL3931837	Pool=1
ttgB_AF031417	249	416	AMPL3931838	Pool=2
ttgB_AF031417	405	572	AMPL3931839	Pool=1
ttgB_AF031417	560	761	AMPL3931840	Pool=2

ttgB_AF031417	750	969	AMPL3931841	Pool=1
ttgB_AF031417	950	1095	AMPL3931842	Pool=2
ttgB_AF031417	1084	1299	AMPL3931843	Pool=1
ttgB_AF031417	1274	1484	AMPL3931844	Pool=2
ttgB_AF031417	1467	1686	AMPL3931845	Pool=1
ttgB_AF031417	1660	1809	AMPL3931846	Pool=2
ttgB_AF031417	1798	1903	AMPL3931847	Pool=1
ttgB_AF031417	1892	2094	AMPL3931848	Pool=2
ttgB_AF031417	2084	2296	AMPL3931849	Pool=1
ttgB_AF031417	2285	2491	AMPL3931850	Pool=2
ttgB_AF031417	2478	2664	AMPL3931851	Pool=1
ttgB_AF031417	2653	2817	AMPL3931852	Pool=2
ttgB_AF031417	2805	3018	AMPL3931853	Pool=1
ttgB_AF031417	3006	3120	AMPL3930194	Pool=2
vanA_1_FJ866609	59	176	AMPL3931264	Pool=1
vanA_1_FJ866609	165	385	AMPL3931265	Pool=2
vanA_1_FJ866609	374	595	AMPL3931266	Pool=1
vanA_1_FJ866609	584	766	AMPL3931267	Pool=2
vanA_1_FJ866609	754	973	AMPL3931268	Pool=1
vanRC_4_EU151753	50	224	AMPL3931307	Pool=1
vanRC_4_EU151753	213	435	AMPL3931308	Pool=2
vanRC_4_EU151753	424	641	AMPL3931309	Pool=1
vanSC_1_AF162694	32	200	AMPL3931320	Pool=1
vanSC_1_AF162694	189	408	AMPL3931321	Pool=2
vanSC_1_AF162694	397	586	AMPL3931322	Pool=1
vanSC_1_AF162694	575	801	AMPL3931323	Pool=2

vanSC_1_AF162694	790	1016	AMPL3931324	Pool=1
vanSE_AY700375	33	102	AMPL3929173	Pool=1
vanSE_AY700375	91	306	AMPL3931325	Pool=2
vanSE_AY700375	295	413	AMPL3931326	Pool=1
vanSE_AY700375	402	586	AMPL3931327	Pool=2
vanSE_AY700375	575	786	AMPL3931328	Pool=1
vanTE_FJ872411	57	273	AMPL3931339	Pool=2
vanTE_FJ872411	148	372	AMPL3931340	Pool=1
vanTE_FJ872411	364	507	AMPL3931341	Pool=2
vanTE_FJ872411	493	593	AMPL3929205	Pool=1
vanTE_FJ872411	582	793	AMPL3931342	Pool=2
vanTE_FJ872411	781	965	AMPL3931343	Pool=1
vanTE_FJ872411	956	1174	AMPL3931344	Pool=2
vanTE_FJ872411	1163	1364	AMPL3931345	Pool=1
vanTE_FJ872411	1353	1512	AMPL3931346	Pool=2
vanTE_FJ872411	1501	1634	AMPL3931347	Pool=1
vanTE_FJ872411	1623	1837	AMPL3931348	Pool=2
vanTE_FJ872411	1826	2044	AMPL3931349	Pool=1
vatA_1_L07778	30	253	AMPL3930960	Pool=1
vatA_1_L07778	242	460	AMPL3930961	Pool=2
vatA_1_L07778	449	605	AMPL3930962	Pool=1
vatB_1_U19459	27	234	AMPL3930963	Pool=2
vatB_1_U19459	223	379	AMPL3930964	Pool=1
vatB_1_U19459	368	584	AMPL3930965	Pool=2
vatC_1_AF015628	53	252	AMPL3930966	Pool=1
vatC_1_AF015628	241	378	AMPL3930967	Pool=2

vatC_1_AF015628	367	583	AMPL3930968	Pool=1
vatE_AF242872	24	164	AMPL3932038	Pool=2
vatE_AF242872	153	376	AMPL3932039	Pool=1
vatE_AF242872	365	575	AMPL3932040	Pool=2
vgaA_1_M90056	61	233	AMPL3930969	Pool=1
vgaA_1_M90056	222	421	AMPL3930970	Pool=2
vgaA_1_M90056	404	618	AMPL3930971	Pool=1
vgaA_1_M90056	613	807	AMPL3930972	Pool=2
vgaA_1_M90056	790	987	AMPL3930973	Pool=1
vgaA_1_M90056	979	1200	AMPL3930974	Pool=2
vgaA_1_M90056	1189	1291	AMPL3928513	Pool=1
vgaA_1_M90056	1280	1499	AMPL3930975	Pool=2
vgaA_V_AF186237	69	286	AMPL3931422	Pool=1
vgaA_V_AF186237	275	492	AMPL3931423	Pool=2
vgaA_V_AF186237	481	666	AMPL3931424	Pool=1
vgaA_V_AF186237	655	878	AMPL3931425	Pool=2
vgaA_V_AF186237	867	1089	AMPL3931426	Pool=1
vgaA_V_AF186237	1078	1298	AMPL3931427	Pool=2
vgaA_V_AF186237	1287	1504	AMPL3931428	Pool=1
vgaB_U82085	30	144	AMPL3931429	Pool=1
vgaB_U82085	133	359	AMPL3931430	Pool=2
vgaB_U82085	353	552	AMPL3931431	Pool=1
vgaB_U82085	594	742	AMPL3931433	Pool=2
vgaB_U82085	731	947	AMPL3931434	Pool=1
vgaB_U82085	936	1112	AMPL3931435	Pool=2
vgaB_U82085	1090	1195	AMPL3929397	Pool=1

vgaB_U82085	1184	1378	AMPL3931436	Pool=2
vgaB_U82085	1367	1557	AMPL3931437	Pool=1
vgaC_NG_048559	32	217	AMPL3931439	Pool=1
vgaC_NG_048559	206	419	AMPL3931440	Pool=2
vgaC_NG_048559	408	631	AMPL3931441	Pool=1
vgaC_NG_048559	620	794	AMPL3931442	Pool=2
vgaC_NG_048559	783	953	AMPL3931443	Pool=1
vgaC_NG_048559	942	1115	AMPL3931444	Pool=2
vgaC_NG_048559	1104	1309	AMPL3931445	Pool=1
vgaC_NG_048559	1298	1501	AMPL3931446	Pool=2
vgaE_FR772051	92	306	AMPL3931447	Pool=1
vgaE_FR772051	295	509	AMPL3931448	Pool=2
vgaE_FR772051	498	694	AMPL3931449	Pool=1
vgaE_FR772051	683	889	AMPL3931450	Pool=2
vgaE_FR772051	877	1094	AMPL3931451	Pool=1
vgaE_FR772051	1083	1304	AMPL3931452	Pool=2
vgaE_FR772051	1293	1504	AMPL3931453	Pool=1
vgbB_1_AF015628	42	200	AMPL3930976	Pool=2
vgbB_1_AF015628	189	402	AMPL3930977	Pool=1
vgbB_1_AF015628	391	613	AMPL3930978	Pool=2
vgbB_1_AF015628	602	818	AMPL3930979	Pool=1
